# The structural basis for the selectivity of sulfonamido dicarbaboranes toward cancer-associated carbonic anhydrase IX

**DOI:** 10.1080/14756366.2020.1816996

**Published:** 2020-09-23

**Authors:** Michael Kugler, Josef Holub, Jiří Brynda, Klára Pospíšilová, Suzan El Anwar, Dmytro Bavol, Miroslav Havránek, Vlastimil Král, Milan Fábry, Bohumír Grüner, Pavlína Řezáčová

**Affiliations:** a Deparment of Structural Biology, Institute of Organic Chemistry and Biochemistry of the Czech Academy of Sciences, Prague, Czech Republic; b Deparment of Structural Biology, Institute of Molecular Genetics of the Czech Academy of Sciences, Prague, Czech Republic; c Department of Syntheses, Institute of Inorganic Chemistry of the Czech Academy of Sciences, Řež, Czech Republic; d Chemistry Department, Apigenex, s.r.o., Prague, Czech Republic

**Keywords:** Carbonic anhydrase IX, carborane, enzyme inhibitors, structure-activity relationship

## Abstract

Human carbonic anhydrase IX (CA IX), a protein specifically expressed on the surface of solid tumour cells, represents a validated target both for anticancer therapy and diagnostics. We recently identified sulfonamide dicarbaboranes as promising inhibitors of CA IX with favourable activities both *in vitro* and *in vivo*. To explain their selectivity and potency, we performed detailed X-ray structural analysis of their interactions within the active sites of CA IX and CA II. Series of compounds bearing various aliphatic linkers between the dicarbaborane cluster and sulfonamide group were examined. Preferential binding towards the hydrophobic part of the active site cavity was observed. Selectivity towards CA IX lies in the shape complementarity of the dicarbaborane cluster with a specific CA IX hydrophobic patch containing V131 residue. The bulky side chain of F131 residue in CA II alters the shape of the catalytic cavity, disrupting favourable interactions of the spherical dicarbaborane cluster.

## Introduction

Human carbonic anhydrases (CAs) are ubiquitous zinc metalloenzymes that catalyse the reversible hydration of carbon dioxide to generate a bicarbonate anion and proton. This reaction is essential for many physiological processes, such as pH homeostasis, bone development, and electrolyte secretion. Comprehensive experimental evidence also suggests the involvement of CAs in various pathological processes, including diabetes, glaucoma, epilepsy, and, more recently, cancer. To date, fifteen CA isoforms have been identified in humans, and several of them represent established diagnostic and therapeutic targets^1^^,^^2^.

CA IX is a transmembrane isoform whose physiologic expression is very limited and localised to the gastrointestinal tract and stomach^3^. However, CA IX expression is highly upregulated in solid hypoxic tumours exposed to acidic extracellular environments^4^. The reason for the acidic microenvironment is explained by the process known as the Warburg effect^5^. Tumour cells undergo a metabolic shift to the anaerobic glycolytic pathway, which leads to the production and subsequent export of lactic acid^6–10^. The activity of CA IX plays a critical role for tumour viability and progression, and its inhibition results in reduced metastasis, smaller tumours, and slower tumour growth^11–13^.

Lack of selectivity to a specific CA isoform is the major issue of the CA inhibitors currently in use. The challenge in finding an isoform-specific inhibitor arises from the high sequence and structural homology between CA isoforms^14^. Even though the amino acid residues forming active sites of CAs are conserved, there are several residues in the proximity of the active sites, which vary in different isoforms^15^. These residues located further from the catalytic site towards the active site opening differ in charge, hydrophobicity, and shape and can be targeted to design isoform-specific inhibitors^16^^,^^17^.

Various strategies have emerged to overcome the specificity problem^18–20^ and we have recently contributed to this effort by introducing dicarbaborane (with the broadly used trivial name carboranes) clusters as three-dimensional pharmacophores in sulfonamide and sulfamide CA inhibitors^21^^,^^22^. Unlike planar compounds, carboranes possess a three-dimensional pharmacophore providing necessary hydrophobic interactions for filling hydrophobic cavities, and their role in the design of pharmacologically relevant molecules has been firmly established^23–33^. Carboranes are non-toxic abiotic compounds that can increase interaction energy and have good *in vivo* stability and bioavailability^24^^,^^34^.

In our recent study, we explored the inhibitory activity of sulfonamide dicarbaboranes containing 12-vertex *closo* or 11-vertex *nido* carborane clusters with carbon atoms in adjacent position^35^. Variation in inhibitory properties and selectivity towards the cancer-specific CA IX isoform depending on the length of the alkyl linker interconnecting sulfonamide moiety with the carborane cluster was observed.

We conducted a comprehensive structural study to understand the structural basis of compound activity and selectivity towards CA IX. Four additional compounds with extended pentyl and hexyl linkers were synthetised and included in this study. Sixteen X-ray structures were determined to follow compound interactions with the CA IX and CA II active sites, and these provided a structural basis for understanding the structure-activity relationship of sulphonamido carboranes as specific inhibitors of CA IX.

## Materials and methods

### Chemistry

*Nido*-decaborane(14) was purchased from Katchem spol s.r.o., Czech Republic.

The 6,9-(Me_2_S)_2_-*arachno*-B_10_H_12_ was prepared by reaction of the *nido*-decaborane(14) in Me_2_S as the solvent according to a described procedure^36^. Toluene was dried with sodium metal and distilled prior to use. Acetonitrile was dried using 4 Å molecular sieves (Fluka). Other chemicals were purchased from Aldrich, including solvents from Aldrich, Lachema a.s. and Penta s.r.o. in the Czech Republic and used without purification. Analytical TLC was carried out on Silufol^®^ (silica gel on aluminium foil, starch as the binder, Kavalier, Czech Republic). Unless otherwise specified, column chromatography was performed on a high purity silica gel (Merck Grade, Type 7754, 70–230 mesh, 60 Å).

All the reactions were performed with the use of standard vacuum or inert-atmosphere techniques as described by Shriver, although some operations, such as liquid chromatography and crystallizations, were carried out in air. Melting points were determined in sealed capillaries on a BŰCHI Melting Point B-545 apparatus and are uncorrected.

### Instrumental techniques

^1^H and ^11^B NMR spectroscopy was performed on a Varian Mercury 400^Plus^ instrument. The spectra of all the compounds were measured immediately after dissolution. NMR chemical shifts are given in ppm to high frequency (low field) to F_3_B⋅OEt_2_ as the external reference. Residual solvent ^1^H resonances were used as internal secondary standards. Coupling constants ^1 ^*J*(^11^B–^1^H) were measured by resolution-enhanced ^11^B spectra with a digital resolution of 2 Hz and are given in Hz. The NMR data are presented in the text below in the following format: ^11^B NMR: ^11^B chemical shifts δ(^11^B) (ppm), multiplicity, coupling *J*(^11^B–^1^H) constants are given in Hz. Peak assignment is based on {^11^B-^11^B} COSY NMR spectroscopy. ^1^H NMR: chemical shifts δ(^1^H) are given in ppm, coupling constants J(*H,H*) in Hz, δ (^11^B{^11^B}) data are also presented, assignment is based on selectively decoupled δ (^1^H)-{^11^B selective}NMR experiments.

### Mass spectrometry

Mass spectrometry measurements were performed on a Thermo-Finnigan LCQ-Fleet Ion Trap instrument using atmospheric pressure chemical ionisation (APCI, neutral carboranes) or electrospray ionisation (ESI, ionic compounds) techniques. Negative ions were detected. Samples dissolved in acetonitrile (concentrations of approx. 100 ng.ml^−1^) were introduced to the ion source by infusion of 5 μL.min^−1^. Molecular ions [M]^−^ were detected for all univalent anions and [M–H]^–^ for neutral compounds as the base peaks in the spectra. Full agreement of the experimental and calculated isotopic distribution pattern was observed for all these compounds. The isotopic distribution in the boron plot of all peaks is in perfect agreement with the calculated spectral pattern. The data are presented for the most abundant mass in the boron distribution plot (100%) and for the peak corresponding to the *m/z* value.

#### Elemental analyses

Elemental analyses were performed on a Thermo Scientific FlashSmart Organic Elemental Analyser using a V_2_O_5_ catalyst weighted with the sample for combustion of the samples in oxygen. All compounds for EA were dried for 12 h in vacuum at 80 °C before analysis.

#### General procedure used for the synthesis of 1-(sulphonamido)alkyl-1,2-dicarba-closo-dodecaboranes (5a, 6a)

With a syringe, toluene (50 ml) was added to a mixture of the corresponding (C7–C8) alkyne-1-sulfonamide (**A–B**) (3.6 mmol) and 6,9-(Me_2_S)_2_-B_10_H_12_ (0.98 g, 4.0 mmol). The slurry was heated under stirring and refluxed for 24 h. After cooling to room temperature, the solvent was removed under reduced pressure, and products were extracted with diethyl ether (3 × 40 ml). The organic extracts were separated by filtration or decantation, and the combined fractions were evaporated under reduced pressure. The crude products were treated overnight with MeOH (50 ml) acidified with a few drops of HCl (3 M) under stirring. The solvent was then evaporated to dryness. Pure products were isolated by liquid chromatography on a silica gel column (25 × 3.5 cm I.D.) using diethyl ether as a solvent. Fractions containing the product (according to NMR) were combined, evaporated under reduced pressure, and dried in a vacuum.

*1-H_2_NSO_2_(CH_2_)_5_-1,2-closo-C_2_B_10_H_11_ (****5a****).* White solid, yield: 0.66 g (62%); m.p. 106–109 °C. **^11^B NMR (**128 MHz, CD_3_CN, 25 °C, BF_3_.Et_2_O): *δ* = −3.25 d (1B, *J* = 144, *B*(12)H), −6.45 d (1B, *J* = 146, *B*(9)H), −9.97 d (2B, *J* = 150, *B*(8,10)H), −11.68 d, −12.04 d (4B, *B*(3,6,7,11)H), −13.32 d (2B, *J* = 162, *B*(4,5)H); **^1^H {^11^B} NMR** (400 MHz, CD_3_CN, 25 °C, TMS): *δ* = 5.19 brs (2H, N*H*_2_), 4.12 s (1H, C(2)*H*_carb_), 3.00 brt (2H, C*H*_2_S), 2.31 s (1H, B(12)*H*), 2.25 s (4H, B(3,6,7,11)*H*), 2.16 brt (2H, *J* = 7.3 Hz, C*H*_2_), 2.07 s (4H, 4,5,8,10)*H*), 2.02 s (1H, B(9)*H*), 1.72 m (2H, C*H*_2_), 1.48 m (2H, C*H*_2_), 1.37 m (2H, C*H*_2_); **^13 ^C NMR** (100 MHz, CD_3_CN, 25 °C, TMS): *δ* = 77.18 s (1 C, *C*(1)_carb_), 63.22 (1 C, *J* = 195, *C*(2)H_carb_), 55.07 (1 C, *C*H_2_S), 37.38 (1 C, *C*H_2_), 29.31 (1 C, *C*H_2_), 27.81 (1 C, *C*H_2_), 24.08 (1 C, *C*H_2_); **MS: *m/z*** 292.36 (100%), 294.28 (50%), calcd. 292.24 (100%), 294.23 (46%) [M–H]^−^; **Analysis:** Found C 28.32, H 7.54, N 5.14 Calcd. for B_10_C_8_H_25_O_2_NS: C 28.65, H 7.90, N 4.77.

*1-H_2_NSO_2_(CH_2_)_6_–1,2-closo-C_2_B_10_H_11_ (****6a****).* White solid, yield: 0.71 g (64%); m.p. 102–105 °C. **^11^B NMR (**128 MHz, CD_3_CN, 25 °C, BF_3_.Et_2_O): *δ* = −3.26 d (1B, *J* = 150, *B*(12)H), −6.50 d (1B, *J* = 146, *B*(9)H), −9.99 d (2B, *J* = 153, *B*(8,10)H), −11.68 d, −12.10 d (4B, *J* = 168, *B*(3,6,7,11)H), −13.35 d (2B, *J* = 165, *B*(4,5)H); **^1^H {^11^B} NMR** (400 MHz, CD_3_CN, 25 °C, TMS): *δ* = 5.28 brs (2H, N*H*_2_), 4.16 s (1H, C(2)*H*_carb_), 2.99 t (2H, *J* = 8.0, C*H*_2_S), 2.30 s (1H, B(12)*H*), 2.23 brt (2H, *J* = 7.3 Hz, C*H*_2_), 2.14 s (4H, B(3,6,7,11)*H*), 2.06 s (4H, 4,5,8,10)*H*), 2.03 s (1H, B(9)*H*), 1.70 m (2H, C*H*_2_), 1.42 m (2H, C*H*_2_), 1.37 m (2H, C*H*_2_), 1.27 (2H, C*H*_2_); **^13 ^C NMR** (100 MHz, CD_3_CN, 25 °C, TMS): *δ* = 77.34 s (1 C, *C*(1)_carb_), 63.02 (1 C, *J* = 195, *C*(2)H_carb_), 55.22 (1 C, *C*H_2_S), 37.80 (1 C, *C*H_2_), 29.51 (1 C, *C*H_2_), 28.76 (1 C, *C*H_2_), 28.07 (1 C, *C*H_2_), 24.30 (1 C, *C*H_2_); **MS: *m/z*** 306.40 (100%), 308.32 (45%), calcd. 306.25 (100%), 308.25 (46%) [M–H]^−^; **Analysis:** Found C 31.64, H 8.00, N 4.92 Calcd. for B_10_C_8_H_25_O_2_NS: C 31.25, H 8.20, N 4.56.

#### General procedure for the synthesis of potassium salts of 7-(sulfonamido)alkyl-1,2-nido-7,8-dicarbaundecaborates (5 b^−^, 6 b^−^)

MeOH (50 ml) was added to the respective 1-(sulfonamido)alkyl-1,2-dicarba-*closo*-dodecaboranes (**5a** and **6a**; 1 mmol). This was followed by the addition of solid KOH (0.40 g, 10 mmol) under stirring. The methanol solution was stirred and heated at 60 °C for 6 h under reflux. Then water (50 ml) was added and the MeOH was evaporated under reduced pressure. The resulting aqueous solution was diluted with water (60 ml) and extracted with diethyl ether (2 × 20 ml) and ethyl acetate (4 × 25 ml). The ethyl acetate extracts were combined, water (10 ml) was added, and the solvents were evaporated under reduced pressure. The final products were purified by liquid chromatography on a silica gel column (25 × 2.5 cm I.D.) using a CH_2_Cl_2_–CH_3_CN solvent mixture (3:1 to 2:1 b.v.) for elution. Fractions containing the product (according to NMR) were combined, evaporated under reduced pressure, and dried in a vacuum for five hours at 45 °C.

*[7-H_2_NSO_2_-(CH_2_)_5_–7,8-nido-C_2_B_9_H_11_]K (****5 b***^−^*).* White solid, yield: 0.27 g (84%), m. p. 132–134 °C decomp. **^11^B (**128 MHz, CD_3_CN, 25 °C, BF_3_.Et_2_O): *δ* = −11.42 d (2B, *J* = 131, *B*(9,11)H), −14.28 d (1B, *J* = 156, *B*(4)H), −17.51 d −18.86 d (3B, *B*(6)H, *B*(2,5)H), −22.53 d (1B, *J* = 143, *B*(3)H), −33.80 d (1B, *J* = 135, *B*(10)H), −37.65 d (1B, *J* = 137, *B*(1)H); **^1^H {^11^B} NMR** (400 MHz, CD_3_CN, 25 °C, TMS): *δ* = 5.27 s (2H, N*H*_2_), 3.08 brs (2H, C*H*_2_S), 2.44 s (1H, C(8)*H*_carborane_), 1.89 (2H, B(9,11)*H*), 1.78 br .s. (2H, C*H*_2_), 1.69 s (1H, B(4)*H*), 1.66 m (2H, C*H*_2_), 1.50 m (2H, C*H*_2_), 1.41 s (1H, B(3)*H*), 1.40 m (2H, C*H*_2_), 1.11 s (1H, B(6)*H*), 1.02 s (2H, B(2,5)*H*), 0.36 s (2H, B(1)*H*), −0.71 s (1H, B(10)*H*), −2.80 s (1H, µ-*H*); **^13 ^C NMR** (100 MHz, CD_3_CN, 25 °C, TMS): *δ* = 60.48 (1 C, *C*(7)_carb_), 55.27 (1 C, *C*H_2_S), 47.69 (1 C, *C*(8)H_carborane_), 39.76 (1 C, *C*H_2_), 31.35 (1 C, *C*H_2_), 28.77 (1 C, *C*H_2_), 24.45 (1 C, *C*H_2_); **MS (ESI^−^), *m/z***: 283.32 (48%), 284.36 (40%), calcd. 283.23 (100%), 284.23 (52%) [M]^−^, 321.24 (100%), 322.28 (50%), calcd. 321.18 (100%), 322.19 (57%) [M + K–H]^−^; **Analysis** for**:** K**.5b** Found C 26.48, H 7.02, N 4.76, calcd. for B_9_C_7_H_23_O_2_NSK: C 26.13, H 7.21, N 4.35.

*[7-H_2_NSO_2_-(CH_2_)_6_–7,8-nido-C_2_B_9_H_11_]K (****6 b****^−^).* White solid, yield: 0.30 g (90%), m. p. 115–118 °C decomp. **^11^B** (128 MHz, CD_3_CN, 25 °C, BF_3_.Et_2_O): *δ* = −11.45 d (2B, *J* = 134, *B*(9,11)H), −14.30 d (1B, *J* = 156, *B*(4)H), −18.20 d (3B, *B*(6)H, *B*(2,5)H), −22.57 d (1B, *J* = 146, *B*(3)H), −33.80 d (1B, *J* = 135, *B*(10)H), −37.65 d (1B, *J* = 137, *B*(1)H); **^1^H {^11^B} NMR** (400 MHz, CD_3_CN, 25 °C, TMS): *δ* = 5.25 s (2H, N*H*_2_), 3.07 brs (2H, C*H*_2_S), 2.28 s (1H, C(8)*H*_carborane_), 1.91, 1.87 (2H, B(9,11)*H*), 1.77 brs (2H, C*H*_2_), 1.69 s (1H, B(4)*H*), 1.66 m (2H, C*H*_2_), 1.47 m (2H, C*H*_2_), 1.45 s (1H, B(3)*H*), 1.44 m (2H, C*H*_2_), 1.15 m (2H, C*H*_2_), 1.10 s (2H, B(2,5)*H*), 1.07 s (1H, B(6)*H*), 0.36 s (2H, B(1)*H*), −0.06 s (1H, B(10)*H*), −2.78 s (1H, µ–*H*); **^13 ^C NMR** (100 MHz, CD_3_CN, 25 °C, TMS): *δ* = 60.30 (1 C, *C*(7)_carb_), 54.82 (1 C, *C*H_2_S), 47.44 (1 C, *C*(8)H_carborane_), 39.55 (1 C, *C*H_2_), 31.0 (1 C, *C*H_2_), 29.18 (1 C, *C*H_2_), 28.19 (1 C, *C*H_2_), 23.95 (1 C, *C*H_2_); **MS (ESI^−^), *m/z***: 296.32 (100%), 298.32 (40%), calcd. 297.25 (100%), 298.24 (53%) [M]^−^; **Analysis** for**:** K**.6b** Found C 28.92, H 7.76, N 4.45, calcd. for B_9_C_8_H_25_O_2_NSK: C 28.62, H 7.51, N 4.17.

### Biochemistry

#### Protein expression and purification

Recombinant CA II and CA IX-mimic (CA II containing amino acid substitutions A65S, N67Q, E69T, I91L, F131V, K170E, and L204A) were prepared by heterologous expression in *E*. *coli* and purified as previously described^37^. The extracellular part of CA IX comprising the PG and CA domains (residues 38–391) and including the amino acid substitution C174S was expressed in HEK 293 cells and purified as previously described^38^.

#### Inhibition assay

A stopped-flow instrument (Applied Photophysics) was used for measuring the CA-catalysed CO_2_ hydration activity in the presence of inhibitors^39^. The assay buffer consisted of 0.2 mM phenol red (pH indicator used in absorbance maximum of 557 nm), 20 mM HEPES-Na (pH 7.5), and 20 mM Na_2_SO_4_. The concentration of CA II and CA IX in the enzyme assay was 2.5 nM and 0.5 nM, respectively. To stabilise CA IX during measurements, 0.0025% Dodecyl-β-D-maltopyranoside (DDM, Anatrace) was included in the reaction mixture. The substrate (CO_2_) concentration in the reaction was 8.5 mM. Rates of the CA-catalysed CO_2_ hydration reaction were followed for a period of 30 s at 25 °C. Four traces of the initial 5–10% of the reaction were used to determine the initial velocity for each inhibitor. The uncatalyzed rates were determined in the same manner and subtracted from the total observed rates. Stock solutions of inhibitors (100 mM) were prepared in dimethyl sulfoxide (DMSO), and dilutions of up to 100 nM were made thereafter in DMSO. Apparent *K_i_*' values were obtained from dose-response curves recorded for at least six different concentrations of the test compound by the nonlinear least squares method using an Excel spreadsheet fitting the Williams-Morrison equation^40^^,^^41^. *K_i_* values were then derived using the Cheng-Prusoff equation^42^. The *K_M_* values used in the Cheng-Prusoff equation were 9.3 mM for CA II and 7.5 mM for CA IX^43^^,^^44^.

#### Crystallisation and X-ray data collection

Complexes of CA II or CA IX-mimic with compounds were prepared by addition of a onefold to twofold molar excess of the compounds (dissolved in 100% DMSO) to a 20 − 25 mg/ml protein solution in 50 mM Tris-H_2_SO_4_, pH 7.8. The final concentration of DMSO in crystallisation drops did not exceed 10%. Crystals were prepared by the hanging drop vapour diffusion method at 18 °C using EasyXtal^®^ 15-Well Plates (Qiagen). Drops containing 2 μl of the complex solution were mixed with 1 μl of the precipitant solution and then these mixtures were equilibrated over a reservoir containing 1 ml of the precipitant solution. The precipitation solution consisted of 1.6 M sodium citrate, 50 mM Tris-H_2_SO_4_, pH 7.8. Crystals formed within one to three weeks. Prior to data collection, the crystals were soaked for 10 s in the reservoir solution supplemented with 20% (v/v) sucrose and stored in liquid nitrogen. X-ray diffraction data at 100 K were collected on BL14.1 and BL14.2 operated by the Helmholtz-Zentrum Berlin (HZB) at the BESSY II electron storage ring (Berlin-Adlershof, Germany)^45^. Diffraction data were processed using the XDS suite of programs^46^^,^^47^. Crystal parameters and data collection statistics are summarised in Supporting Information Table S1–S2.

#### Structure determination, refinement, and analyses

The crystal structures of both the CA II and CA IX-mimic complexes with compounds were determined by the difference Fourier technique. Coordinates from PDB entries 3PO6^48^ and 6GXE^38^ were used as a models for the CA II and CA IX-mimic complexes, respectively. Atomic coordinates of inhibitor molecules were generated by quantum mechanical (QM) optimizations in the Turbomole package^49^ by means of the density functional theory (DFT) method using the B-LYP functional and the SVP basis set augmented with empirical dispersion correction^50^. The geometric library for the compounds was generated using the Libcheck programme^51^. The Coot programme^52^ was used for inhibitor fitting, model rebuilding, and the addition of water molecules. Refinement was carried out with the Refmac5 programme^53^ with 5% of the reflections reserved for cross-validation. The structures were first refined with isotropic atomic displacement parameters (ADPs). Following addition of solvent atoms and zinc ions, building inhibitor molecules in the active site, and several alternate conformations for a number of residues, anisotropic ADPs were refined for nearly all atoms. Refinement of ADPs was not carried out for spatially overlapping atoms in segments with alternate conformations, or for oxygen atoms of water molecules with an unrealistic ratio of ellipsoid axes. The quality of the crystallographic model was assessed with MolProbity^54^. The final refinement statistics are summarised in Supporting Information Table S1–S2. All the figures representing structures were created using PyMOL^55^. Analysis of contacts was conducted using the Contact programme included in the CCP4 suite^56^. Only contacts between ligands and protein chains up to 4 Å were evaluated. Alternative conformations of ligands were paired with corresponding alternative residues. Interactions were also analysed by PDBePISA^57^, an interactive tool for the exploration of macromolecular interfaces. Only the interaction of the cluster moiety of compounds was evaluated, and only atoms of major (A) conformations of both, the ligand and protein, were used.

Atomic coordinates and structure factors for the crystal structures of CA II in complex with **2a**, **3a**, **4a**, **4 b**^−^, **5a**, **5 b**^−^, **6a**, and **6 b**^−^ were deposited in the PDB with accession codes 6YZV, 6YZT, 6YZQ, 6YZR, 6YZS, 6YZU, 6YZW, and 6YZX, respectively. Atomic coordinates and structure factors for the crystal structures of CA IX-mimic in complex with **1a**, **2a**, **4a**, **4 b**^−^, **5a**, **5 b**^−^, **6a**, and **6 b**^−^ were deposited in the PDB with accession codes 6YZL, 6YZJ, 6YZN, 6Z04, 6YZK, 6YZM, 6YZO, and 6YZP, respectively.

## Results and discussion

### Compound design and synthesis

**Scheme 1.**
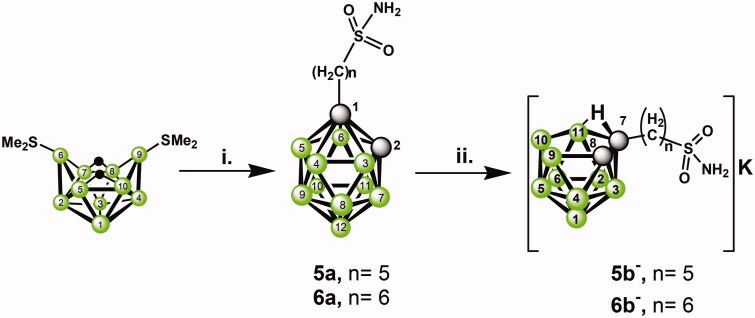
Reaction scheme leading to *closo* and *nido* clusters substituted by alkylsulfonamide groups, i. HCC-(CH_2_)_n_-S(O)_2_NH_2_ (*n* = 5, **I;** *n* = 6, **II**), toluene, reflux, ii. KOH/MeOH, reflux.

The series of sulphonamido carboranes contained a sulphonamidoalkyl group attached to alternative clusters: 1,2-dicarba-*closo*-dodecaborane (here referred to as *closo* clusters) or 7,8-dicarba-*nido*-undecaborate(1-) (here referred to as *nido* clusters). Compounds containing an alkyl linker with a length of *n* = 1 to *n* = 4 (compounds **1a**–**4a** and **1 b**^−^–**4b**^−^) were designed and synthetised in our previous work^35^. Synthesis of compounds with a longer linker length of *n* = 5 and *n* = 6 between the sulfonamide group and the carborane cluster (compounds **5a**–**6a** and **5 b**^−^–**6b**^−^) was carried out using a procedure analogous to the one used for the carboranes with shorter linkers (Scheme 1). The respective sulphonamido acetylenes were inserted into open-cluster 6,9-(Me_2_S)-*arachno*-decaborane(12) producing sulphonamidoalkyl 1,2-dicarba-*closo*-dodecaboranes **5a** and **6a**, respectively. Using a solution of KOH in methanol, these were degraded to the respective 7-substituted derivatives of eleven vertex [7-NH_2_SO_2_-(CH_2_)_n_-7,8-*nido*-C_2_B_9_H_12_]^−^ ions **5 b**^−^ and **6 b**^−^ (*n* = 5 and 6, resp.).

### Carbonic anhydrase inhibition

Compounds **1a,b**^−^ to **6a,b**^−^ were tested for their inhibitory potency *in vitro* by using a stopped-flow carbon dioxide hydration assay. To determine their selectivity, all the compounds were screened not only for inhibition of cancer-associated CA IX but also of widespread CA II (Table 1). CA II is highly expressed in red blood cells and is essential for blood pH regulation. Inhibition of this physiologically important CA isoform would thus cause severe side effects.

**Table 1.** *In vitro* inhibition and X-ray structure overview.

Inhibitor properties			*In vitro* inhibition			X-ray structure resolution (PDB code)	
Cmpd	Cluster type	Linker length	K_i_ (CA II) [nM]	K_i_ (CA IX) [nM]	Selectivity index^a^	CA II	CA IX-mimic
**1a**	*closo*	1	494.4 ± 113.3*^b^*	1136 ± 140.7*^b^*	0.44	N.D.	1.20 Å, (6YZL)
**2a**	*closo*	2	21.0 ± 2.3*^b^*	22.8 ± 3.4*^b^*	0.92	1.65 Å, (6YZV)	1.20 Å (6YZJ)
**3a**	*closo*	3	622.0 ± 177.4*^b^*	0.506 ± 0.11*^b^*	1229.3	1.05 Å (6YZT)	1.20 Å (6T7U^c^)
**4a**	*closo*	4	870.1 ± 91.04*^b^*	3.65 ± 0.64*^b^*	238.4	1.04 Å (6YZQ)	0.95 Å (6YZN)
**5a**	*closo*	5	1296 ± 122.1	14.83 ± 1.98	87.4	1.05 Å (6YZS)	0.99 Å (6YZK)
**6a**	*closo*	6	821.2 ± 70.7	44.65 ± 4.86	18.4	1.03 Å (6YZW)	1.50 Å (6YZO)
**1b**^−^	*nido*	1	63007 ± 28409*^b^*	6545 ± 1625*^b^*	9.6	N.D.	N.D.
**2b**^−^	*nido*	2	60.69 ± 22.36*^b^*	45.16 ± 5.73*^b^*	1.34	N.D.	N.D.
**3b**^−^	*nido*	3	1546 ± 385.9*^b^*	1.178 ± 0.12*^b^*	1312.4	N.D.	1.12 Å (6T9Z^c^)
**4b**^−^	*nido*	4	333 ± 36.59*^b^*	1.605 ± 0.38*^b^*	207.5	1.20 Å (6YZR)	1.05 Å (6Z04)
**5b**^−^	*nido*	5	299.4 ± 25.96	10.63 ± 1.37	28.2	1.00 Å (6YZU)	1.50 Å (6YZM)
**6b^−^**	*nido*	6	29.48 ± 3.32	29.27 ± 7.39	1.01	1.10 Å (6YZX)	1.30 Å (6YZP)

^a^The selectivity index is the ratio between Ki (CA II) and Ki (CA IX).

^b^*K_i_* values from Dvořanová et al., 2020^35^.

^c^Structure reported in Dvořanová et al., 2020^35^; N.D. not determined.

Inhibitor activity of compounds **1a,b**^−^ to **6a,b**^−^ against both CA IX and CA II varied from micromolar to low nanomolar *K_i_* values (Table 1). Compounds **3a** and **3 b**^−^ containing a propyl linker were the most effective inhibitors of CA IX with low or subnanomolar *K_i_* values. CA II was most effectively inhibited by compound **2a** containing a *closo* carborane connected to sulfonamide by an ethyl linker and compound **6 b**^−^ containing a *nido* cluster connected to sulfonamide by a hexyl linker.

**Figure 1.**
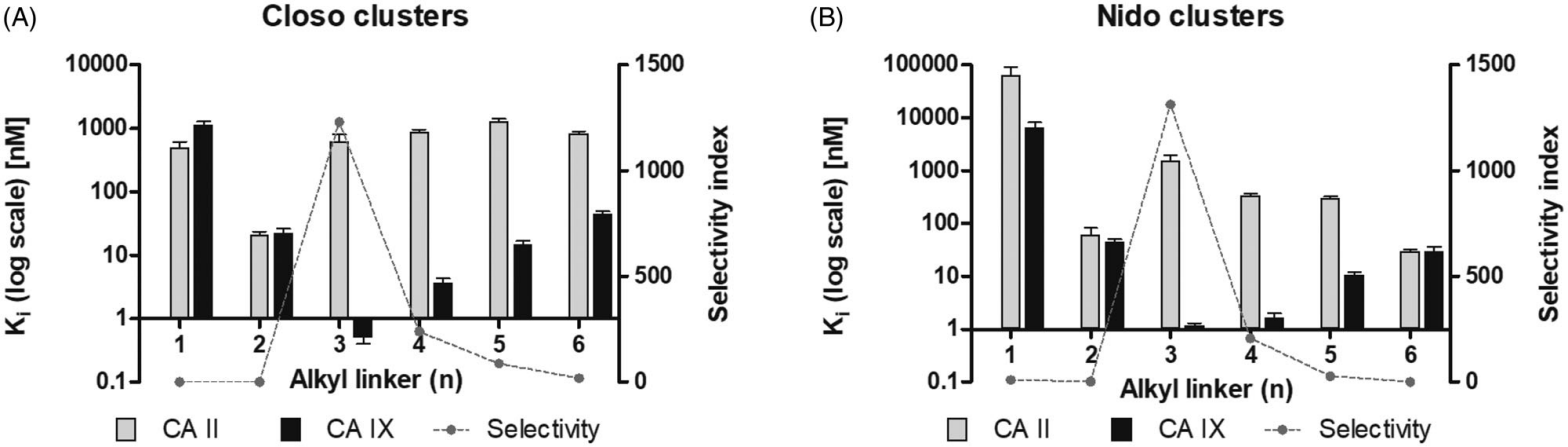
Inhibition and selectivity profiles of compounds **1a** to **6a** (A) and **1 b^−^** to **6 b^−^** (B). K_i_ values (in decadic logarithmic scale) for *in vitro* inhibition are shown in grey for CA II and black for CA IX. Error bars are depicted as standard deviations of a least square statistical analysis of the inhibition curve. The selectivity index shown as a dashed line is the ratio between K_i_ (CA II) and K_i_ (CA IX).

Except for **1a**, **2a,** and **6 b**^−^, the compounds were selective towards CA IX. As the linker length increased from methyl to propyl, selectivity towards CA IX became more favourable, reaching its maxima for **3a** and **3 b**^−^ (Figure 1). Introduction of a longer linker, butyl to hexyl, decreased the CA IX selectivity.

### Structure description and quality

To understand the structural basis of inhibitory potency and selectivity, we set out to obtain structural information on the interactions of all compounds with the active site of CA II and CA IX. We co-crystallized compounds with CA II and CA IX-mimic.

CA IX-mimic is an engineered variant that is widely used in structural studies to resemble hardly crystallisable CA IX. CA IX-mimic contains seven amino acid substitutions (A65S, N67Q, E69T, I91L, F131V, K170E, and L204A) in the CA II active site to mimic the active site of CA IX while retaining good crystallisation properties^37^^,^^58^. We determined 18 co-crystal structures, mostly at atomic resolution (Table 1). Despite extensive crystallisation efforts, we were unable to successfully co-crystallize CA II in complex with **1a**, **1 b**^−^, **2 b**^−^, and **3 b**^−^ or CA IX-mimic with **1 b**^−^ and **2 b**^−^.

**Figure 2.**
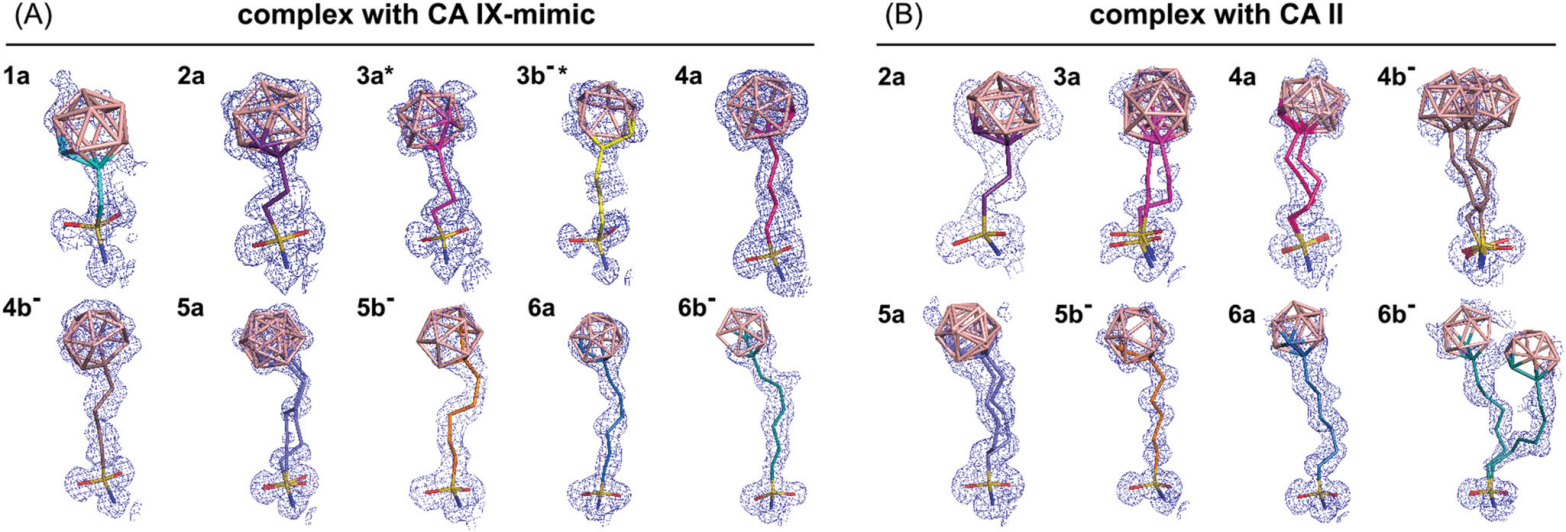
Compounds bound to the active site of CA IX-mimic (A) and CA II (B). *2F_o_-F_c_* map contoured at 1σ is shown with the exception of **4 b^−^**, **5a**, **6a**, and **6 b^−^** bound to CA II that are contoured at 0.5 σ. (A) Compound **5a** was modelled in two alternative conformations with partial occupancies of 0.5 and 0.5. All other compounds were modelled in one conformation with full occupancy. *Crystal structures of **3a** and **3 b^−^** in complex with CA IX-mimic, published previously^35^, are shown for comparison. (B) Compound **3a** was modelled in two alternative conformations with partial occupancies of 0.8 and 0.2. Compound **4 b^−^** was modelled in three alternative conformations with partial occupancies of 0.3, 0.3, and 0.3. Compounds **4a**, **5a**, and **6 b^−^** were modelled in two alternative conformations with partial occupancies of 0.5 and 0.5. All other compounds were modelled in one conformation with full occupancy.

Although the quality of electron density varied, we were able to unambiguously model the compounds in the active sites of CA IX-mimic and CA II (Figure 2). Electron density maps for CA II bound inhibitors were, in general, more difficult to interpret due to static disorder resulting in the modelling of two or three alternative conformations (Figure 2(B), compounds **3a**, **4a**, **5a**, **4 b**^−^, and **6 b**^−^).

**Figure 3.**
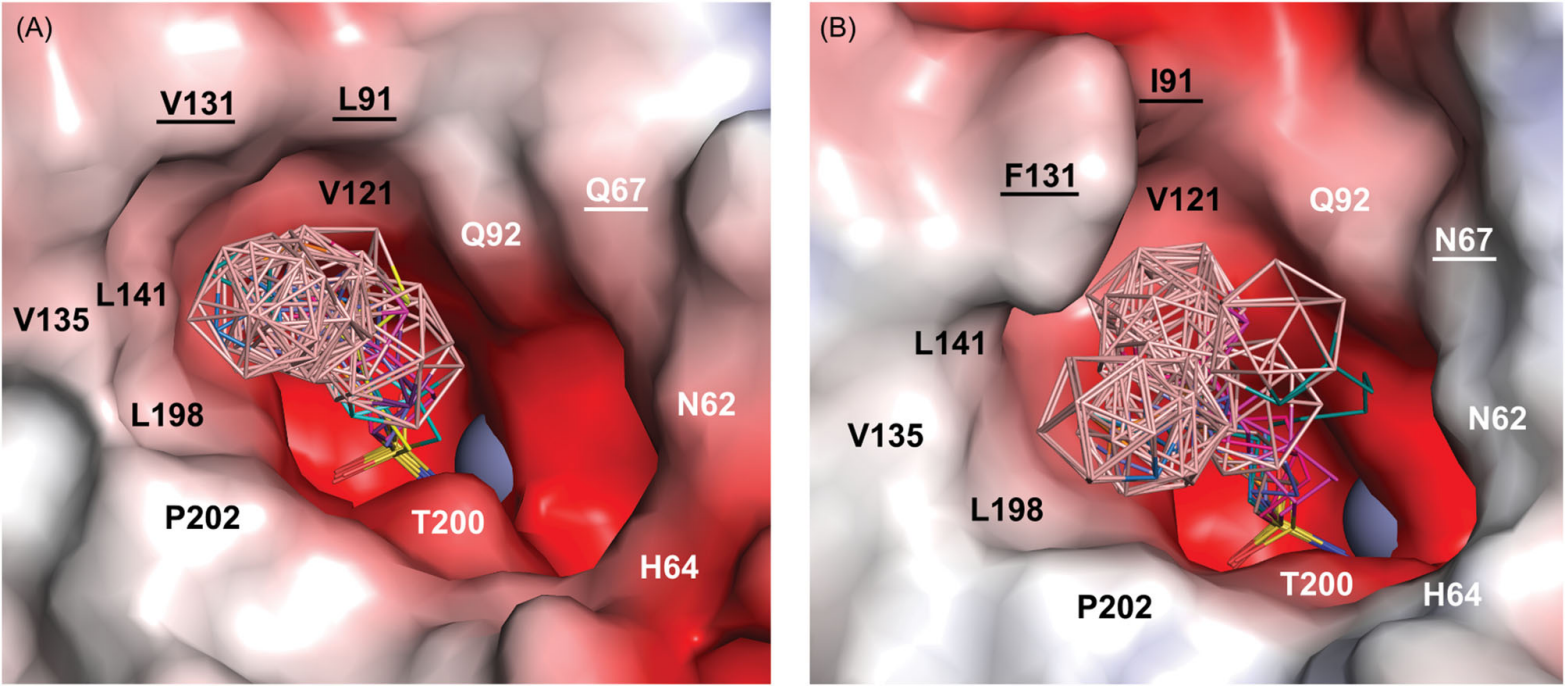
Overview of binding of selected compounds in the active site of CA IX-mimic (A) and CA II (B). The protein surface is represented by its solvent accessible surface coloured by electrostatic potential (red for negative, blue for positive). Compounds are shown as lines with differently coloured carbon atoms: **1a** (cyan), **2a** (purple), **3a** (light magenta), **3 b^−^** (yellow), **4a** (hot pink), **4 b^−^** (brown), **5a** (slate), **5 b^−^** (orange), **6a** (marine), and **6 b^−^** (deep teal). Boron atoms are coloured pink; oxygen, sulfur, and nitrogen are shown in red, yellow, and blue, respectively. Hydrophobic and hydrophilic residues are labelled in black and white, respectively. Residues that vary between CA IX-mimic and CA II are underlined. For clarity, residues H94, H96, H119, V143, and T199 at the bottom of the cavity are not labelled. The zinc ion is represented by the grey sphere.

Superposition of binding poses of all the compounds bound to the CA IX and CA II active site revealed that the position of the sulfonamide moiety is invariant and that compounds differ in linker conformations and cluster positions (Figure 3, Supporting Information Figure S1). The sulfonamide moiety is well anchored to the enzyme active site through interaction with the zinc ion. As previously observed for other sulfonamide complexes, binding occurs directly between the active site zinc ion and the N1 atom of the sulfonamide group^59^. The zinc ion is coordinated by three conserved histidine residues, H94, H96, and H119 (CA II numbering will be used throughout the manuscript), and as the fourth ligand serves the sulfonamide moiety instead of the bound solvent. sulfonamide complexes also provide additional hydrogen bonding between the sulfonamide nitrogen N1 and OG of T199 as well as between the sulfonamide oxygen O1 and NH of T199.

The aliphatic linker acquired extended conformation and guided the carborane cluster towards the opening of the CA IX or CA II active site (Figure 3). In the CA IX active site, we observed preferential binding of all clusters to the hydrophobic part of the active site formed by L91, V121, V131, V135, L141, V143, L198, and P202 (Figure 3). For CA II, we also observed preferential binding of carborane clusters towards a hydrophobic patch formed by residues I91, V121, F131, V135, L141, L198, and P202; however, alternative binding conformations of several compounds (**3a**, **4a**, **5a**, **4 b**^−^, and **6 b**^−^) were oriented towards the hydrophilic part of the active site opening formed by N62, H64, N67, Q92, T199, and T200 (Figure 3).

### Interaction of compounds within CA IX and CA II active sites

**Figure 4.**
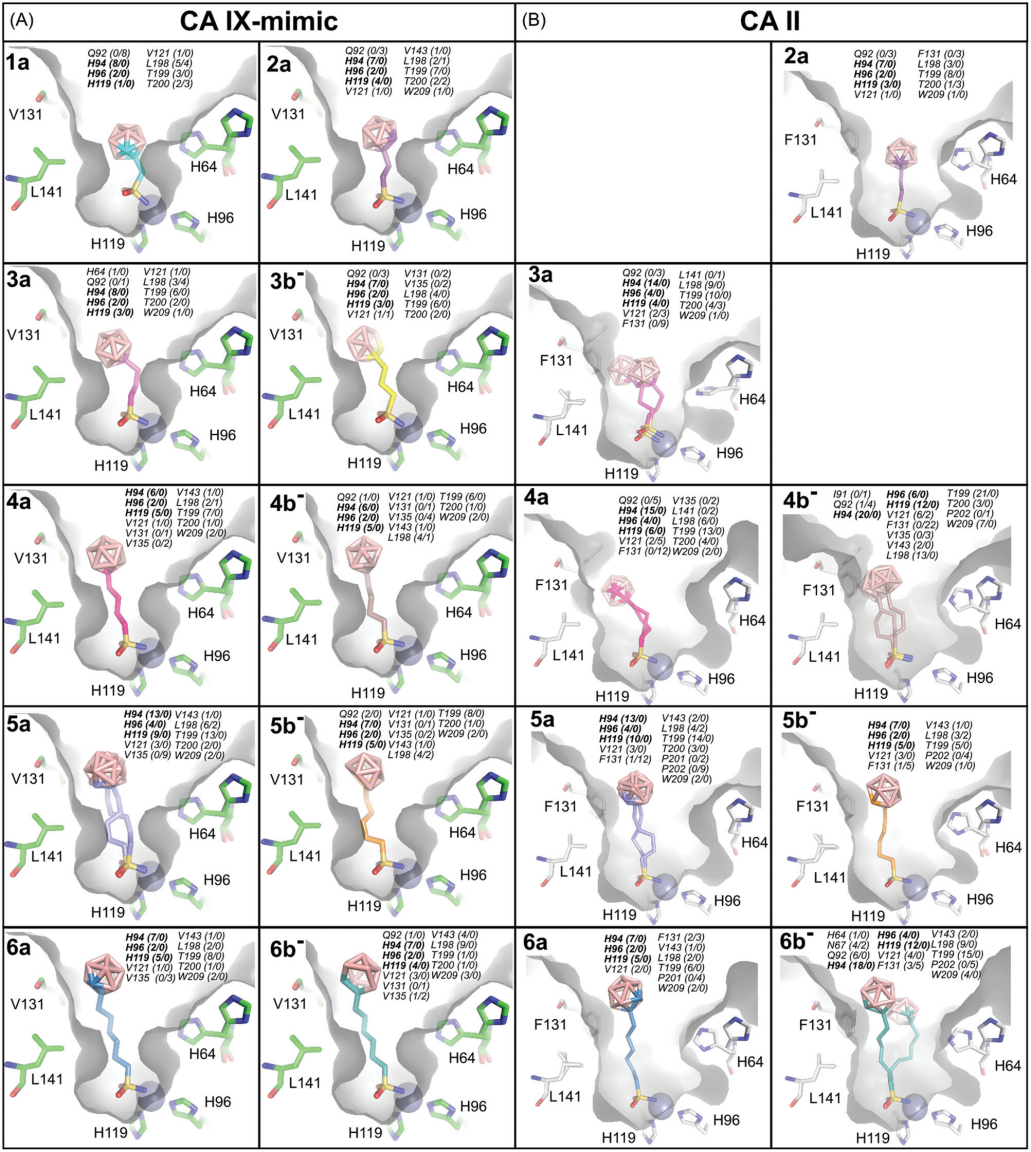
The structures of compounds bound to the CA IX-mimic cavity (A) and CA II (B). Structures with **1a** and **3 b^−^** in complex with CA II were not determined. The compounds are depicted in stick representation with differently coloured carbon atoms: **1a** (cyan), **2a** (purple), **3a** (light magenta), **3 b^−^** (yellow), **4a** (hot pink), **4 b^−^** (brown), **5a** (slate), **5 b^−^** (orange), **6a** (marine), and **6 b^−^** (deep teal). Boron atoms are coloured pink; oxygen, sulfur, and nitrogen are shown in red, yellow, and blue, respectively. Protein cavity is shown as a grey surface, zinc ion is represented by the grey sphere. Only residues H64, H96, H119, V131, and L141 forming part of active site cavity are highlighted as sticks; other residues are omitted for clarity. All contacts with distance between the ligand and protein atoms less than or equal to 4 Å are listed for each compound. The first number in parentheses represents the number of contacts with the compound’s linker and the sulfonamide moiety, while the second number represents the number of contacts with the carborane cluster. The residues highlighted in bold represent conserved residues (H94, H96, H119) involved in zinc ion coordination.

To understand in detail the interactions of the compounds inside the CA IX-mimic cavity, we analysed interatomic contacts between the compounds and the interacting residues (Figure 4). Methyl and ethyl linkers (**1a** and **2a**) did not allow carborane clusters to make favourable hydrophobic interactions with hydrophobic residues due to their short length. On the contrary, butyl, pentyl, and hexyl linkers (**4a**, **5a**, **6a**, **4 b**^−^, **5 b**^−^, and **6 b**^−^) extended the carborane cluster much further beyond the pocket. Propyl linkers had an ideal length to accommodate the cluster in the hydrophobic cleft formed by V121, V131, V135, and L198. Both compounds possessing the propyl linker (**3a** and **3 b**^−^) thus showed the highest inhibitory potency towards CA IX.

Similarly, we analysed the interaction interface of the compounds bound in the CA II cavity (Figure 4). The ethyl linker in **2a** positioned the cluster in the centre of the CA II active site cavity. The cluster interacts with both the hydrophobic and the polar sides of the active site cavity, and this can explain the highest potency of the compound towards CA II. Longer propyl and butyl linkers (**3a**, **4a**, **4 b**^−^) allowed hydrophobic interactions with I91, V135, L141, and P202. This interaction was accompanied by a remodelling of the hydrophobic side chains, specifically the flip of F131 from the original rotameric position (t80) to a less favourable one (p90). The pentyl and hexyl linkers (**5a**, **5 b**^−^, **6a**, and **6 b**^−^) allowed carborane clusters to interact with residues at the opening of the active site (P201 and P202) and no flip of F131 was observed.

### Structural basis for CA IX selectivity

**Figure 5.**
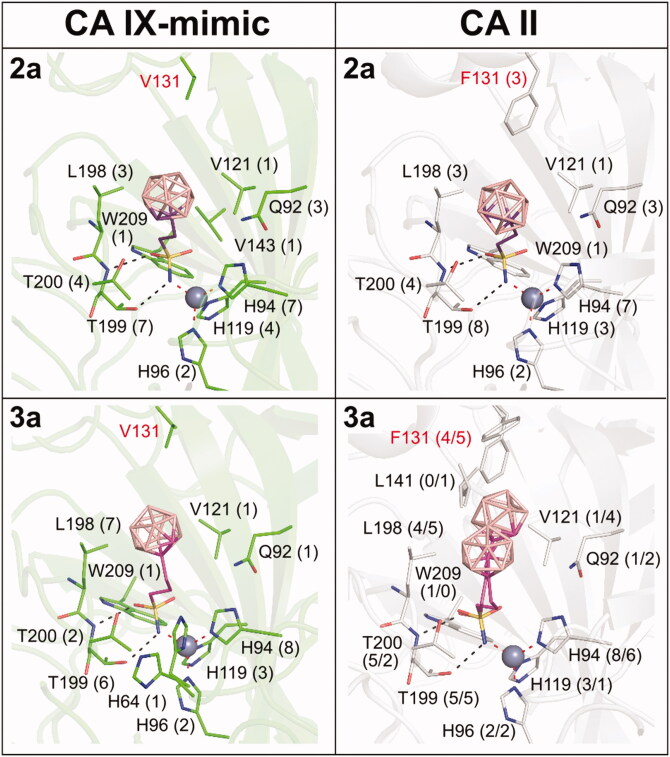
The different binding positions of compounds between the CA IX-mimic and CA II active sites. The structures of compounds are depicted as sticks with differently coloured carbon atoms: **2a** (purple) and **3a** (light magenta). Boron atoms are coloured pink; oxygen, sulfur, and nitrogen are shown in red, yellow, and blue, respectively. Carbon atoms in CA IX-mimic and CA II are shown in green and white, respectively. Protein is represented as cartoon with the residues interacting with compounds highlighted as sticks. The zinc ion is shown as the grey sphere. Polar contacts between protein and compound are labelled as black dashed lines, and coordination bonds are marked as red dashed lines. The numbers in parentheses give the number of contacts with a distance between the ligand and protein atoms less than or equal to 4 Å. The numbers are separated by a slash to express the number of contacts for each alternative conformation. The active site residues that differ between CA IX-mimic and CA II are highlighted in red.

Detailed comparison of binding poses and interactions of compounds with CA IX and CA II provided the structural basis for explaining the selectivity towards CA IX (Supporting Information Table S3–S4). Seven amino acid substitutions distinguish the CA IX-mimic active site from that of CA II, and three of them (N67Q, I91L, and F131V) were in contact with at least one compound in our crystal structures (Figure 5, Supporting Information Figure S2). Residues N67 and I91 only contacted **4 b**^−^ and **6 b**^−^, respectively. On the contrary, residue 131 was involved in interaction with the majority of compounds. The F131V substitution significantly alters the shape of the hydrophobic patch in the CA active site. The change from the bulky F131 in CA II to the rather small V131 in CA IX led to more open access to the active site and the formation of a concave hydrophobic pocket^60^^,^^61^. We believe that this substitution is crucial for the selectivity of carborane sulfonamide s towards CA IX, as this pocket can be efficiently filled by the three-dimensional carborane cluster.

The shape complementarity and fit of the cluster into the concave hydrophobic pocket can be documented by the solvent accessible area, which is buried upon complex formation (Table 2).

**Table 2.** Analysis of cluster interaction with the CA active site^a^.

Cmpd	CA IX-mimic			CA II		
	ASA^b^ [Å^2^]	BSA*^c^* [Å^2^]	BAP*^d^* [%]	ASA^b^ [Å^2^]	BSA*^c^* [Å^2^]	BAP*^d^* [%]
**1a**	274.72	208.85	76.0	N.D.	N.D.	N.D.
**2a**	271.11	204.05	75.3	271.13	202.43	74.7
**3a**	272.49	202.35	74.3	273.48^e^	184.03^e^	67.3^e^
**3b**^−^	266.99	180.20	67.5	N.D.	N.D.	N.D.
**4a**	272.00	165.44	60.8	272.55^e^	189.18^e^	69.4^e^
**4b**^−^	270.35	165.73	61.3	268.95^e^	152.98^e^	56.9^e^
**5a**	270.65^e^	150.27^e^	55.5^e^	272.85^e^	129.49^e^	47.5^e^
**5b**^−^	270.45	155.11	57.4	268.09	129.26	48.2
**6a**	272.94	124.19	45.5	273.48	118.75	43.4
**6b**^−^	271.14	119.97	44.2	269.03^e^	120.89^e^	44.9^e^

^a^The solvent accessible area of the carborane cluster, which is buried upon complex formation, was calculated using PDBePISA^57^.

^b^The accessible surface area (ASA) for the respective cluster; ^c^the buried surface area (BSA) for the respective cluster; ^d^the buried area percentage (BAP) is the ratio between ASA and BSA expressed in a percentage; N.D.: not determined.

^e^Only major conformations (A) of compounds were subjected to interface analysis.

The buried area percentage (BAP) for the carborane cluster is generally larger for CA IX complexes. The decrease in the BAP was observed as the linker became longer and clusters formed interactions with residues located further away from the catalytic site. The largest extent of buried surface area was observed for compounds with short linkers. Clusters of **1a** and **2a** were buried deeply within the active sites of CA IX and CA II in a similar manner, not reaching the hydrophobic patch at the active site opening. These compounds have no selectivity towards CA IX (Table 1). The propyl linker of **3a** in CA IX-mimic allowed the cluster to fill the hydrophobic cavity in proximity to V131, keeping its high BAP (74.3%). On the contrary, the bulkiness of F131 caused displacement of the **3a** cluster towards the centre of the CA II active site, leading to a significant decrease in the BAP (67.3%). Extension of the linker caused a drop of the BAP to 60.8% for **4a** in CA IX-mimic as the cluster bypassed the selectivity pocket and positioned itself towards the active site opening. Contrarily, the **4a** cluster in CA II forced F131 to maintain a single rotameric orientation forming an interface for cluster accommodation with BAP values similar to those of **3a**. Interactions with other residues that distinguish the CA IX active site from that of CA II (N67Q and I91L) are quite limited: **4 b**^−^ in complex with CA II interacted with I91, and **6 b**^−^ in complex with CA II interacted with N67 and I91. Due to the alternative binding conformations of **3a**, **4 b**^−^, and **6 b**^−^ in the CA II active site, several conformation-specific interactions were observed: interactions with L141 and W209 for **3a**; interactions with I91, V135, T200, and P202 for **4 b**^−^; and interactions with H64, N67, and P202 for **6 b**^−^. Otherwise, the list of the interacting residues was comparable between CA II and CA IX-mimic (Figure 5, Supporting Information Figure S2).

In conclusion, the high inhibitory activity and selectivity of compounds **3a** and **3 b**^−^ towards CA IX is due to the binding of the carborane cluster to the CA IX specific pocket lined by residues L91, V131, and L141. The bulky side chain of residue F131 in CA II does not provide shape complementarity for favourable interactions of a spherical carborane cluster. Less favourable interaction thus resulted in alternative binding conformations of compounds inside the CA II active site.

### Binding of closo vs. nido clusters

**Figure 6.**
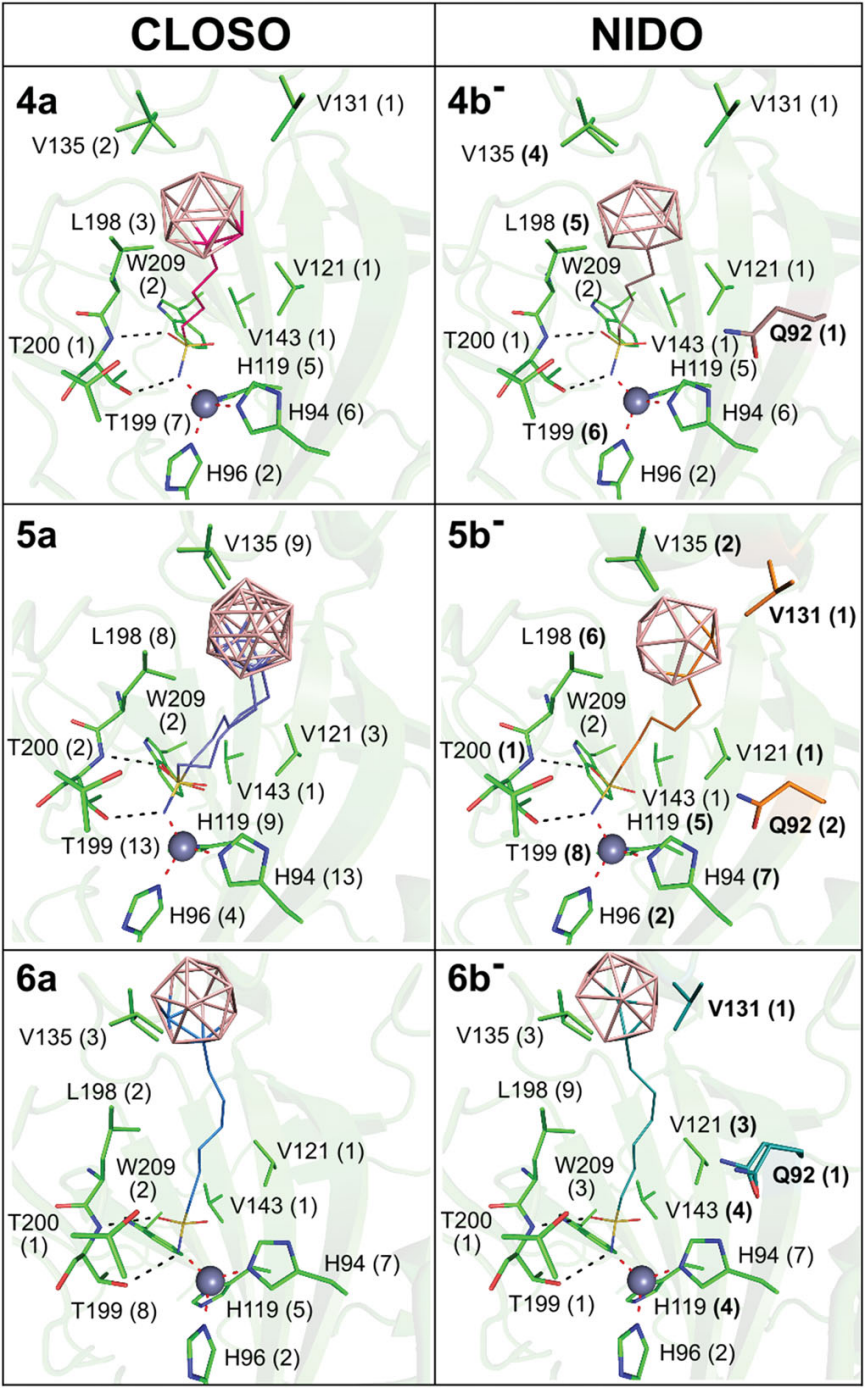
Binding positions of compounds containing the *closo* and *nido* cluster, respectively, in the CA IX-mimic active site. The compounds are depicted in lines with carbon atoms coloured differently: **4a** (hot pink), **4 b^−^** (brown), **5a** (slate), **5 b^−^** (orange), **6a** (marine), and **6 b^−^** (deep teal). Protein is shown in green cartoon representation and interacting residues are highlighted as sticks. The zinc ion is represented by the grey sphere. Polar contacts are represented as black dashed lines, and coordination bonds are marked as red dashed lines. The numbers in parentheses give the number of contacts with a distance between the ligand and protein atoms less than or equal to 4 Å. Changes in interaction between the corresponding *closo* (**a**) and *nido* (**b^−^**) cluster-containing compounds are in bold, and additional interacting residues are highlighted by carbon colour corresponding to the one of the interacting compound.

We analysed differences between the interactions provided by the two types of carborane clusters present in our series. The *closo* cluster consists of ten boron and two carbon atoms, while one boron atom is absent for the *nido* cluster, resulting in the overall negative charge of the *nido* cluster. We compared binding between the *closo* (compounds **4a**, **5a**, **6a**) and *nido* clusters (**4 b**^−^, **5 b**^−^, **6 b**^−^) in the CA IX-mimic active site (Figure 6). We described minor differences in binding between **3a** and **3 b**^−^ in a previous study^35^ and the differences between the compounds containing *closo* and *nido* clusters were also quite subtle. The positions of the two corresponding pairs of compounds **4a**/**4b**^−^ and **6a**/**6b**^−^ in the CA IX active site were almost identical. The RMSD value for superposition of 19 atoms of **4a** and **4 b^−^** and 21 atoms of **6a** and **6 b^−^** was 0.228 Å and 0.204 Å, respectively. In addition, the interacting residues are almost identical, with the exception of two additional residues (Q92 and V131) interacting with the *nido* clusters of **4 b^−^** and **6 b^−^** (Figure 6). A more noticeable difference was found between the binding modes of compounds **5a** and **5 b^−^**. The RMSD value **5a** and **5 b^−^** was 0.653 Å and 0.571 Å (20 atoms superposed) for the two alternative conformations of **5a**, respectively. Interaction of **5 b^−^** with the two additional residues (Q92 and V131, Figure 6) might be the reason for the presence of a single binding conformation of **5 b^−^**.

We might conclude that even though the differences between the interactions of the *closo* and *nido* clusters with the CA IX active site residues are mostly minor in comparison with the changes caused by variation in the linker length, they still managed to cause distinguishable differences in the compound inhibitory activities.

## Conclusion

We have previously showed that carborane clusters present a useful three-dimensional pharmacophore in the design of CA IX inhibitors^21^^,^^22^. In this study, we explored a series of compounds comprising two types of carborane clusters: 12-vertex 1,2-dicarba-*closo*-dodecaborane and 11-vertex 7,8-dicarba-*nido*-undecaborate (dicarbollide) connected to a sulfonamide moiety *via* aliphatic linkers of varying lengths (1 to 6 carbon atoms; *n* = 1–6). The length of the linker strongly affected compound inhibitory potency and selectivity towards the cancer-specific CA IX isoform.

We conducted a detailed structural study focussed on compound interactions with active sites of the cancer-specific isoform CA IX and widely physiologically expressed isoform CA II to identify the structural basis for high potency and selectivity. Eighteen X-ray structures of compounds bound to CA IX-mimic or CA II revealed the preferential interaction of carborane clusters with the hydrophobic patch around residue 131 in the CA active site opening. The sequence of CA II and CA IX differs in this region (bulky F131 in CA II versus small V131 in CA IX), and thus interaction of carborane clusters with this region determines selectivity towards CA IX. Optimal filling of the CA IX-mimic hydrophobic cavity (containing the V131 residue) by carborane clusters was observed for compounds with propyl linkers (**3a**,**b^−^**).

Our results presented herein might serve as the structural basis for further development of potent and specific inhibitors of CA IX as potential anti-tumour agents.

## Supplementary Material

Supplemental Material
