# Supplementary material for: The structural basis for the selectivity of sulfonamido dicarbaboranes toward cancer-associated carbonic anhydrase IX
Source: J Enzyme Inhib Med Chem. 2020 Sep 23;35(1):1800–10. doi: 10.1080/14756366.2020.1816996 (PMC7534198; doi:10.1080/14756366.2020.1816996)
Supplement: Supplemental Material [file IENZ_A_1816996_SM0975.pdf]

Supporting information

The structural basis for the selectivity of sulfonamido dicarbaboranes towards cancer-associated carbonic anhydrase IX

Michael Kugler[a,b], Josef Holub[c], Jiří Brynda[a,b], Klára Pospíšilová[a], Suzan El Anwar[c], Dmytro Bavol[c], Miroslav Havránek[d], Vlastimil Král[b], Milan Fábry[b], Bohumír Grüner[c]* and Pavlína Řezáčová[a,b]*

Chemical synthesis……2

Table S1. Diffraction data collection and refinement statistics of CA IX-mimic complexes……6

Table S2. Diffraction data collection and refinement statistics of CA II complexes……8

Table S3. List of contacts between compounds and CA IX-mimic……10

Table S4. List of contacts between compounds and CA II……11

Figure S1. Superposition of compounds in CA IX-mimic and CA II active sites……12

Figure S2. Binding positions of compounds in between CA IX-mimic and CA II active sites……13

Supplementary references……14

NMR spectra of compounds **5a**, **5b**$^-$, **6a** and **6b**$^-$……15

5a 1-$NH_2S(O)_2C_5H_{10}$-*closo*-1,2-dicarbaborane……16

6a 1-$NH_2S(O)_2C_6H_{12}$-*closo*-1,2-dicarbaborane……23

5b$^-$ [7-$NH_2S(O)_2C_5H_{10}$-*nido*-7,8-$C_2B_9H_{11}$]K……30

6b$^-$ [7-$NH_2S(O)_2C_6H_{12}$-*nido*-7,8-$C_2B_9H_{11}$]K……38

Chemical synthesis

Synthesis of sulfonamide alkynes

Both compounds were prepared from the respective alkynyl alcohols, in which structures the alkyne group and terminal hydroxy group were separated by 5 and 6 memberred aliphatic linker. The hydroxyl group was converted to methanesulfonates in the first step. The esters were then reacted with sodium iodide producing iodides that were transferred to sodium sulfonates. The sulfonates were reacted with phosphorus pentachloride to the respective chlorosulfonates, which were not isolated and were reacted with aqueous ammonia in one pot arrangement producing the respective alkynylsulfonamides as the final products.

Hept-6-yne-1-sulfonamide (I)

Hept-6-yn-1-yl methanesulfonate: Methanesulfonyl chloride (9.7 mL, 128 mmol) was added to a solution of hept-6-yn-1-ol (12 g, 106 mmol) and triethylamine (22 mL, 159 mmol) in dichloromethane (150 mL) at 0 °C. The mixture was stirred overnight, then washed with water (1 x 100 mL), 1 M aqueous solution of hydrochloric acid (1 x 100 mL), 5% aqueous solution of sodium bicarbonate (1 x 100 mL), and brine (1 x 100 mL). Organic phase was dried over anhydrous sodium sulfate, filtered, and evaporated giving hept-6-yn-1-yl methanesulfonate; Yield: 18 g (95%); ^{1}H NMR (300 MHz, $CDCl_3$, δ_H): 4.18 t (2H, *J*=6.5 Hz), 2.96 s (3 H), 2.19 - 2.12 m (2 H), 1.92 t (2H, *J*=2.7 Hz), 1.44 - 1.56 m (6 H).

7-Iodohept-1-yne: A mixture of hept-6-yn-1-yl methanesulfonate (18.9 g, 100 mmol) and sodium iodide (16.5 g, 110 mmol) in acetone (180 mL) was stirred for 3 days at room temperature, then refluxed for 5 h. Solvent was evaporated giving 7-iodohept-1-yne;

Yield: 8.22 g (37%); ^{1}H NMR (300 MHz, $CDCl_3$, δ_H): 3.17 t (2H, *J*=7.1 Hz), 2.15 - 2.23 m (2 H), 1.94 t (1H, *J*=2.7 Hz), 1.43 - 1.61 m (6 H).

*Hept-6-yne-1-sulfonamide (**V**):* Phosphorus pentachloride (3.30 g, 15.8 mmol) was added to a mixture of sodium hept-6-yne-1-sulfonate (2.85 g, 14.4 mmol, prepared from 7-iodohept-1-yne according published procedure [1]) in dry dichloromethane (50 mL) at 0 °C. Resulting mixture was refluxed overnight. The solvent was evaporated. The residue was suspended in tetrahydrofuran (50 mL) followed by addition of ammonium hydroxide (25% aqueous solution, 5 mL, 66.2 mmol). The mixture was left to stand overnight, then it was evaporated. The residue was dissolved in ethyl acetate (70 mL) and washed with water (3 x 50 mL). Organic phase was dried over anhydrous sodium sulfate, filtered, and evaporated. The residue was treated with diethyl ether/cyclohexane mixture (1:1) overnight. Solid was filtered, washed with diethyl ether/cyclohexane mixture (1:1), and dried giving the title compound **V** as white solid; Yield: 713 mg (28 %); ^{1}H NMR (600 MHz, $CDCl_3$): δ_H= 4.613 br s (2 H, NH), 3.135 t (2H, CH2, *CH2*SO2), 2.226 brs, (2H, *CH2*C), 1.962 s (1H, CH2), 1.890 q (2H, CH2), 1.579 brs (4H, CH), **^{13}C{1H} NMR** (150 MHz, $CDCl_3$, 25 °C, TMS): 83.85 (1C), 68.737 (1C), 55.126 (1C), 27.776 (1C), 27.149 (1C), 23.515 (1C), 18.096 (1C); **MS ESI$^-$** (*m/z*): found 175.08, calcld. 175.07 $[M]^-$.

Oct-7-yne-1-sulfonamide (II)

Oct-7-yn-1-yl methanesulfonate: Methanesulfonyl chloride (3.62 mL, 46.8 mmol) was added dropwise to a solution of oct-7-yn-1-ol (4.92 g, 39 mmol) and triethylamine (8.13 mL, 58.5 mmol) in dichloromethane at 0 °C. Resulting mixture was stirred for 1.5 hours at 0 °C and 3 hours at room temperature. Water (30 mL) was added. Layers

were separated; the organic one was washed with 1 M aqueous solution of hydrochloric acid (1 x 30 mL), 5% aqueous solution of sodium bicarbonate (1 x 40 mL) and 10% aqueous solution of sodium chloride (1 x 30 mL), dried over anhydrous sodium sulfate, filtered and evaporated giving oct-7-yn-1-yl methanesulfonate as white solid; Yield: 7.96 g (100 %); ^{1}H NMR (300 MHz, $CDCl_3$, δ_H): 4.21 t (2H, *J*=6.5 Hz), 2.99 s (3 H), 1.93 t (1H, *J*=2.7 Hz), 2.22 - 2.14 m (2 H), 1.81 - 1.69 m (2 H), 1.58 - 1.47 m (2 H), 1.47 - 1.37 m (4 H).

8-Iodooct-1-yne: Oct-7-yn-1-yl methanesulfonate (7.05 g, 34.5 mmol) was dissolved in acetone (120 mL) followed by addition of sodium iodide (20.7 g, 138 mmol). Resulting mixture was refluxed overnight. Then, the mixture was filtered; the filtrate was evaporated and purified by vacuum distillation (8 mm/Hg, 94 °C) giving 8-iodooct-1-yne as colorless liquid; Yield: 5.02 g (54%); ^{1}H NMR (300 MHz, $CDCl_3$, δ_H): 3.19 t (2H, *J*=7.0 Hz), 2.24 - 2.16 m (2 H), 1.94 t (1H, *J*=2.7 Hz), 1.90 - 1.77 m (2 H), 1.61 - 1.48 m (2 H), 1.47 - 1.36 m (4 H).

Sodium oct-7-yne-1-sulfonate: A solution of 8-iodooct-1-yne (4.8 g, 20.3 mmol) in methanol (40 mL) and a solution of sodium sulfite (3.84 g, 30.5 mmol) in water (50 mL) were combined, stirred for 30 min at room temperature, and refluxed overnight. Methanol was added and the mixture was filtered. The filtrate was evaporated followed by addition of acetone. The precipitate was filtered giving sodium oct-7-yne-1-sulfonate as white solid; Yield: 2.74 g (63%); ^{1}H NMR (300 MHz, D_2O, δ_H): 2.94 – 2.85 m (2 H), 2.33 t (1H, *J*=2.7 Hz), 2.24 - 2.17 m (2 H), 1.77 - 1.66 m (2 H), 1.58 - 1.47 m (2 H), 1.47 - 1.36 m (4 H).

*Oct 7-yne-1-sulfonamide (**VI**):* Phosphorus pentachloride (2.93 g, 14.1 mmol) was added to a mixture of sodium hept-6-yne-1-sulfonate (2.72 g, 12.8 mmol) in dry dichloromethane (40 mL) at 0 °C. Resulting mixture was refluxed for 5 hours. The solvent was evaporated. The residue was suspended in tetrahydrofuran (40 mL) followed by addition of ammonium hydroxide (25% aqueous solution, 4.5 mL, 59.6 mmol). The mixture was left to stand over weekend, then it was evaporated. The residue was dissolved in ethyl acetate (70 mL) and washed with water (3 x 50 mL). Organic phase was dried over anhydrous sodium sulfate, filtered, and evaporated. The residue was treated with diethyl ether/cyclohexane mixture (1:1) overnight. Solid was filtered, washed with diethyl ether/cyclohexane mixture (1:1), and dried giving the title compound **VI** as white solid; Yield: 460 mg (19 %); ^{1}H NMR (600 MHz, $CDCl_3$): δ_H= 4.81 br s (2 H, *NH*), 3.11 t (2H, *CH2*, *J*= 2.4 Hz, *CH2*SO2), 2.19 t, (2H, *CH2*C), 1.94 t (1H, *CH*, *J*= 8.4 Hz), 1.87 m (2H, *CH2*), 1.53 m (2H, *CH2*), 1.44 brs (4H, *CH2*), **^{13}C{1H} NMR** (150 MHz, $CDCl_3$, 25 °C, TMS): 84.24 (1C), 68.47 (1C), 55.16 (1C), 28.04 (1C), 27.95 (1C), 27.16 (1C), 23.78 (1C), 18.22 (1C); **MS ESI$^-$** (*m/z*): found 189.16, calcld. 189.08 $[M]^-$.

Table S1. Diffraction data collection and refinement statistics of the CA IX-mimic complexes.

**CA IX-mimic in complex with**								
**Ligand**	closo methyl	closo ethyl	closo butyl	nido butyl	closo pentyl	nido pentyl	closo hexyl	nido hexyl
	**1a**	**2a**	**4a**	**4b**$^-$	**5a**	**5b**$^-$	**6a**	**6b**$^-$
**Data collection statistics**								
Space group	$P2_1$	$P2_1$	$P2_1$	$P2_1$	$P2_1$	$P2_1$	$P2_1$	$P2_1$
Cell parameters (Å; °)	41.81<br>41.20<br>72.25<br>90.00<br>103.91<br>90.00	41.68<br>40.91<br>72.17<br>90.00<br>103.84<br>90.00	41.92<br>41.34<br>72.47<br>90.00<br>103.90<br>90.00	42.03<br>41.34<br>72.68<br>90.00<br>103.98<br>90.00	41.84<br>41.25<br>72.37<br>90.00<br>103.96<br>90.00	41.67<br>41.00<br>71.21<br>90.00<br>103.65<br>90.00	41.86<br>41.07<br>72.35<br>90.00<br>103.99<br>90.00	41.81<br>41.07<br>72.24<br>90.00<br>103.86<br>90.00
Wavelength (Å)	0.9184	0.9184	0.9184	0.9184	0.9184	0.9184	0.9184	0.9184
Resolution (Å)	50.00-1.20<br>(1.27-1.20)	50.00-1.20<br>(1.27-1.20)	50.00-0.95<br>(1.01-0.95)	50.00-1.05<br>(1.11-1.05)	41.25-0.99<br>(1.05-0.99)	41.00-1.50<br>(1.59-1.50)	39.55-1.50<br>(1.59-1.50)	41.07-1.35<br>(1.43-1.35)
Number of unique reflections	59,147<br>(3674)	64,053<br>(5044)	145,883<br>(20734)	111,433<br>(17369)	116,159<br>(10502)	37,246<br>(5922)	37,101<br>(5918)	51,336<br>(8146)
Multiplicity	3.7 (3.4)	3.4 (2.4)	3.2 (2.6)	3.3 (3.1)	3.5 (2.5)	3.3 (3.2)	3.8 (3.9)	3.8 (3.8)
Completeness (%)	78.9 (30.6)	85.8 (42.0)	96.0 (84.6)	97.8 (94.6)	87.0 (48.7)	98.8 (98.2)	96.4 (95.9)	97.5 (96.3)
R_{merge}[a]	2.7 (11.9)	5.2 (36.8)	2.9 (46.6)	6.9 (83.5)	3.8 (56.3)	5.9 (77.1)	11.2 (154.2)	8.8 (131.9)
$CC_{(1/2)}$ (%)[b]	99.9 (98.2)	99.8 (82.5)	100.0 (73.8)	99.7 (83.5)	99.9 (74.7)	99.9 (73.5)	99.8 (49.2)	99.8 (52.8)
Average I/s(I)	27.4 (7.9)	12.7 (2.0)	16.2 (1.7)	7.9 (1.1)	17.2 (1.6)	13.4 (1.7)	7.8 (1.0)	9.3 (1.1)
Wilson B (Å^2)	14.5	16.3	12.2	13.9	13.0	25.7	25.7	21.5
**Refinement statistics**								
Resolution range (Å)	40.60-1.20<br>(1.23-1.20)	35.30-1.20<br>(1.23-1.20)	35.64-0.95<br>(0.97-0.95)	40.80-1.05<br>(1.08-1.05)	35.11-0.99<br>(1.02-0.99)	39.21-1.50<br>(1.54-1.50)	39.54-1.50<br>(1.54-1.50)	40.60-1.35<br>(1.38-1.35)
No. of reflections in working set	57716<br>(1356)	60699<br>(1922)	144011<br>(8029)	109331<br>(7353)	114058<br>(3800)	35381<br>(2550)	35991<br>(2638)	48771<br>(3474)
No. of reflections in test set	1248 (51)	3172 (101)	1529 (85)	2101 (141)	2101 (70)	1863 (135)	1114 (82)	2567 (183)
R value (%)[c]	15.5 (18.1)	13.2 (20.4)	13.7 (27.6)	14.1 (37.0)	12.4 (26.3)	16.3 (31.0)	18.6 (38.0)	17.7 (39.0)
R_{free} value (%)[d]	15.9 (18.4)	16.3 (22.4)	14.8 (31.4)	15.7 (38.4)	14.3 (25.4)	19.5 (32.2)	21.2 (37.6)	19.8 (40.7)
RMSD bond length (Å)	0.010	0.012	0.012	0.011	0.012	0.015	0.013	0.013
RMSD angle (°)	1.55	1.63	1.68	1.58	1.66	1.73	1.62	1.65
Number of atoms in AU<br>(protein/ligand/water molecules)	2419<br>21136/18/264	2439<br>2170/19/250	2544<br>2246/20/277	2524<br>2218/19/286	2544<br>2131/42/370	2233<br>2037/20/175	2225<br>2080/22/122	2391<br>2140/21/229
Mean B value (Å^2)	11.9	13.8	13.4	15.5	14.1	21.4	22.3	19.6
**Ramachandran plot statistics**[e]								
Residues in favored regions (%)	96.1	96.5	96.1	96.5	96.5	97.3	96.9	96.9
Residues in allowed regions (%)	3.9	3.5	3.9	3.5	3.5	2.7	3.1	3.1
PDB code	6YZL	6YZJ	6YZN	6Z04	6YZK	6YZM	6YZO	6YZP

The data in parentheses refer to the highest-resolution shell.

[a] R_{merge} = ($|I_{hkl}$ - $\langle I \rangle$)/I_{hkl}, where the average intensity $\langle I \rangle$ is taken over all symmetry equivalent measurements and I_{hkl} is the measured intensity for any given reflection

[b] $CC_{(1/2)}$ is the correlation coefficient between random half data sets and from its value the Pearson correlation coefficient of the true level of signal can be calculated: $CC^* = \sqrt{2CC_{1/2}/1 + CC_{1/2}}$ [2]

[c] R-value = $||F_o| - |F_c||/|F_o|$, where F_o and F_c are the observed and calculated structure factors, respectively

[d] R_{free} is equivalent to R-value but is calculated for 5% of the reflections chosen at random and omitted from the refinement process [3]

[e] As determined by Molprobity [4]

Table S2. Diffraction data collection and refinement statistics of the CA II complexes.

CA II in complex with								
**Ligand**	closo ethyl	closo propyl	closo butyl	nido butyl	closo pentyl	nido pentyl	closo hexyl	nido hexyl
	**2a**	**3a**	**4a**	**4b⁻**	**5a**	**5b⁻**	**6a**	**6b⁻**
**Data collection statistics**								
Space group	$P2_1$	$P2_1$	$P2_1$	$P2_1$	$P2_1$	$P2_1$	$P2_1$	$P2_1$
Cell parameters (Å; °)	42.10<br>41.25<br>72.15<br>90.00<br>104.26<br>90.00	42.16<br>41.46<br>72.35<br>90.00<br>104.54<br>90.00	42.11<br>41.39<br>72.16<br>90.00<br>104.43<br>90.00	42.22<br>41.37<br>71.91<br>90.00<br>104.33<br>90.00	42.05<br>41.30<br>72.02<br>90.00<br>104.31<br>90.00	42.26<br>41.39<br>71.84<br>90.00<br>104.31<br>90.00	42.22<br>41.44<br>72.26<br>90.00<br>104.46<br>90.00	42.15<br>41.34<br>72.35<br>90.00<br>104.44<br>90.00
Wavelength (Å)	0.91841	0.91841	0.91841	0.91841	0.91841	0.91841	0.91841	0.91841
Resolution (Å)	50.00-1.65<br>(1.75-1.65)	50.00-1.05<br>(1.11-1.05)	41.39-1.04<br>(1.11-1.04)	40.91-1.20<br>(1.27-1.20)	41.30-1.05<br>(1.11-1.05)	41.38-1.00<br>(1.06-1.00)	40.89-1.03<br>(1.09-1.03)	35.61-1.09<br>(1.16-1.09)
Number of unique reflections	27514<br>(1863)	106801<br>(18215)	106505<br>(15622)	74406<br>(11929)	106217<br>(16076)	122054<br>(16373)	107589<br>(9486)	93252<br>(12807)
Multiplicity	2.6 (2.6)	12.9 (13.1)	4.2 (4.0)	4.6 (4.6)	4.9 (4.7)	2.5 (1.8)	3.5 (2.3)	3.8 (3.7)
Completeness (%)	97.0 (95.4)	94.5 (91.8)	93.3 (85.0)	98.5 (98.3)	94.7 (89.0)	93.9 (78.1)	89.9 (49.3)	92.4 (79.0)
R_{merge}[a]	11.3 (69.3)	8.2 (154.1)	7.7 (89.2)	5.4 (58.8)	5.6 (100.2)	3.6 (59.6)	5.2 (74.8)	6.0 (108.4)
$CC_{(1/2)}$ (%)[b]	99.9 (69.3)	99.9 (72.2)	99.9 (56.5)	99.9 (81.4)	99.9 (69.8)	99.9 (69.6)	99.9 (59.6)	99.9 (53.0)
Average I/s(I)	8.5 (1.6)	16.43 (1.4)	10.6 (1.7)	15.2 (2.5)	14.4 (1.6)	14.8 (1.6)	12.0 (1.3)	10.9 (1.2)
Wilson B (Å^2)	20.5	15.4	13.5	16.8	14.2	12.9	13.9	15.8
**Refinement statistics**								
Resolution range (Å)	40.80-1.65<br>(1.69-1.65)	40.80-1.05<br>(1.08-1.05)	40.79-1.04<br>(1.07-1.04)	40.90-1.20<br>(1.23-1.19)	40.74-1.05<br>(1.08-1.05)	39.86-1.00<br>(1.03-1.00)	40.88-1.03<br>(1.06-1.03)	35.60-1.10<br>(1.13-1.10)
No. of reflections in working set	26906<br>(1920)	105732<br>(7466)	105334<br>(6357)	73358<br>(5335)	105091<br>(7016)	121249<br>(6331)	105430<br>(2676)	89695<br>(5599)
No. of reflections in test set	1417 (101)	1068 (75)	1118 (67)	1089 (79)	1116 (75)	1287 (67)	2152 (54)	1831 (114)
R value (%)[c]	17.7 (30.1)	17.6 (32.1)	14.1 (33.8)	13.3 (21.1)	13.8 (28.1)	12.6 (26.6)	12.8 (30.3)	14.3 (27.9)
R_{free} value (%)[d]	20.4 (32.8)	20.8 (35.4)	16.0 (32.6)	16.2 (28.1)	17.2 (26.4)	14.0 (24.6)	15.5 (32.6)	16.6 (29.6)
RMSD bond length (Å)	0.018	0.018	0.017	0.016	0.016	0.012	0.012	0.012
RMSD angle (°)	1.86	1.88	1.80	1.82	1.80	1.67	1.63	1.64
Number of atoms in AU<br>(protein/ligand/water molecules)	2377<br>2124/19/234	2435<br>2131/38/262	2487<br>2091/40/355	2486<br>2120/57/308	2537<br>2126/42/367	2608<br>2163/60/384	2520<br>2158/22/339	2396<br>2098/42/255
Mean B value (Å^2)	19.9	19.6	18.6	17.5	17.6	15.2	15.8	17.4
**Ramachandran plot statistics**[e]								
Residues in favored regions (%)	95.7	96.9	96.5	96.9	96.5	96.1	96.9	97.3
Residues in allowed regions (%)	4.3	3.1	3.5	3.1	3.5	3.9	3.1	2.7
PDB code	6YZV	6YZT	6YZQ	6YZR	6YZS	6YZU	6YZW	6YZX

The data in parentheses refer to the highest-resolution shell.

[a] R_{merge} = ($|I_{hkl}$ - $\langle I \rangle$)/I_{hkl}, where the average intensity $\langle I \rangle$ is taken over all symmetry equivalent measurements and I_{hkl} is the measured intensity for any given reflection

[b] $CC_{(1/2)}$ is the correlation coefficient between random half data sets and from its value the Pearson correlation coefficient of the true level of signal can be calculated: $CC^{*} = \sqrt{2CC_{1/2}/1 + CC_{1/2}}$ [2]

[c] R-value = $||F_o| - |F_c||/|F_o|$, where F_o and F_c are the observed and calculated structure factors, respectively

[d] R_{free} is equivalent to R-value but is calculated for 5% of the reflections chosen at random and omitted from the refinement process [3]

[e] As determined by Molprobity [4]

Table S3. List of contacts between compounds and CA IX-mimic.

Residue		**Compound** (compound conformation)										
Number	Amino acid	Interacting part[a]	**1a** (A)	**2a** (A)	**3a** (A)	**3b$^-$** (A)	**4a** (A)	**4b$^-$** (A)	**5a** (A / B)	**5b$^-$** (A)	**6a** (A)	**6b$^-$** (A)
64	H	L	—	—	1	—	—	—	—	—	—	—
64	H	C	—	—	—	—	—	—	—	—	—	—
92	Q	L	—	—	—	—	—	1	—	—	—	1
92	Q	C	8	3	1	3	—	—	—	2	—	—
94	H	L	8	7	8	7	6	6	7 / 6	7	7	7
94	H	C	—	—	—	—	—	—	—	—	—	—
96	H	L	2	2	2	2	2	2	2 / 2	2	2	2
96	H	C	—	—	—	—	—	—	—	—	—	—
119	H	L	1	4	3	3	5	5	4 / 5	5	5	4
119	H	C	—	—	—	—	—	—	—	—	—	—
121	V	L	1	1	1	1	1	1	1 / 2	1	1	3
121	V	C	—	—	—	1	—	—	—	—	—	—
131	V	L	—	—	—	—	—	—	—	—	—	—
131	V	C	—	—	—	2	1	1	—	1	—	1
135	V	L	—	—	—	—	—	—	—	—	—	1
135	V	C	—	—	—	2	2	4	2 / 7	2	3	2
143	V	L	—	1	—	—	1	1	0 / 1	1	1	4
143	V	C	—	—	—	—	—	—	—	—	—	—
198	L	L	5	2	3	4	2	4	2 / 4	4	2	9
198	L	C	4	1	4	—	1	1	1 / 1	2	—	—
199	T	L	3	7	6	6	7	6	5 / 8	8	8	1
199	T	C	—	—	—	—	—	—	—	—	—	—
200	T	L	2	2	2	2	1	1	2 / 0	1	1	1
200	T	C	3	2	—	—	—	—	—	—	—	—
209	W	L	—	1	1	—	2	2	1 / 1	2	2	3
209	W	C	—	—	—	—	—	—	—	—	—	—

[a] Abbreviations representing which part of a compound is responsible for interaction (C = cluster; L = linker + sulfonamide moiety)

Table S4. List of contacts between compounds and CA II.

Residue		**Compound** (compound conformation)								
Number	Amino acid	Interacting part[a]	**2a** (A)	**3a** (A / B)	**4a** (A / B)	**4b$^-$** (A / B / C)	**5a** (A / B)	**5b$^-$** (A)	**6a** (A)	**6b$^-$** (A / B)
64	H	L	—	—	—	—	—	—	—	0 / 1
64	H	C	—	—	—	—	—	—	—	—
67	N	L	—	—	—	—	—	—	—	0 / 4
67	N	C	—	—	—	—	—	—	—	0 / 2
91	I	L	—	—	—	—	—	—	—	—
91	I	C	—	—	—	0 / 1 / 0	—	—	—	—
92	Q	L	—	—	—	0 / 0 / 1	—	—	—	2 / 4
92	Q	C	3	1 / 2	4 / 1	0 / 4 / 0	—	—	—	—
94	H	L	7	8 / 6	8 / 7	7 / 7 / 6	6 / 7	7	7	7 / 11
94	H	C	—	—	—	—	—	—	—	—
96	H	L	2	2 / 2	2 / 2	2 / 2 / 2	2 / 2	2	2	2 / 2
96	H	C	—	—	—	—	—	—	—	—
119	H	L	3	3 / 1	2 / 4	4 / 5 / 3	5 / 5	5	5	6 / 6
119	H	C	—	—	—	—	—	—	—	—
121	V	L	1	1 / 0	1 / 1	1 / 4 / 1	1 / 2	3	2	2 / 2
121	V	C	—	1 / 3	3 / 2	0 / 2 / 0	—	—	—	—
131	F	L	—	—	—	—	1 / 0	1	2	3 / 0
131	F	C	3	4 / 5	6 / 6	2 / 20 / 0	6 / 6	5	3	4 / 1
135	V	L	—	—	—	—	—	—	—	—
135	V	C	—	—	1 / 1	0 / 2 / 1	—	—	—	—
141	L	L	—	—	—	—	—	—	—	—
141	L	C	—	0 / 1	1 / 1	—	—	—	—	—
143	V	L	—	—	—	1 / 1 / 0	1 / 1	1	1	1 / 1
143	V	C	—	—	—	—	—	—	—	—
198	L	L	3	4 / 5	3 / 3	6 / 4 / 3	2 / 2	3	2	6 / 3
198	L	C	—	—	—	—	1 / 1	2	—	—
199	T	L	8	5 / 5	7 / 6	7 / 5 / 9	7 / 7	5	6	8 / 7
199	T	C	—	—	—	—	—	—	—	—
200	T	L	1	2 / 2	2 / 2	2 / 1 / 0	2 / 1	—	—	—
200	T	C	3	3 / 0	—	—	—	—	—	—
201	P	L	—	—	—	—	—	—	—	—
201	P	C	—	—	—	—	1 / 1	—	4	—
202	P	L	—	—	—	—	—	—	—	—
202	P	C	—	—	—	1 / 0 / 0	5 / 4	4	—	5 / 0
209	W	L	1	1 / 0	1 / 1	1 / 3 / 3	1 / 1	1	2	2 / 2
209	W	C	—	—	—	—	—	—	—	—

[a] Abbreviations representing which part of a compound is responsible for interaction (C = cluster; L = linker + sulfonamide moiety)

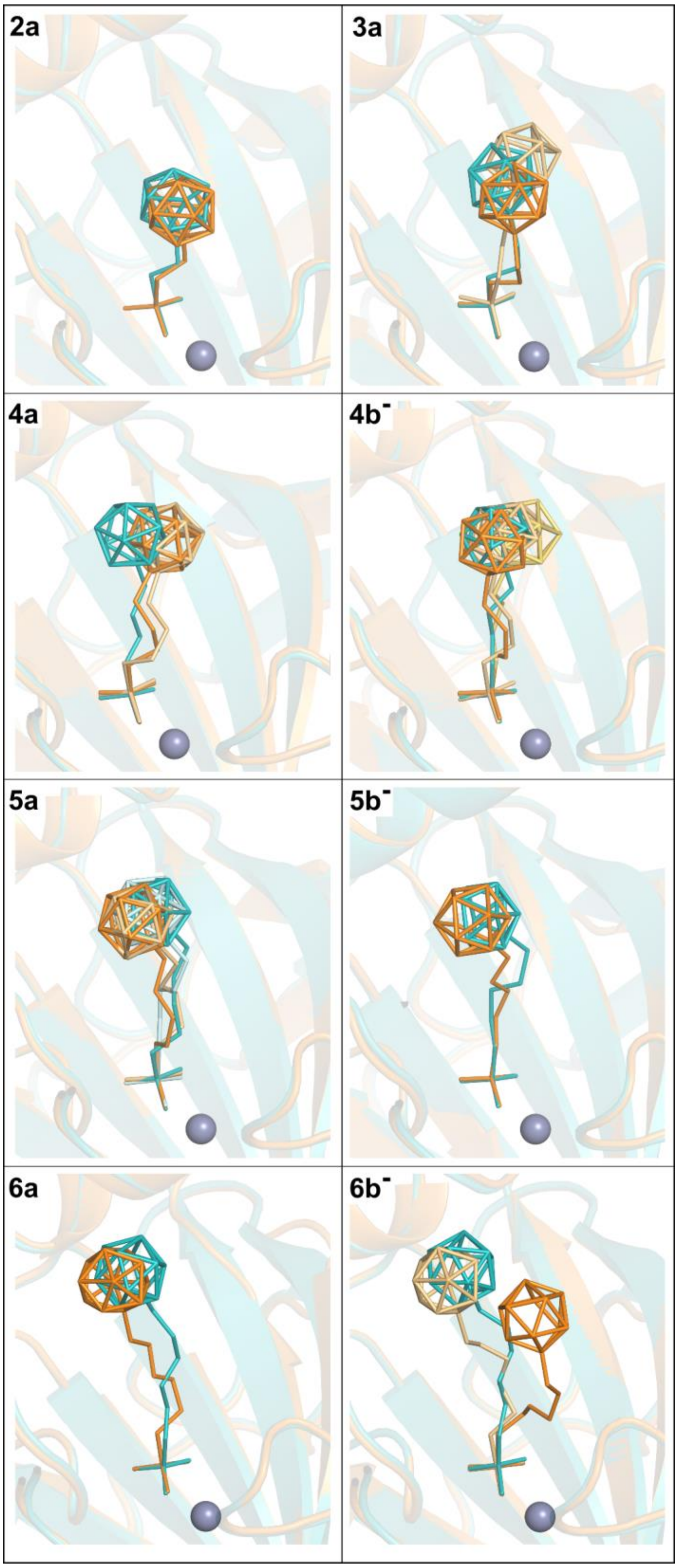

Figure S1. Superposition of compounds in CA IX-mimic and CA II active sites. The structures of compounds are depicted as sticks and protein is represented as cartoon. CA IX-mimic structure is coloured in teal with alternative binding mode of the compounds in pale cyan. CA II structure is coloured in orange with alternative binding modes of the compounds in light and yellow orange, respectively. The zinc ion is shown as the grey sphere.

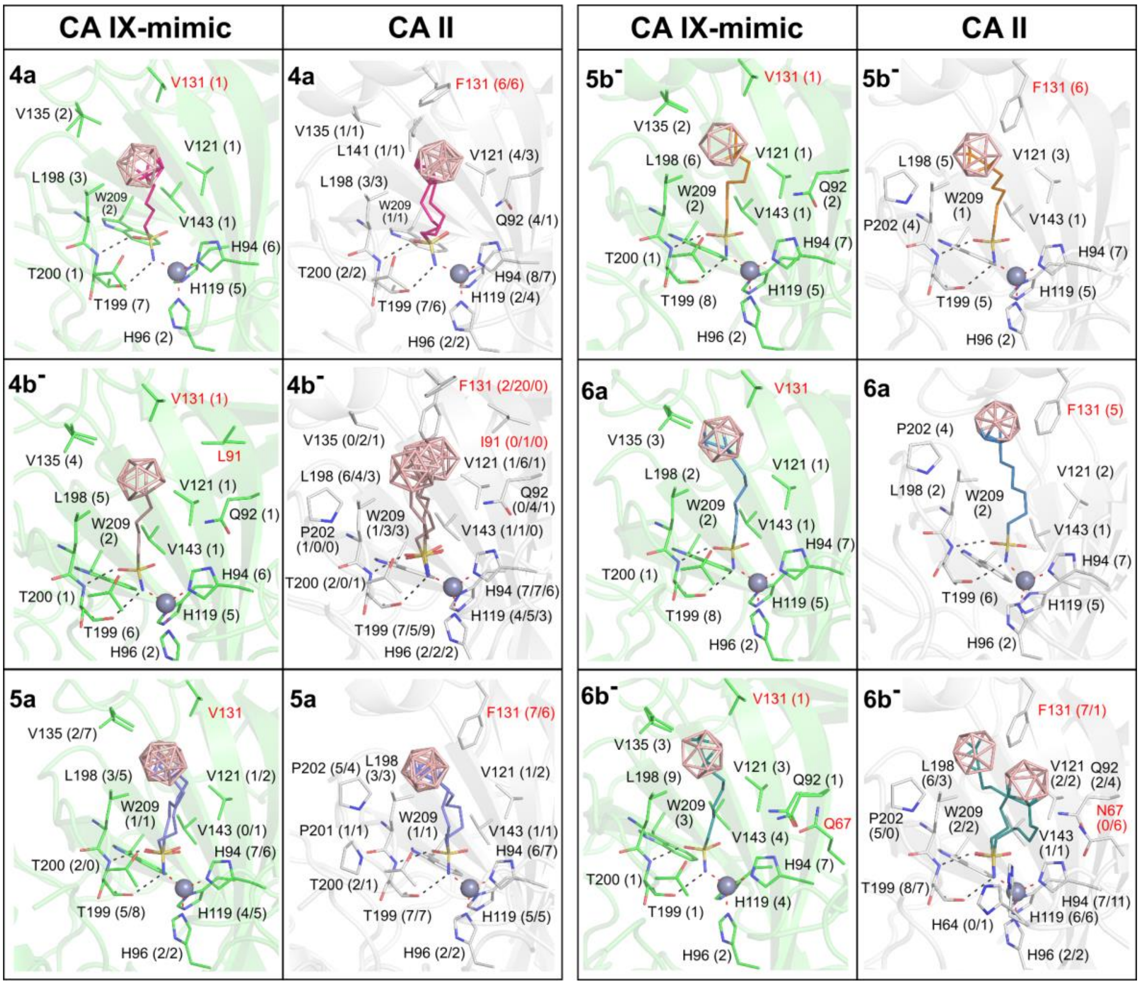

Figure S2. The different binding positions of compounds between the CA IX-mimic and CA II active sites. The structures of compounds are depicted as sticks with differently coloured carbon atoms: **4a** (hot pink), **4b**$^{-}$ (brown), **5a** (slate), **5b**$^{-}$ (orange), **6a** (marine), and **6b**$^{-}$ (deep teal). Boron atoms are coloured pink; oxygen, sulphur, and nitrogen are shown in red, yellow, and blue, respectively. Carbon atoms in CA IX-mimic and CA II are shown in green and white, respectively. Protein is represented as cartoon with the residues interacting with compounds highlighted as sticks. The zinc ion is shown as the grey sphere. Polar contacts between protein and compound are labelled as black dashed lines, and coordination bonds are marked as red dashed lines. The numbers in parentheses give the number of contacts with a distance between the ligand and protein atoms less than or equal to 4 Å. The numbers are separated by slash to express number of contacts for each alternative conformation. The active site residues that vary between CA IX-mimic and CA II are highlighted in red.

Supplementary references

1. Chen J, Wang J, Bai Y, Li K, Garcia ES, Ferguson AL, Zimmerman SC. Enzyme-like click catalysis by a copper-containing single-chain nanoparticle. Journal of the American Chemical Society 2018;140(42):13695–13702.
2. Karplus PA, Diederichs K. Linking crystallographic model and data quality. Science 2012;336(6084):1030–1033.
3. Brünger AT. Free R value: A novel statistical quantity for assessing the accuracy of crystal structures. Nature 1992;355(6359):472–475.
4. Chen VB, Arendall WB, 3rd, Headd JJ, Keedy DA, Immormino RM, Kapral GJ, Murray LW, Richardson JS, Richardson DC. Molprobity: All-atom structure validation for macromolecular crystallography. Acta Crystallogr D Biol Crystallogr 2010;66(Pt 1):12–21.

NMR spectra of new compounds 5a, $5b^-$, 6a and $6b^-$

The spectra for each compound are given in the following order: ^{11}B, $^{11}B\{^{1}H\}$, ^{11}B-^{11}B COSY, $^{1}H\{^{11}B\}$, ^{1}H, $^{1}H\{^{11}B_{selective}\}$ and $^{13}C\{^{1}H\}$ NMR.

The headings in the left corner of each spectrum correspond to the formula of the particular compound.

1-SO2NH2-(CH2)5-OK

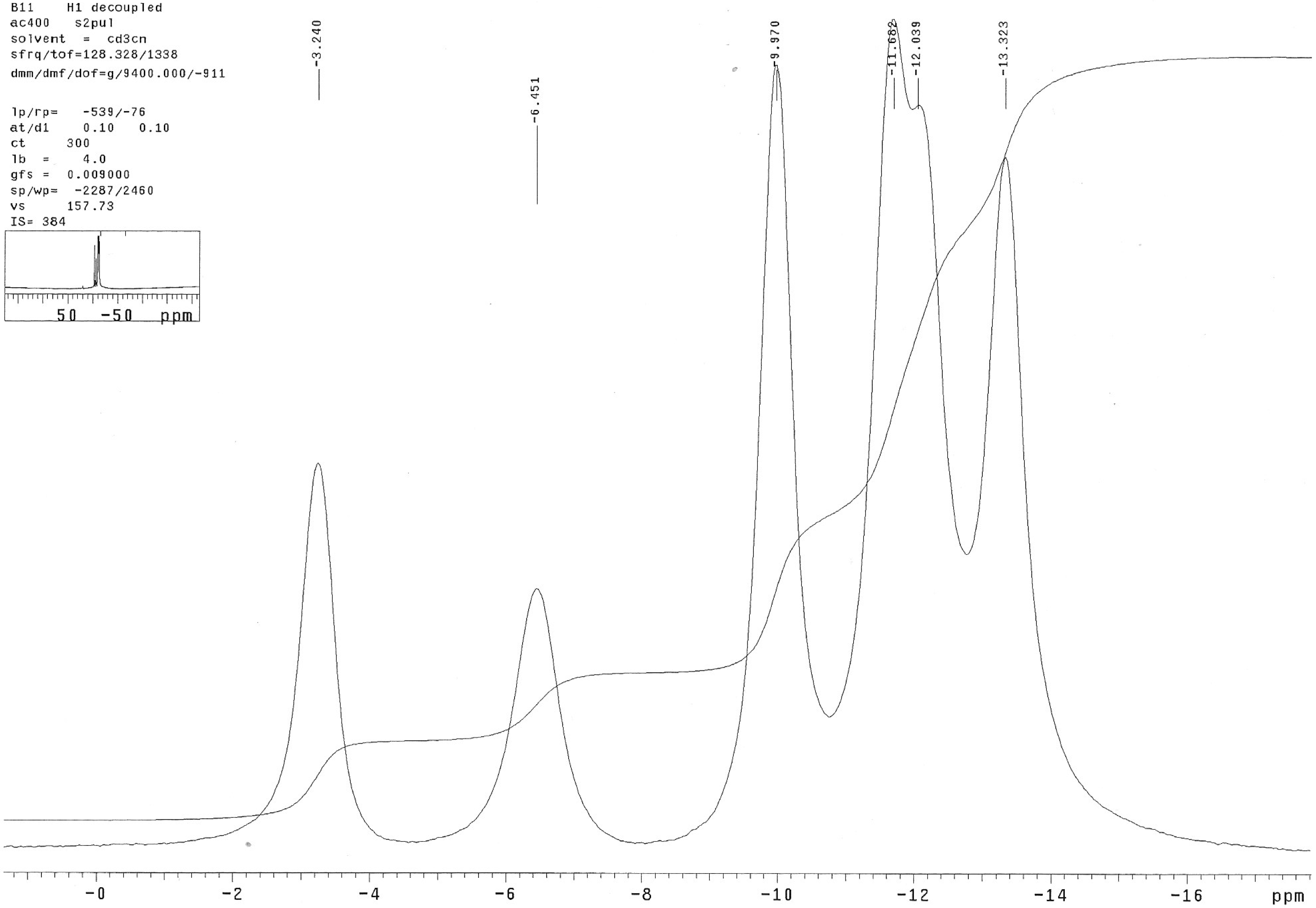

1-SO2NH2-(CH2)5-OK

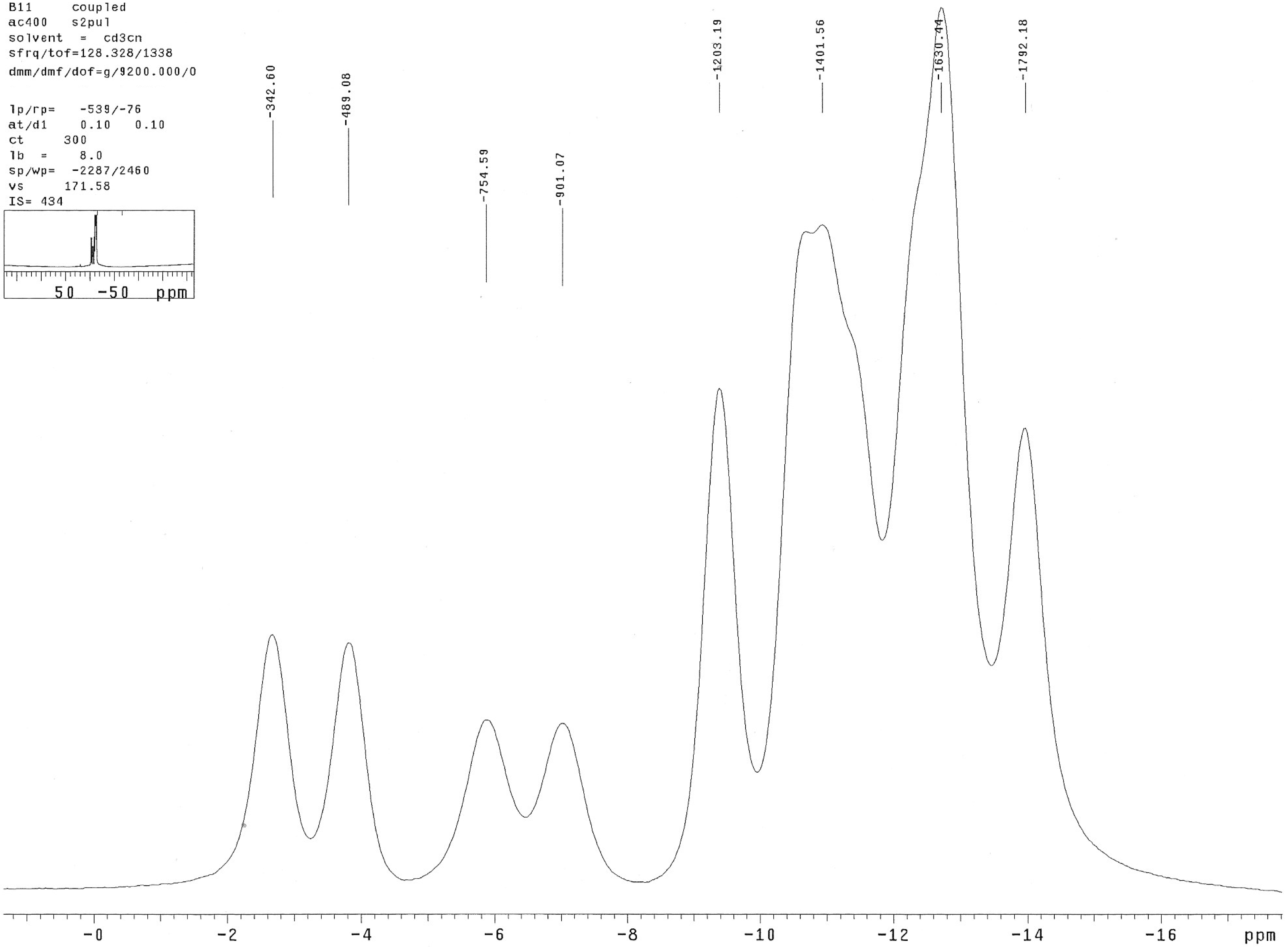

1-SO2NH2-(CH2)5-OK

B11 H1 decoupled
ac400 COSY
solvent = cd3cn
sfrq/tof=128.330/2822
dmm/dmf/dof=g/9600.000/0

lp/rp= -1000/80
at/d1 0.06 0.02
ct 32
lb = 17.1
gf = 0.004000
gfs = 0.008000
sp/wp= -2071/2035
sp1/wp1= -2071/2035
vs 1421.41
IS= 281473

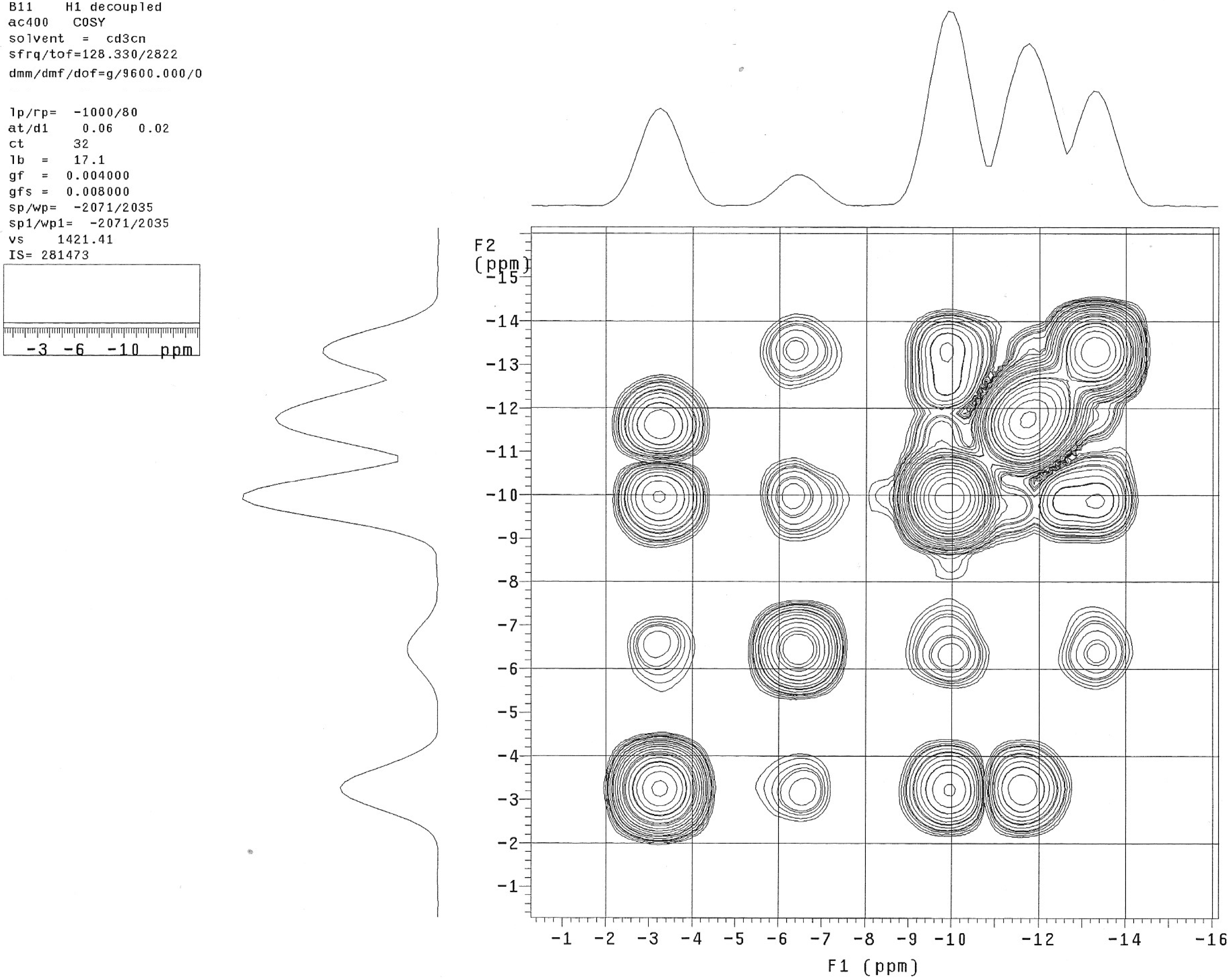

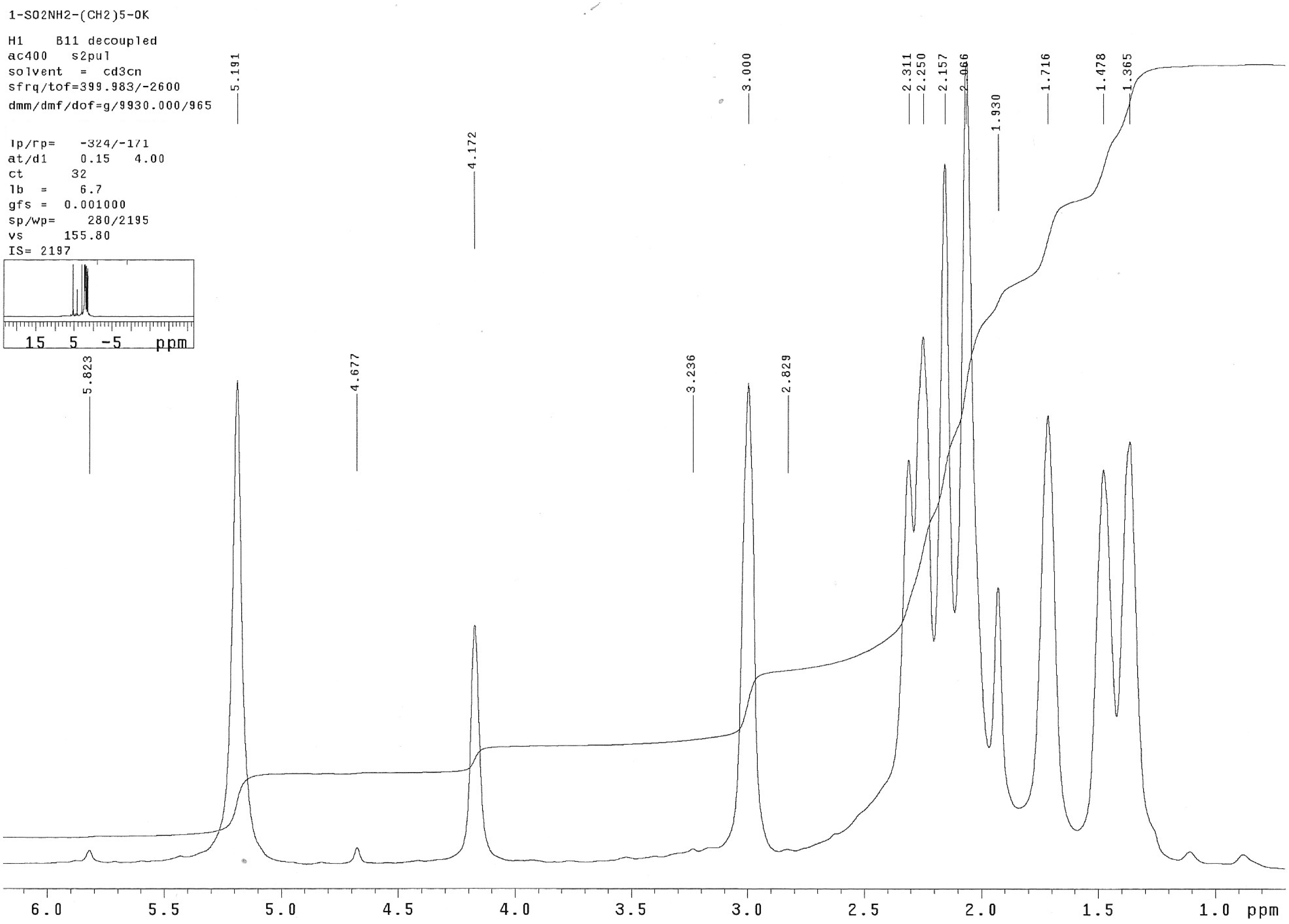
1-SO2NH2-(CH2)5-OK
H1 B11 decoupled
ac400 s2pul
solvent = cd3cn
sfrq/tof=399.983/-2600
dmm/dmf/dof=g/9930.000/965
lp/rp= -324/-171
at/d1 0.15 4.00
ct 32
lb = 6.7
gfs = 0.001000
sp/wp= 280/2195
vs 155.80
IS= 2197
15 5 -5 ppm
5.191
4.172
3.000
2.311
2.250
2.157
2.066
1.930
1.716
1.478
1.365
5.823
4.677
3.236
2.829
6.0
5.5
5.0
4.5
4.0
3.5
3.0
2.5
2.0
1.5
1.0 ppm

1-SO2NH2-(CH2)5-OK

H1 coupled
ac400 s2pul
solvent = cd3cn
sfrq/tof=399.983/-2600
dmm/dmf/dof=c/200.000/0

lp/rp= -324/-175
at/d1 2.00 1.00
ct 16
gf = 0.090000
gfs = 0.001000
sp/wp= 280/2195
vs 168.90
IS= 2456

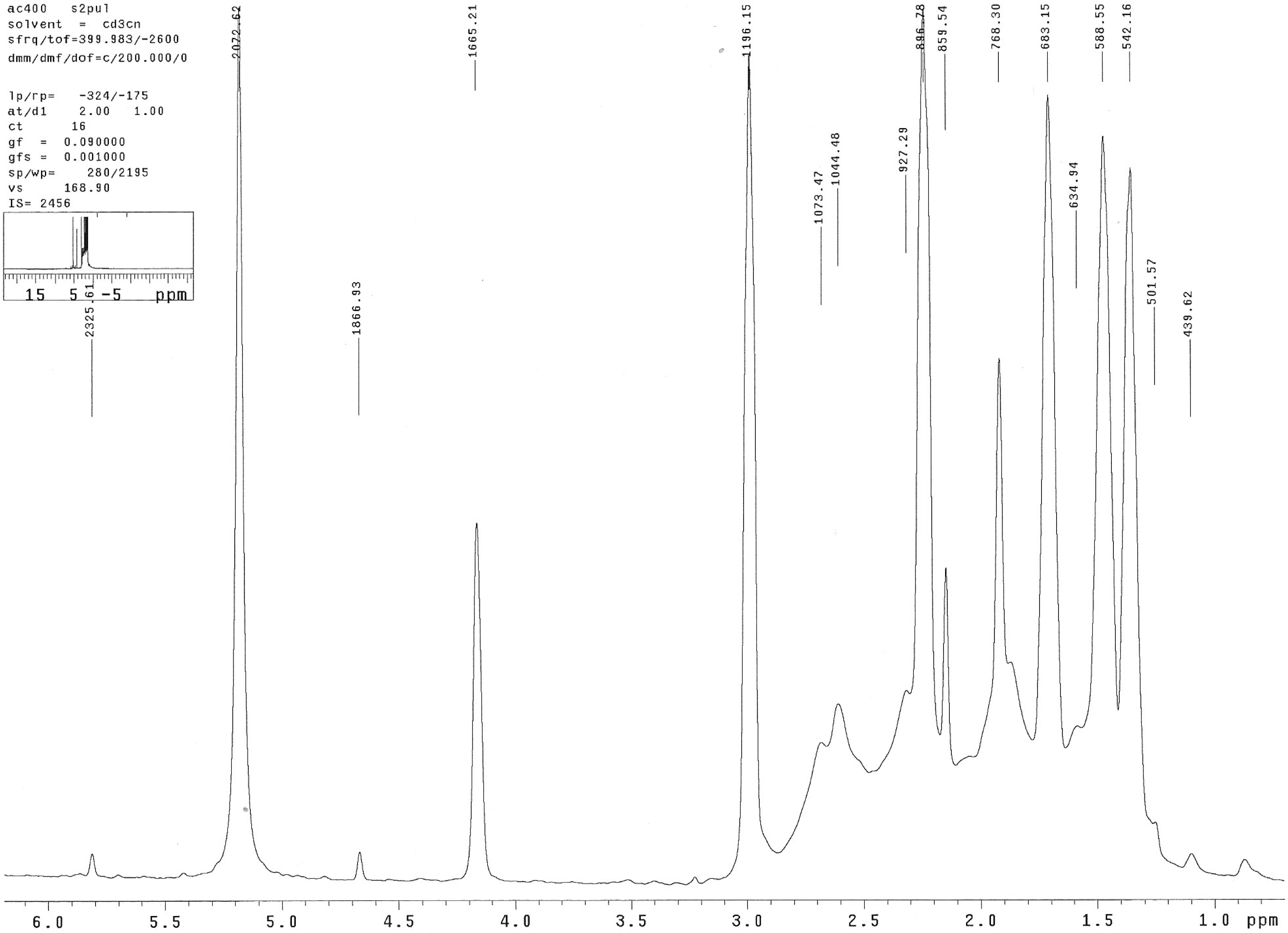

1-SO2NH2-(CH2)5-OK

H1 B11 decoupled
ac400 s2pul
solvent = cd3cn
sfrq/tof=399.985/0
dmm/dmf/dof=c/200.000/-50000

lp/rp= -377/-130
at/d1 0.20 4.00
ct 16
lb = 2.0
gfs = 0.000000
sp/wp= 457/703
vs 521.52
selective according to B11 ppm:
6 -13.329
5 -12.044
4 -11.687
3 -9.974
2 -6.453
1 -3.242
COUPLED SPECTRUM SUBSTRACTED
IS= 3346

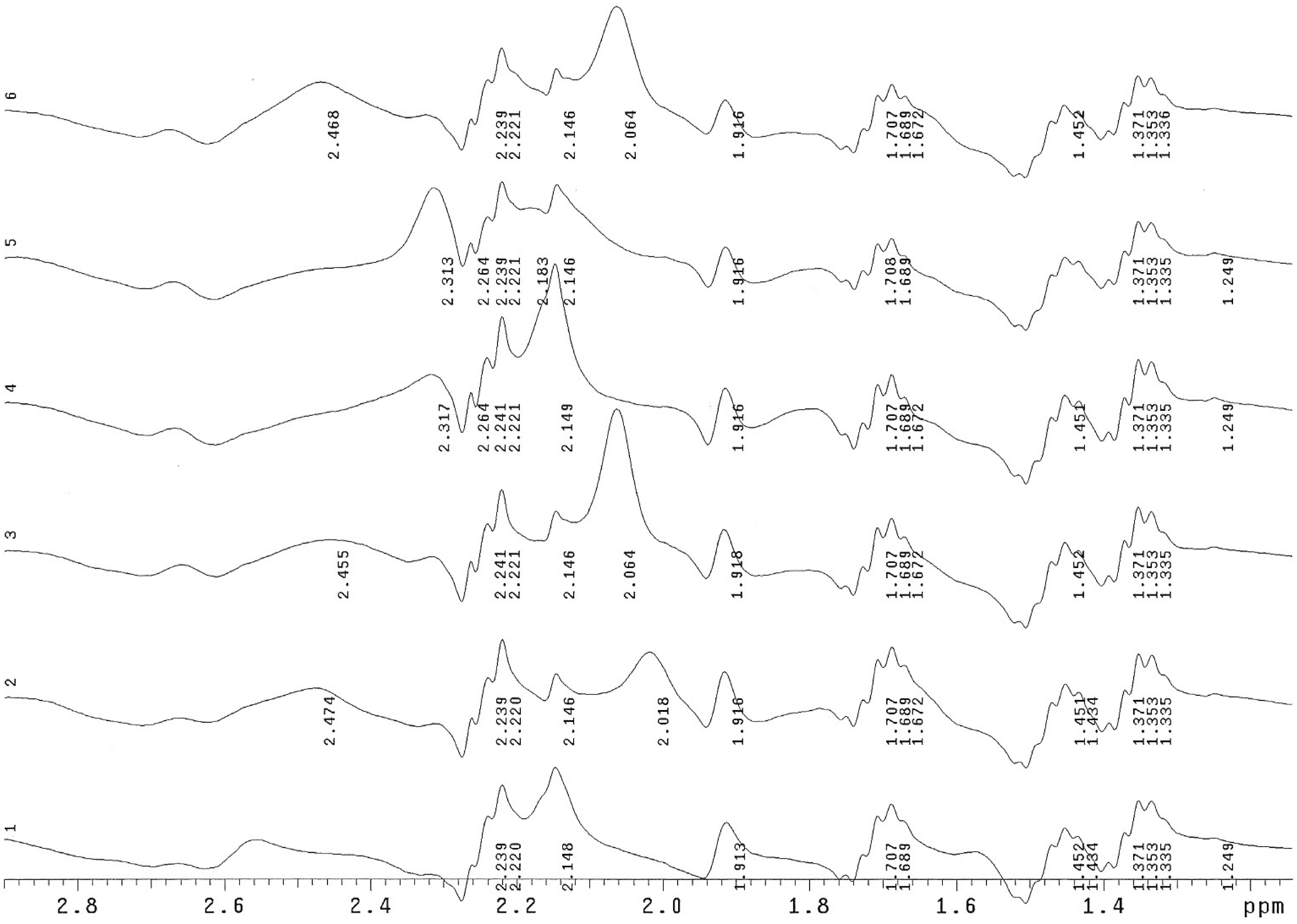

1-SO2NH2-(CH2)5-OK

C13 H1 decoupled
ac400 s2pul
solvent = cd3cn
sfrq/tof=100.585/0
dmm/dmf/dof=g/9400.000/-700

lp/rp= -563/-54
at/d1 1.20 0.20
ct 64
lb = 2.0
sp/wp= 1569 6792
vs 1743.77
IS= 104533

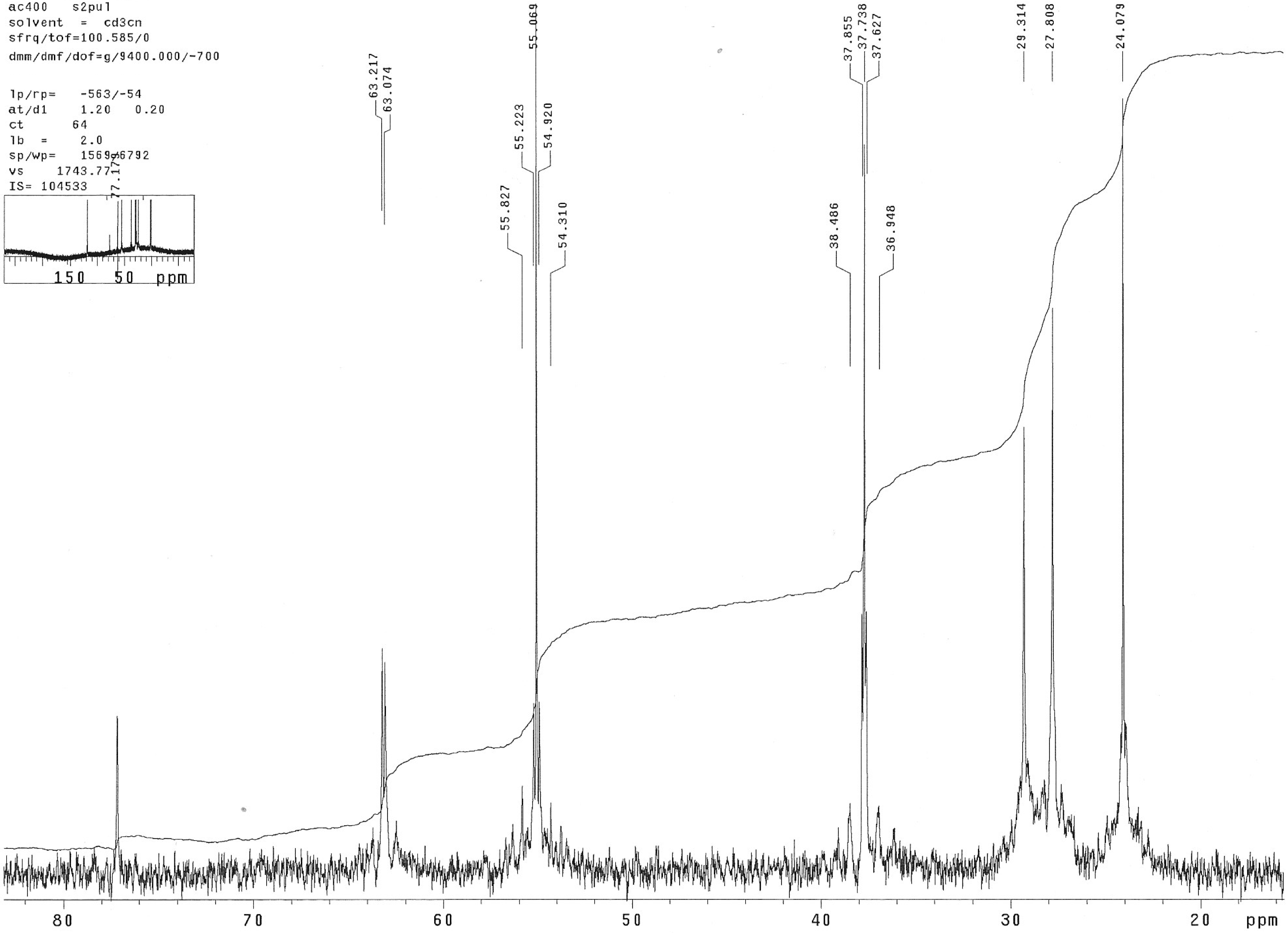

1-SO2NH2-(CH2)6-OK

B11 H1 decoupled
ac400 s2pul
solvent = cd3cn
sfrq/tof=128.328/1338
dmm/dmf/dof=g/9400.000/-911

date Jan 31 2018
lp/rp= -540/-98
at/d1 0.10 0.10
ct 300
lb = 8.0
gfs = 0.009000
sp/wp= -3675/5072
vs 467.80
IS= 572

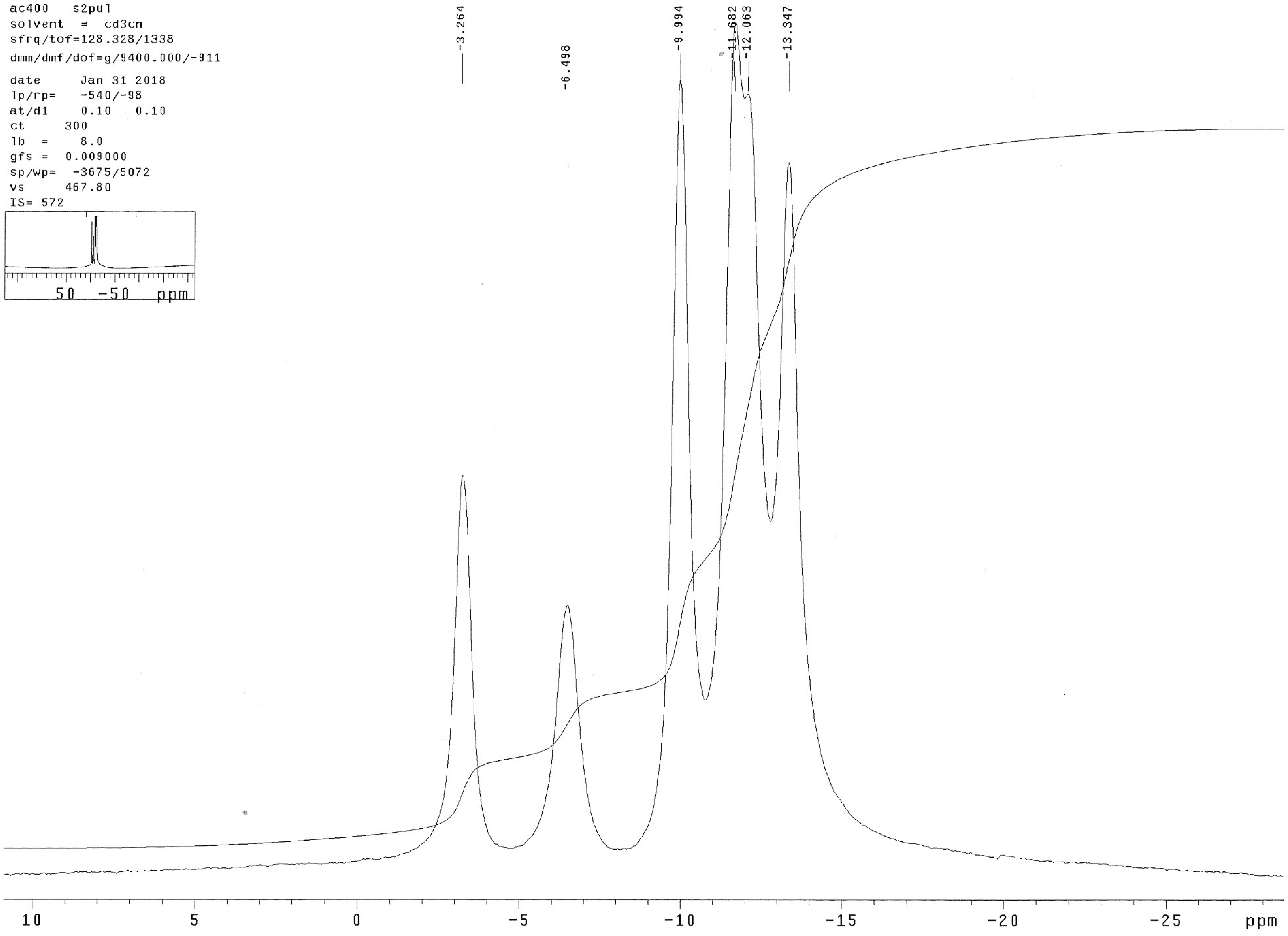

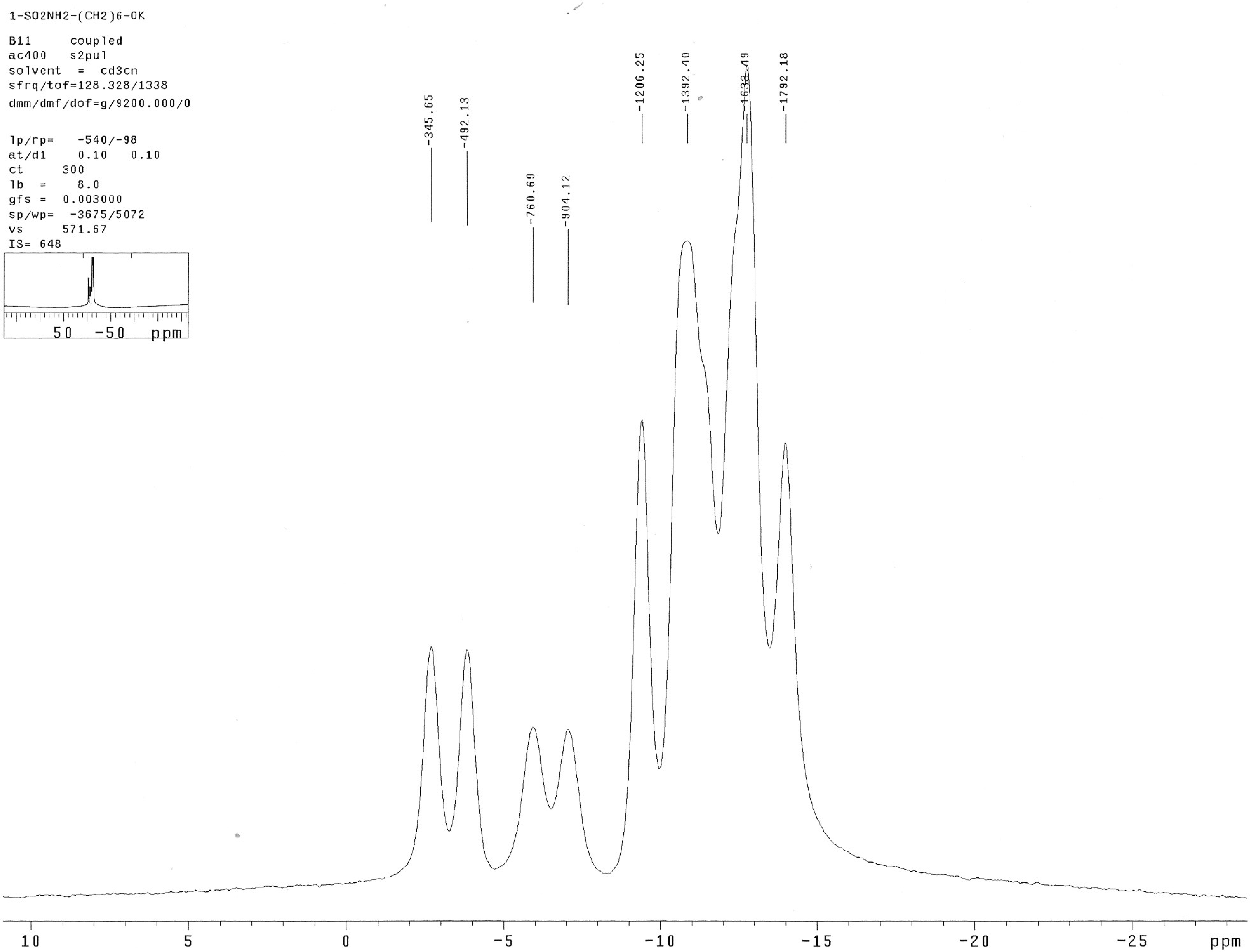
1-SO2NH2-(CH2)6-OK
B11 coupled
ac400 s2pul
solvent = cd3cn
sfrq/tof=128.328/1338
dmm/dmf/dof=g/9200.000/0
lp/rp= -540/-98
at/d1 0.10 0.10
ct 300
lb = 8.0
gfs = 0.003000
sp/wp= -3675/5072
vs 571.67
IS= 648
-345.65
-492.13
-760.69
-904.12
-1206.25
-1392.40
-1633.49
-1792.18
50 -50 ppm
10 5 0 -5 -10 -15 -20 -25 ppm

1-SO2NH2-(CH2)6-OK

B11 H1 decoupled
ac400 COSY
solvent = cd3cn
sfrq/tof=128.330/2821
dmm/dmf/dof=g/9600.000/0
[illegible]

lp/rp= -1000/80
at/d1 0.06 0.02
ct 72
lb = 17.1
gf = 0.004000
gfs = 0.008000
sp/wp= -2116/2121
sp1/wp1= -2116/2121
vs 1421.41
IS= 296159

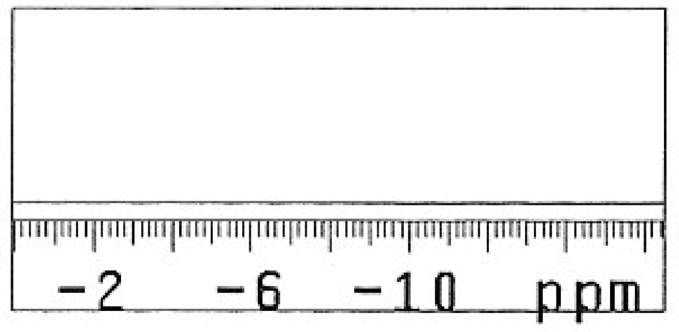

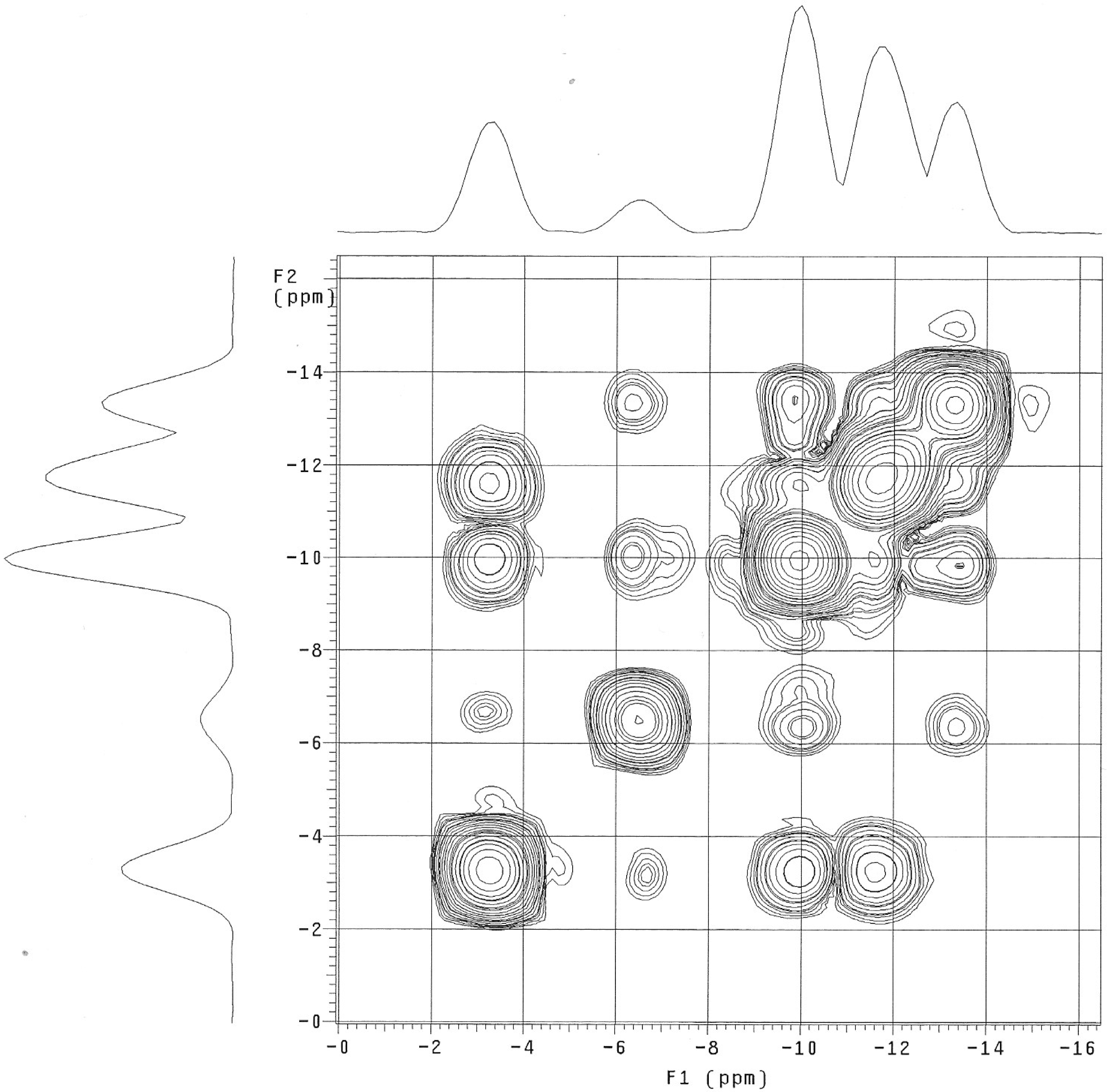

1-SO2NH2-(CH2)6-OK

H1 B11 decoupled
ac400 s2pul
solvent = cd3cn
sfrq/tof=399.983/-2600
dmm/dmf/dof=g/9930.000/965

lp/rp= -381/-80
at/d1 0.15 4.00
ct 16
lb = 6.7
gfs = 0.001000
sp/wp= 404/1842
vs 2183.14
IS= 3104

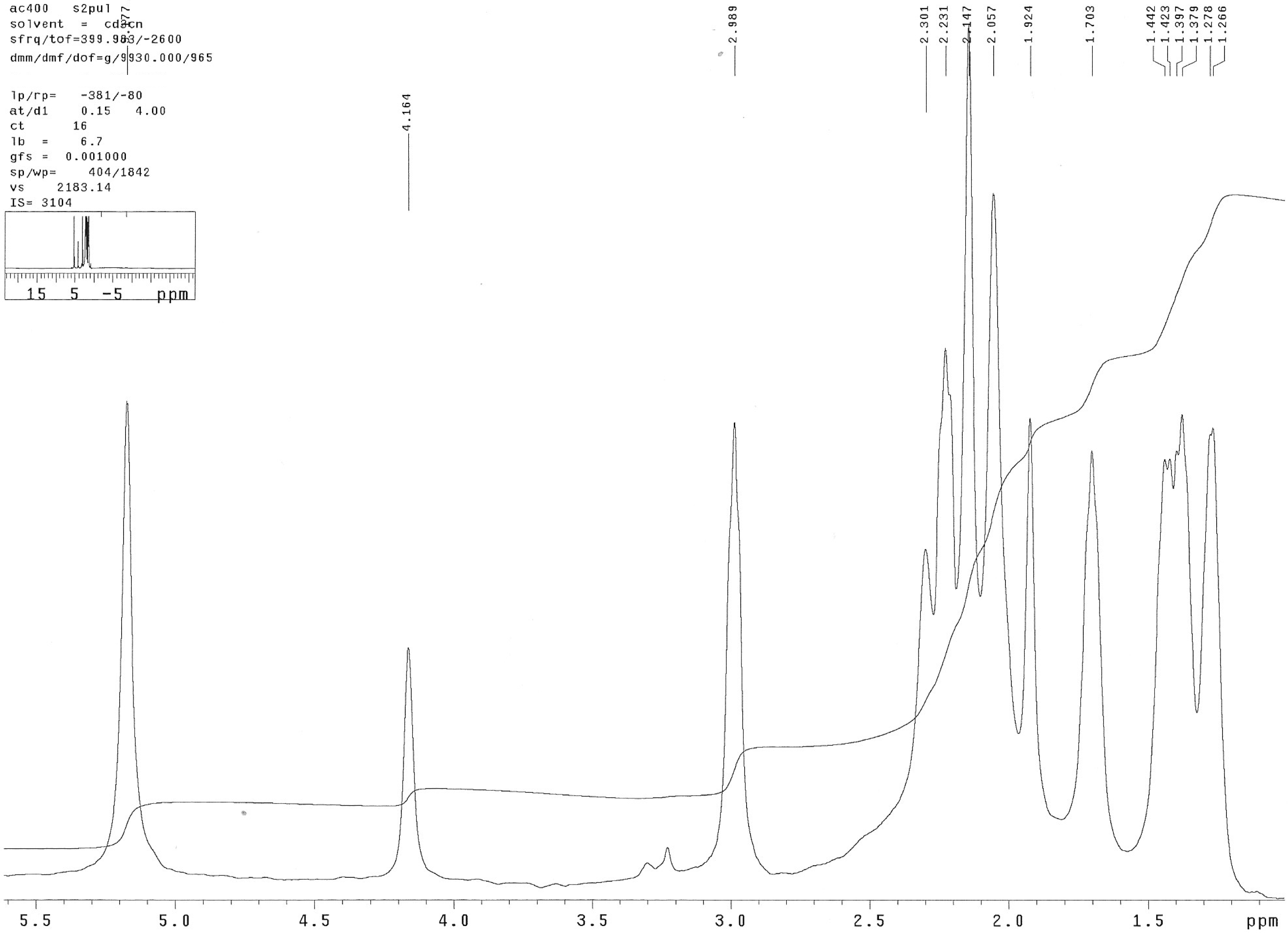

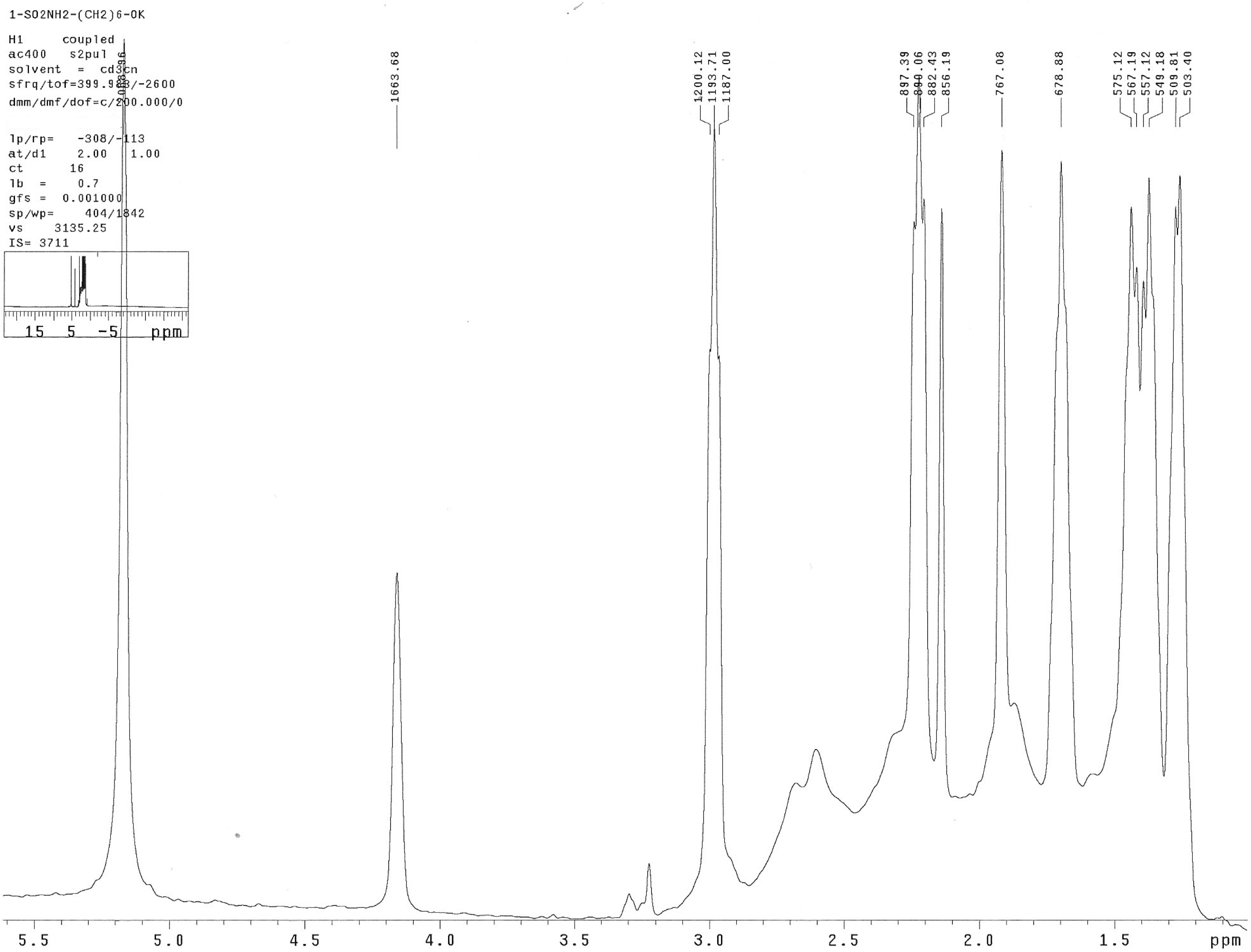

1-SO2NH2-(CH2)6-OK
H1 coupled
ac400 s2pul
solvent = cd3cn
sfrq/tof=399.9[illegible]/-2600
dmm/dmf/dof=c/200.000/0
lp/rp= -308/-113
at/d1 2.00 1.00
ct 16
lb = 0.7
gfs = 0.001000
sp/wp= 404/1842
vs 3135.25
IS= 3711
15 5 -5 ppm
[illegible]
1663.68
1200.12
1193.71
1187.00
897.39
890.06
882.43
856.19
767.08
678.88
575.12
567.19
557.12
549.18
509.81
503.40
5.5
5.0
4.5
4.0
3.5
3.0
2.5
2.0
1.5
ppm

1-SO2NH2-(CH2)6-OK

H1 B11 decoupled
ac400 s2pul
solvent = cd3cn
sfrq/tof=399.985/0
dmm/dmf/dof=c/200.000/-50000

lp/rp= -88/163
at/d1 0.20 4.00
ct 0
lb = 2.0
gfs = 0.000000
sp/wp= 431/690
vs 466.29
selective according to B11 ppm:
6 -13.353
5 -12.068
4 -11.687
3 -9.998
2 -6.525
1 -3.289
COUPLED SPECTRUM SUBSTRACTED
IS= 5048

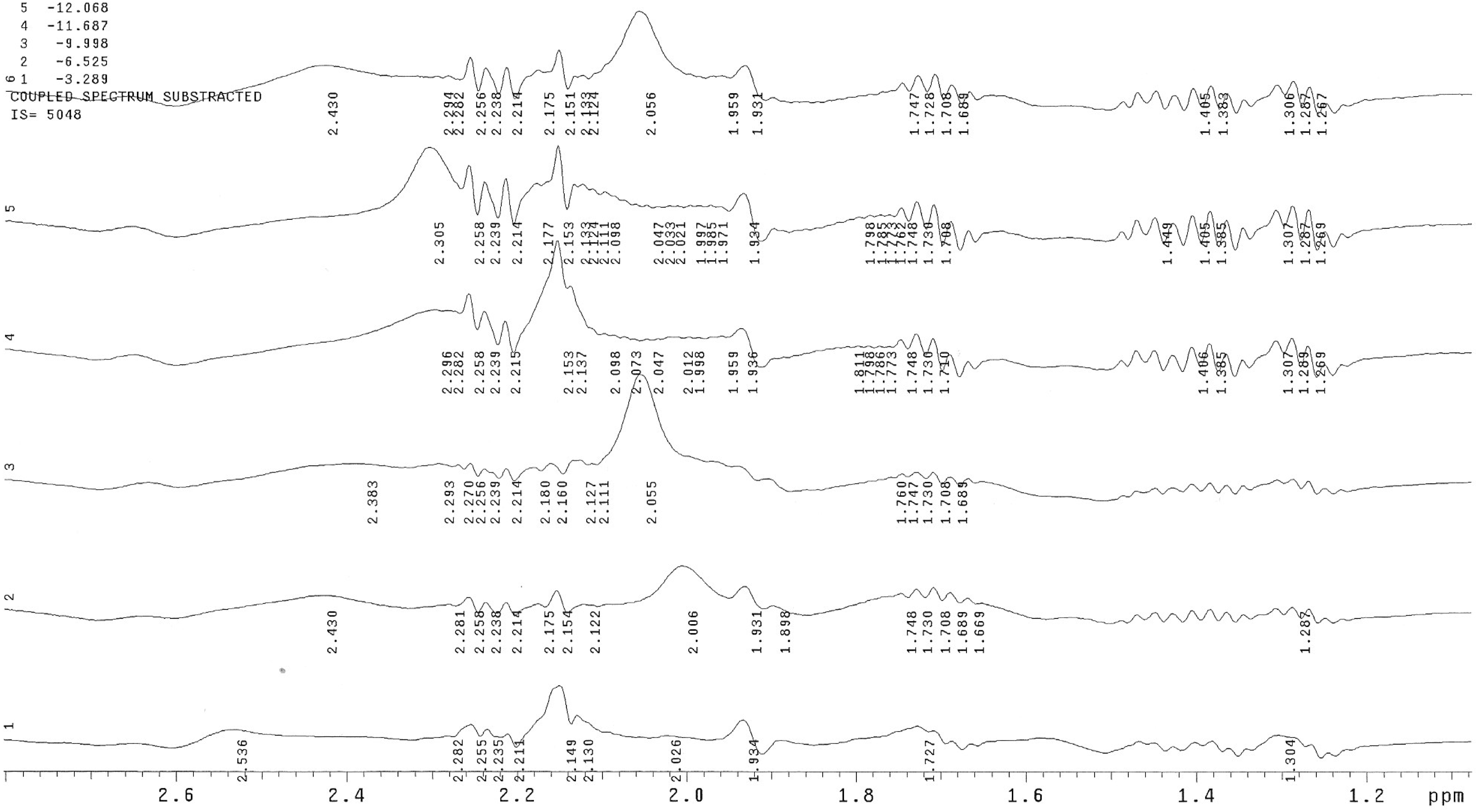

1-SO2NH2-(CH2)6-OK

C13 H1 decoupled
ac400 s2pul
solvent = cd3cn
sfrq/tof=100.585/0
dmm/dmf/dof=g/9400.000/-700

lp/rp= -563/-60
at/d1 1.20 0.20
ct 5184
lb = 2.0
sp/wp= 1550/6543
vs 16286.32
IS= 253771

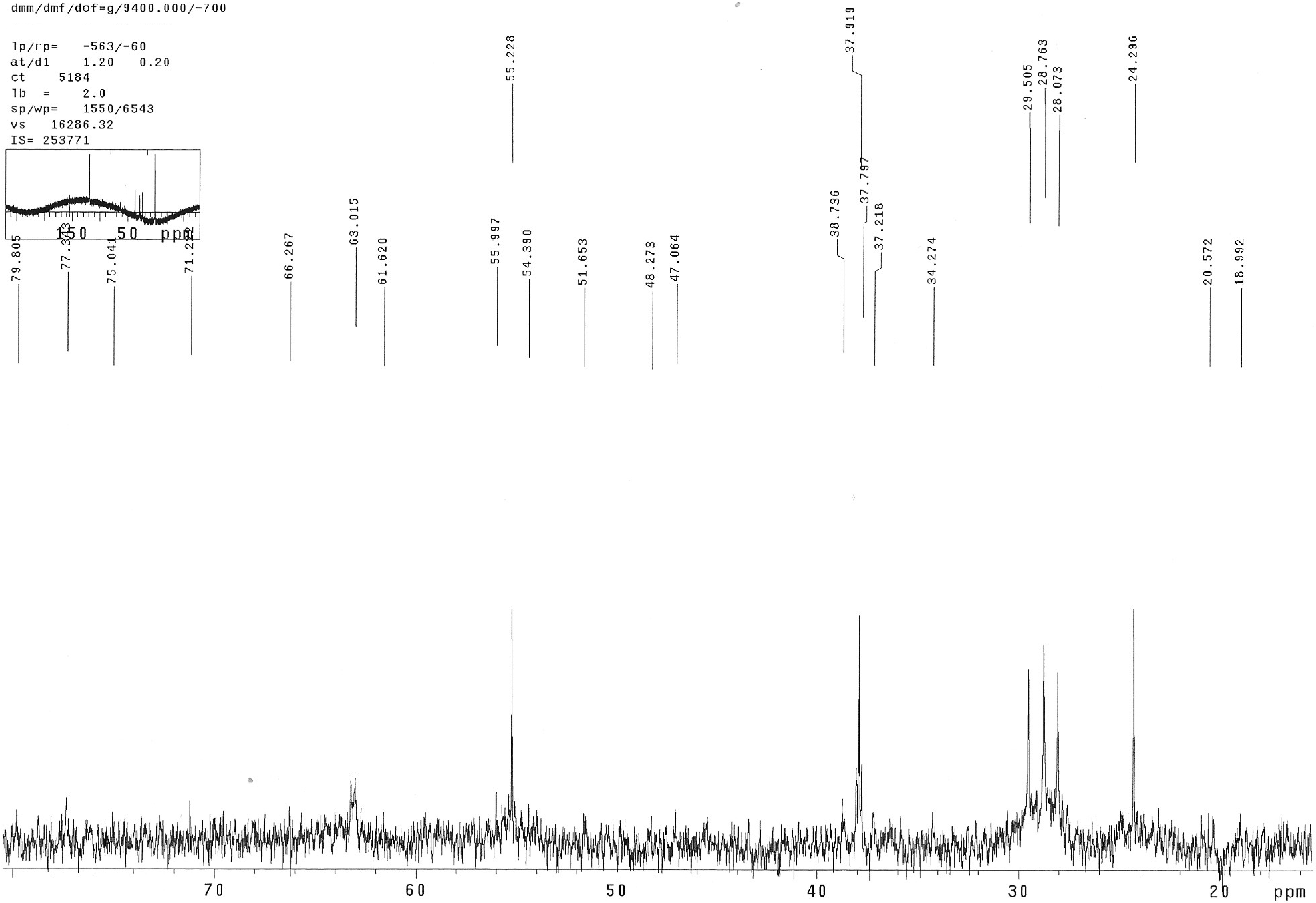

7-SO2NH2-(CH2)5-7,8-C2B9H11(-)K(+)

B11   H1 decoupled
ac400   s2pul
solvent = cd3cn
sfrq/tof=128.328/1338
dmm/dmf/dof=g/9400.000/-911

lp/rp=   -851/117
at/d1   0.10   0.10
ct   300
lb =   4.0
gfs = 0.009000
sp/wp= -5421/5426
vs   98.92
IS= 163

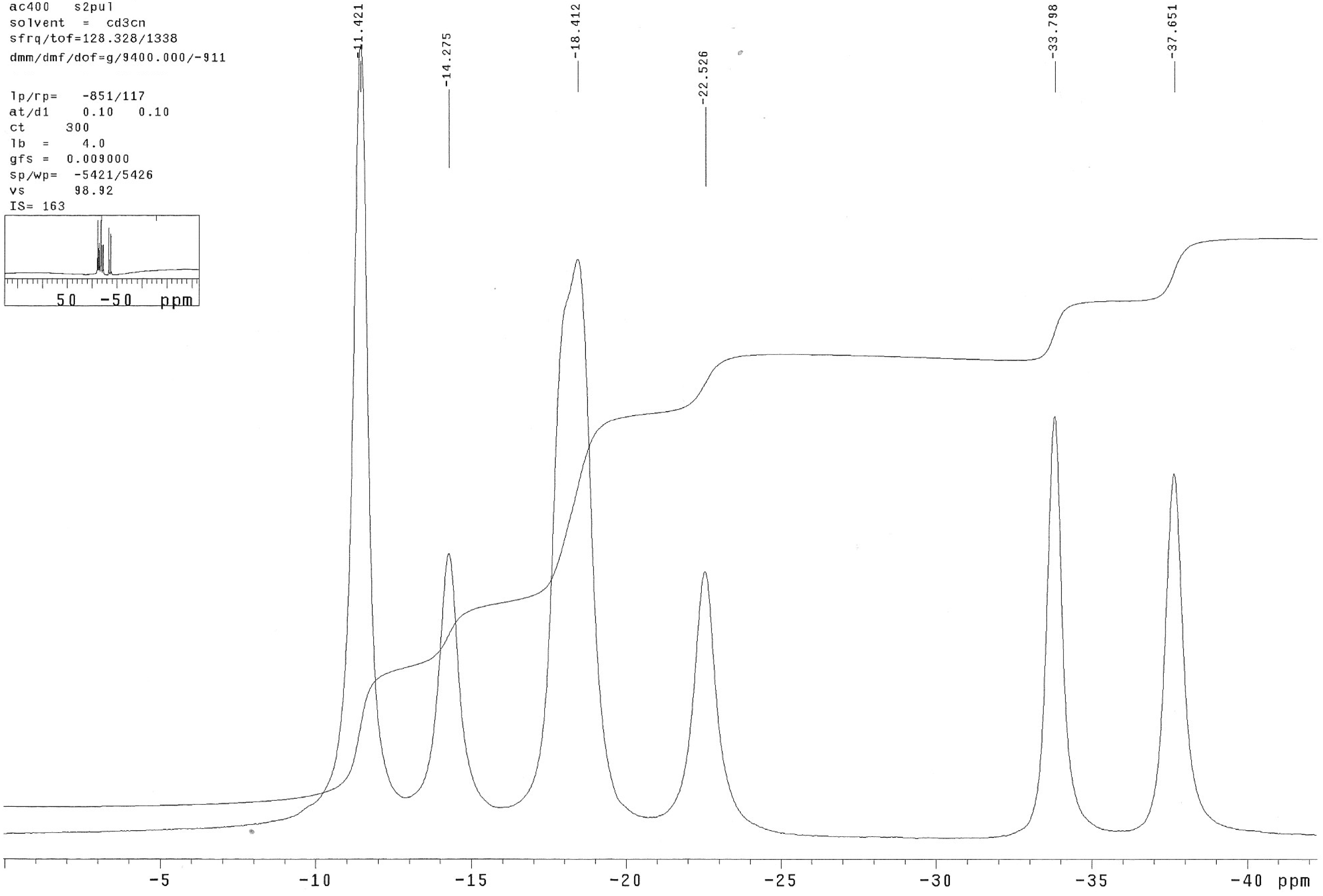

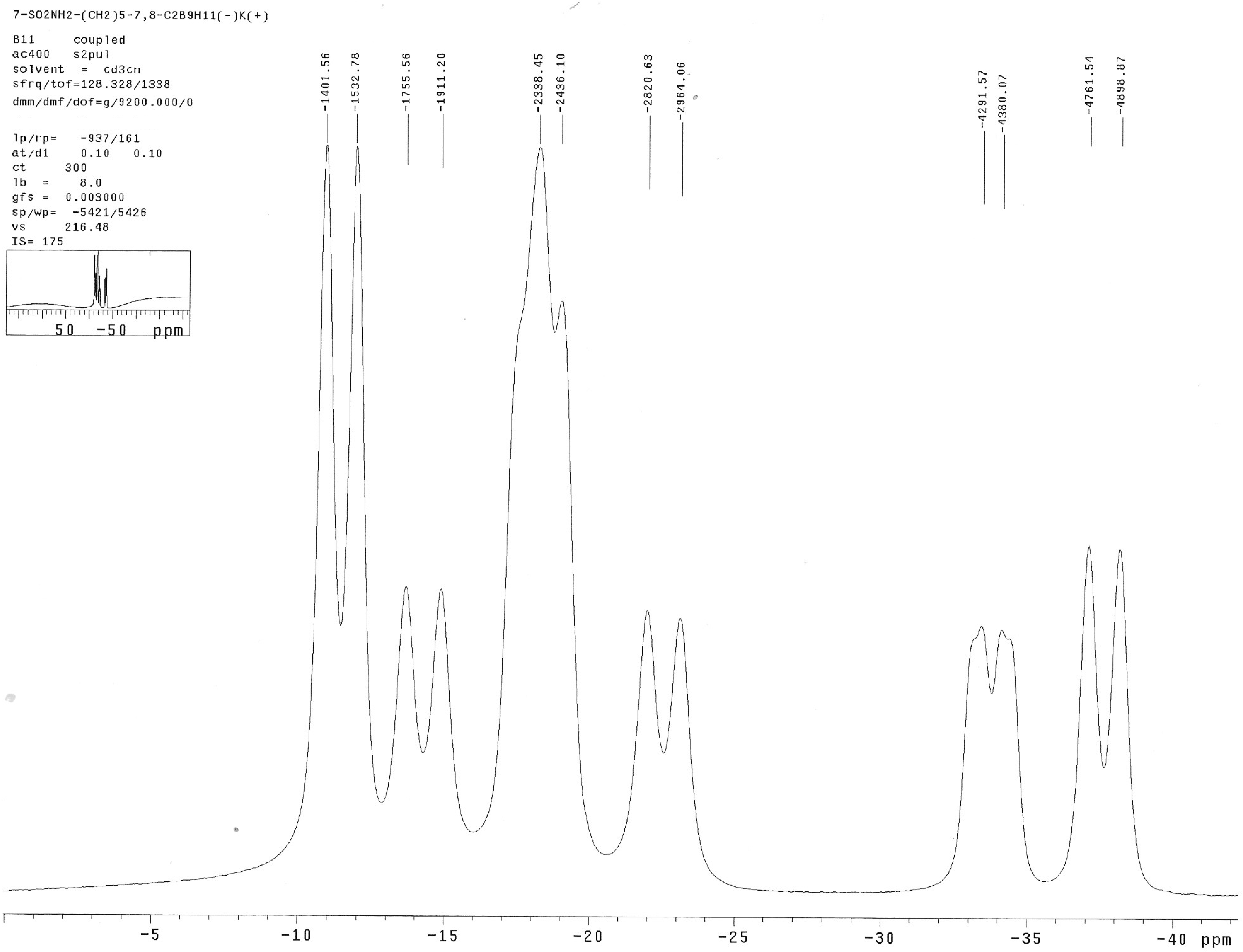
7-SO2NH2-(CH2)5-7,8-C2B9H11(-)K(+)
B11 coupled
ac400 s2pul
solvent = cd3cn
sfrq/tof=128.328/1338
dmm/dmf/dof=g/9200.000/0
lp/rp= -937/161
at/d1 0.10 0.10
ct 300
lb = 8.0
gfs = 0.003000
sp/wp= -5421/5426
vs 216.48
IS= 175
50 -50 ppm
-1401.56
-1532.78
-1755.56
-1911.20
-2338.45
-2436.10
-2820.63
-2964.06
-4291.57
-4380.07
-4761.54
-4898.87
-5
-10
-15
-20
-25
-30
-35
-40 ppm

7-SO2NH2-(CH2)5-7,8-C2B9H11(-)K(+)

B11 H1 decoupled
ac400 COSY
solvent = cd3cn
sfrq/tof=128.328/837
dmm/dmf/dof=g/9600.000/0

lp/rp= -1000/80
at/d1 0.03 0.02
ct 80
lb = 17.1
gf = 0.004000
gfs = 0.008000
sp/wp= -5199/4336
sp1/wp1= -5199/4336
vs 1421.41
IS= 274944

-15 -25 ppm

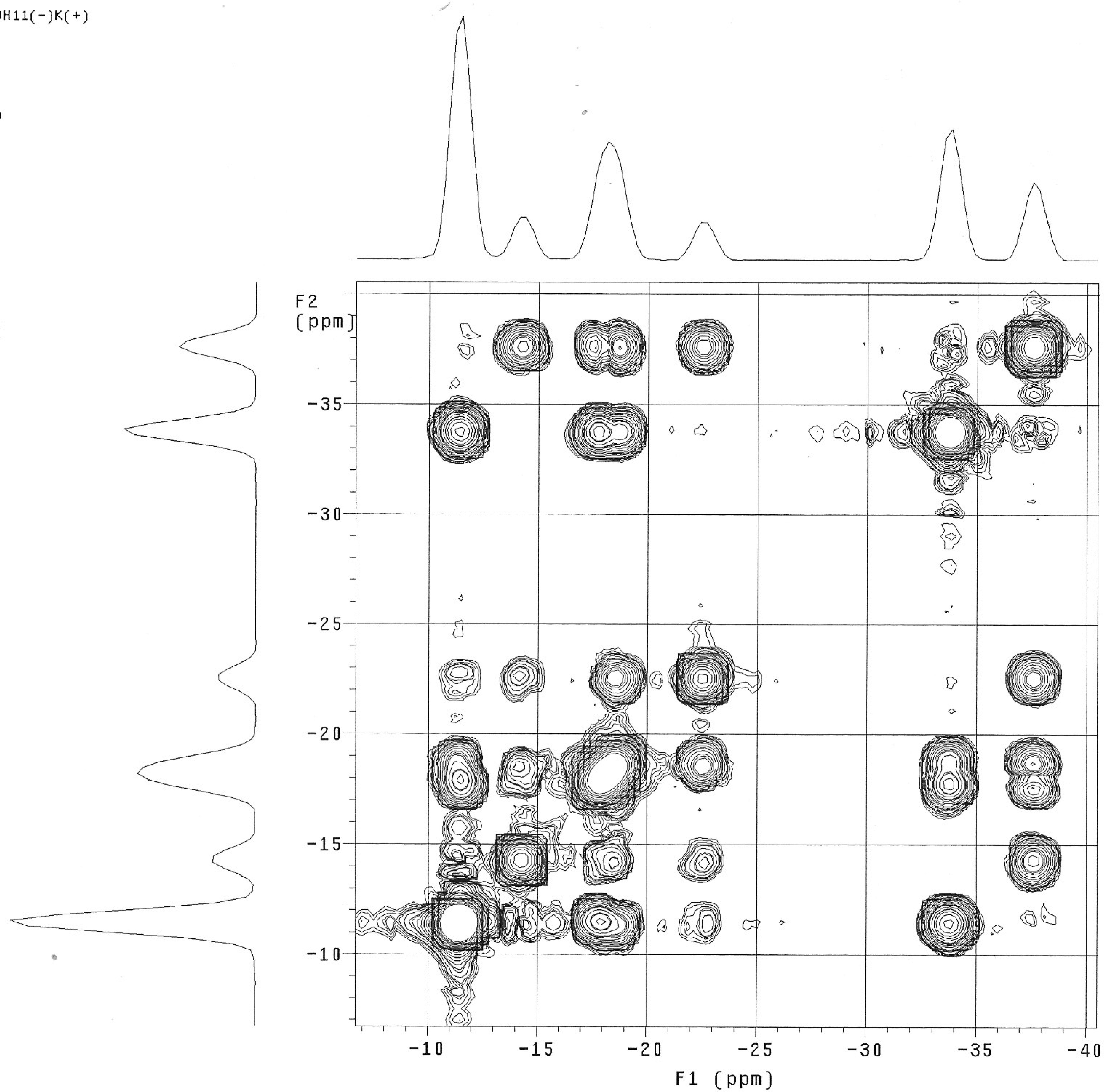

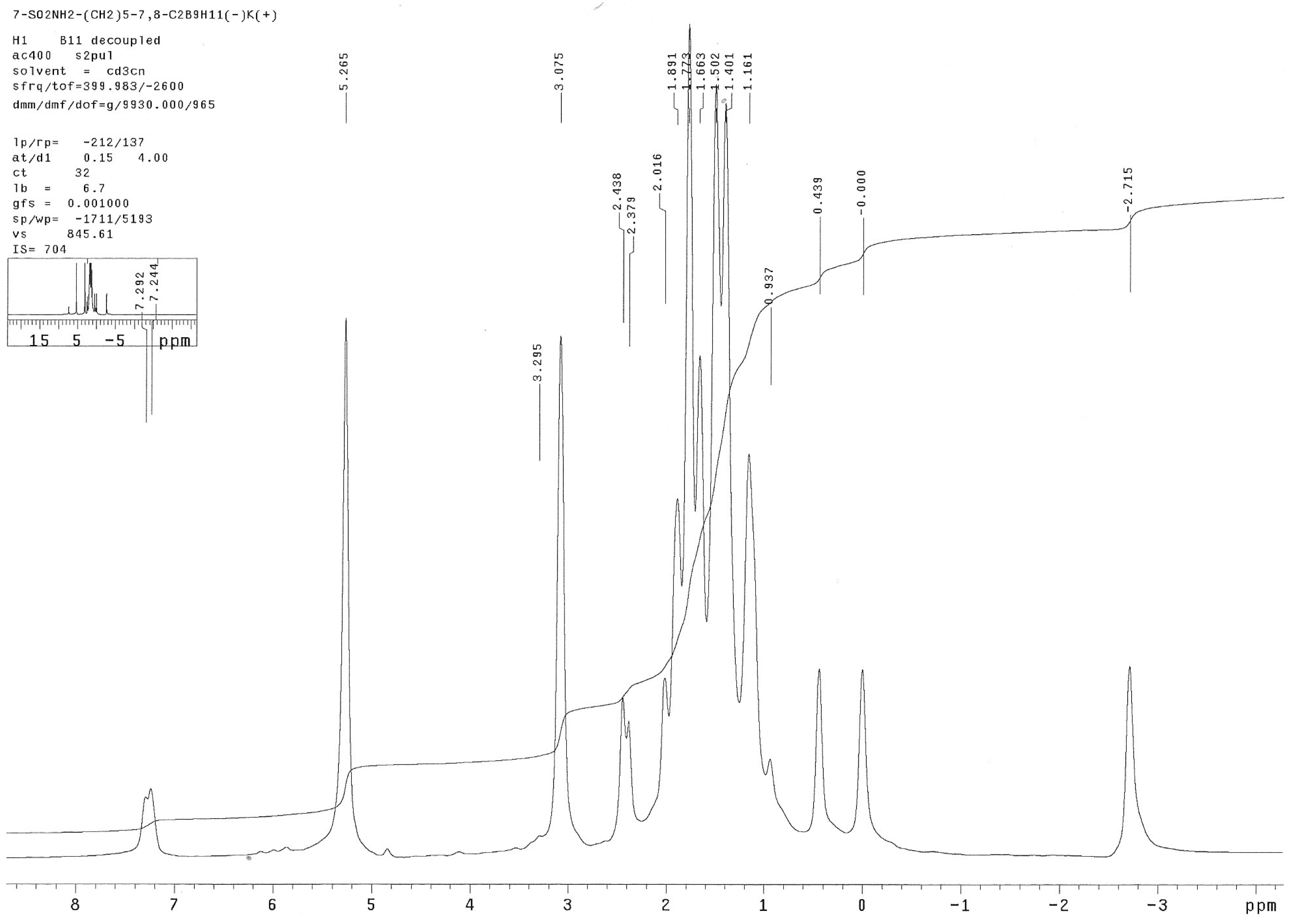
7-SO2NH2-(CH2)5-7,8-C2B9H11(-)K(+)
H1 B11 decoupled
ac400 s2pul
solvent = cd3cn
sfrq/tof=399.983/-2600
dmm/dmf/dof=g/9930.000/965
lp/rp= -212/137
at/d1 0.15 4.00
ct 32
lb = 6.7
gfs = 0.001000
sp/wp= -1711/5193
vs 845.61
IS= 704
15 5 -5 ppm
7.292
7.244
5.265
3.295
3.075
2.438
2.379
2.016
1.891
1.773
1.663
1.502
1.401
1.161
0.937
0.439
-0.000
-2.715
8 7 6 5 4 3 2 1 0 -1 -2 -3 ppm

7-SO2NH2-(CH2)5-7,8-C2B9H11(-)K(+)

H1 coupled
ac400 s2pul
solvent = cd3cn
sfrq/tof=399.983/-2600
dmm/dmf/dof=c/200.000/0

lp/rp= -325/-166
at/d1 2.00 1.00
ct 16
lb = 0.7
gfs = 0.001000
sp/wp= -1934/5193
vs 690.01
IS= 666

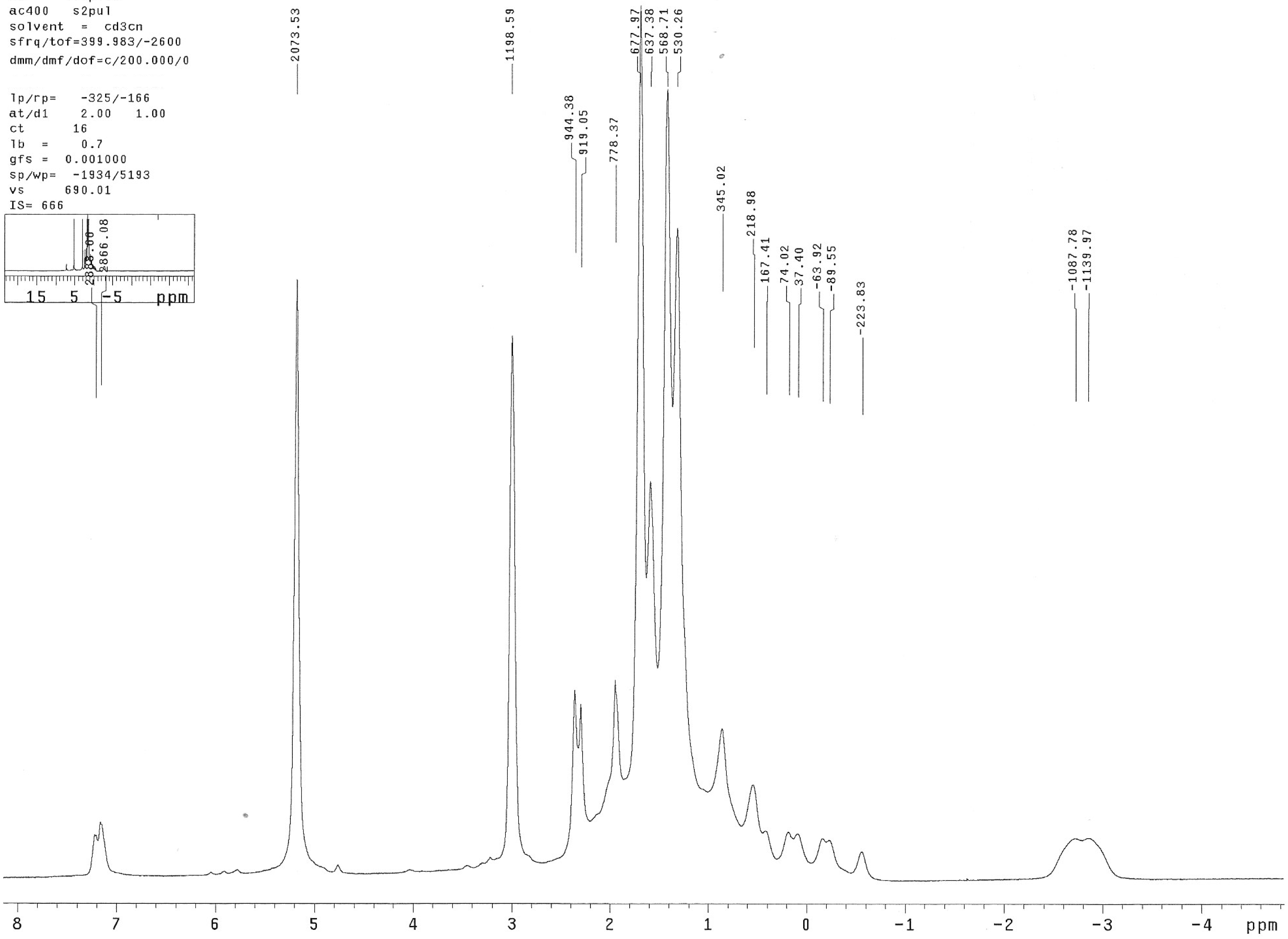

7-SO2NH2-(CH2)5-7,8-C2B9H11(-)K(+)

H1 B11 decoupled
ac400 s2pul
solvent = cd3cn
sfrq/tof=399.985/0
dmm/dmf/dof=c/200.000/-50000

lp/rp= -188/164
at/d1 0.20 4.00
ct 16
lb = 2.0
gfs = 0.000000
sp/wp= -1852/5493
vs 738.98
selective according to B11 ppm:
6 -37.643
5 -33.812
4 -22.536
3 -18.444
2 -14.281
1 -11.426
COUPLED SPECTRUM SUBSTRACTED
IS= 685

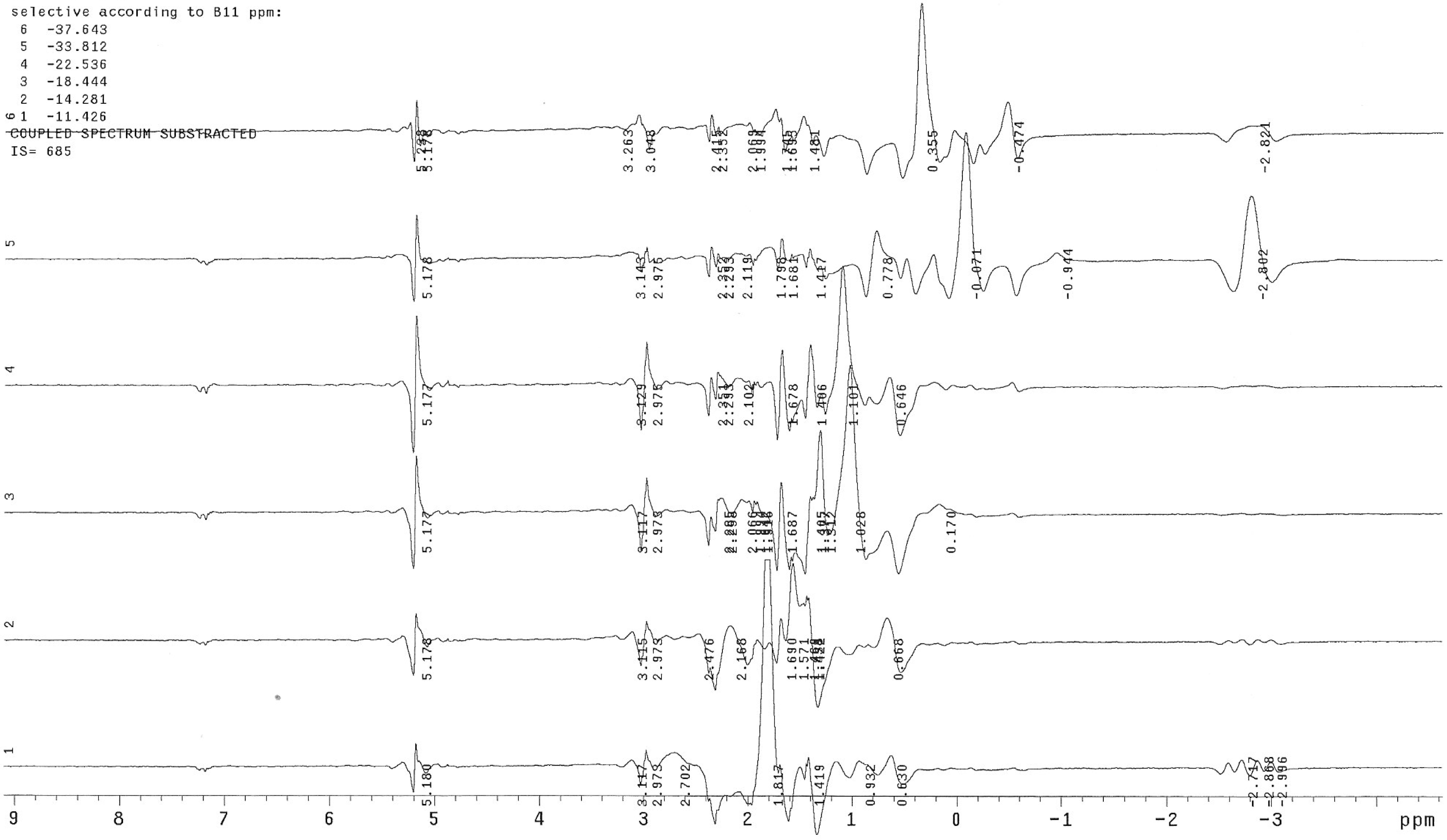

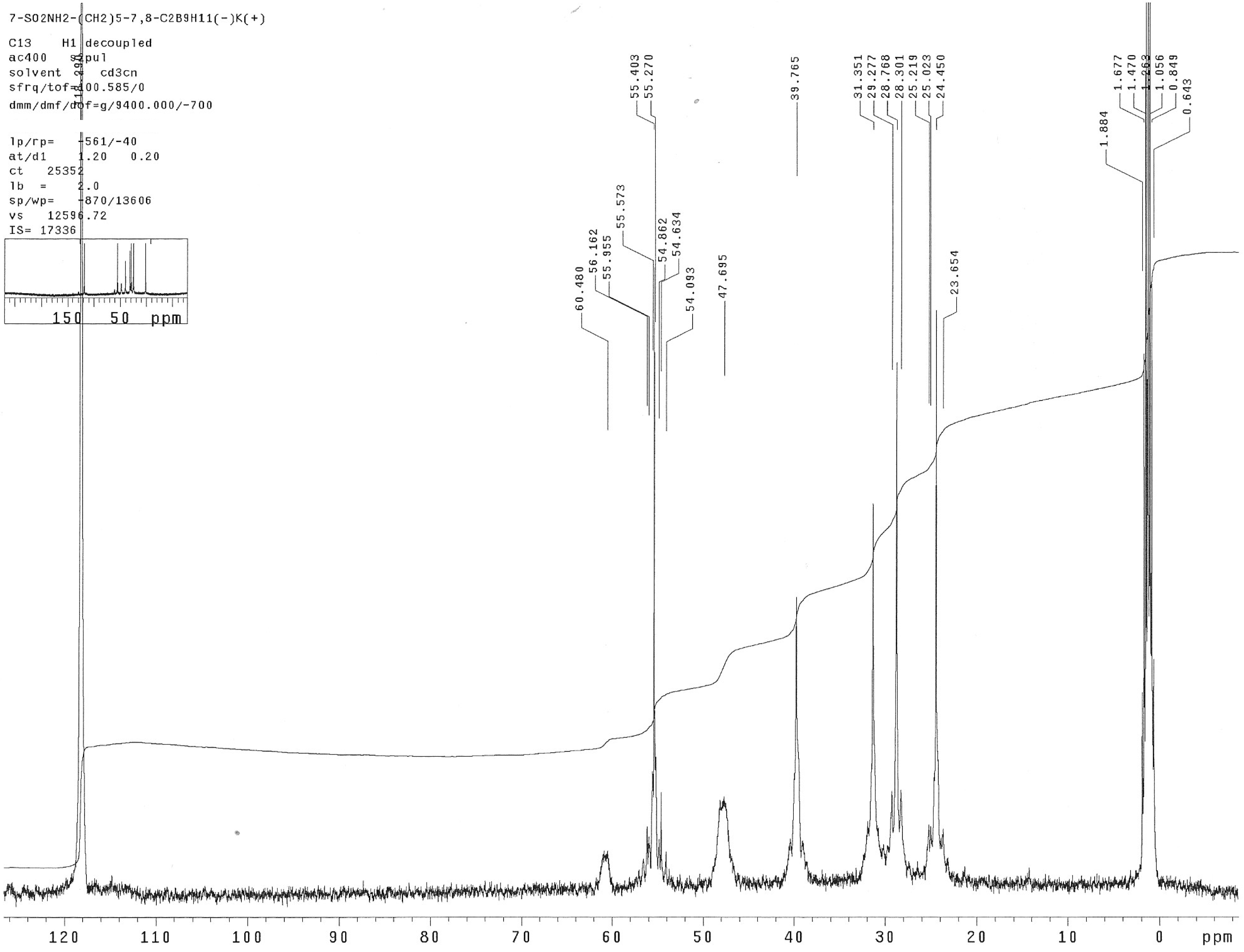
7-SO2NH2-(CH2)5-7,8-C2B9H11(-)K(+)
C13 H1 decoupled
ac400 s2pul
solvent = cd3cn
sfrq/tof=100.585/0
dmm/dmf/dof=g/9400.000/-700
lp/rp= -561/-40
at/d1 1.20 0.20
ct 25352
lb = 2.0
sp/wp= -870/13606
vs 12596.72
IS= 17336
150 50 ppm
55.403
55.270
39.765
31.351
29.277
28.768
28.301
25.219
25.023
24.450
1.677
1.470
1.263
1.056
0.849
0.643
1.884
55.573
56.162
55.955
54.862
54.634
60.480
54.093
47.695
23.654
120 110 100 90 80 70 60 50 40 30 20 10 0 ppm

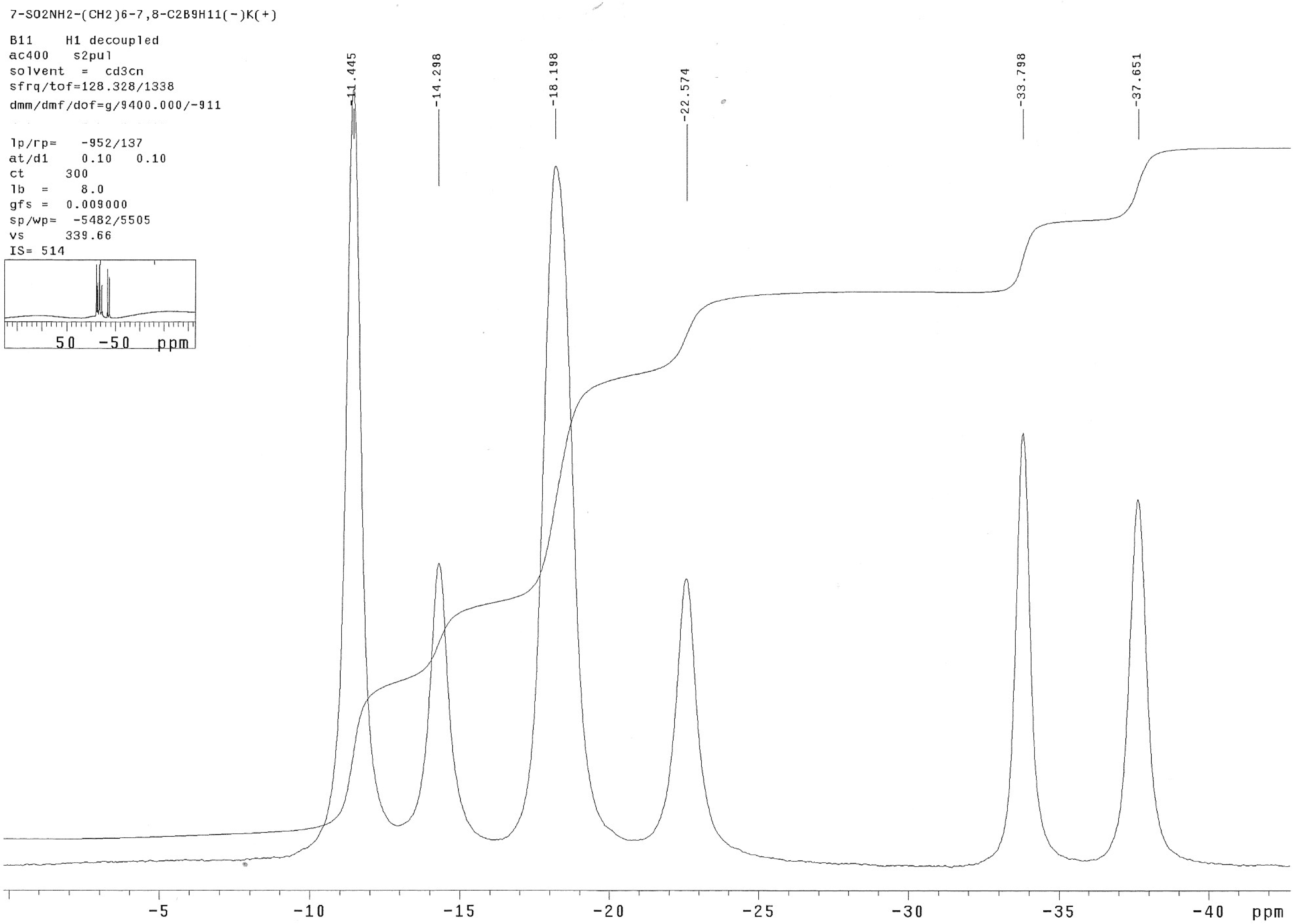
7-SO2NH2-(CH2)6-7,8-C2B9H11(-)K(+)
B11 H1 decoupled
ac400 s2pul
solvent = cd3cn
sfrq/tof=128.328/1338
dmm/dmf/dof=g/9400.000/-911
lp/rp= -952/137
at/d1 0.10 0.10
ct 300
lb = 8.0
gfs = 0.009000
sp/wp= -5482/5505
vs 339.66
IS= 514
50 -50 ppm
-11.445
-14.298
-18.198
-22.574
-33.798
-37.651
-5 -10 -15 -20 -25 -30 -35 -40 ppm

7-SO2NH2-(CH2)6-7,8-C2B9H11(-)K(+)

B11 coupled
ac400 s2pul
solvent = cd3cn
sfrq/tof=128.328/1338
dmm/dmf/dof=g/9200.000/0

lp/rp= -902/116
at/d1 0.10 0.10
ct 300
lb = 8.0
gfs = 0.003000
sp/wp= -5482/5505
vs 763.78
IS= 594

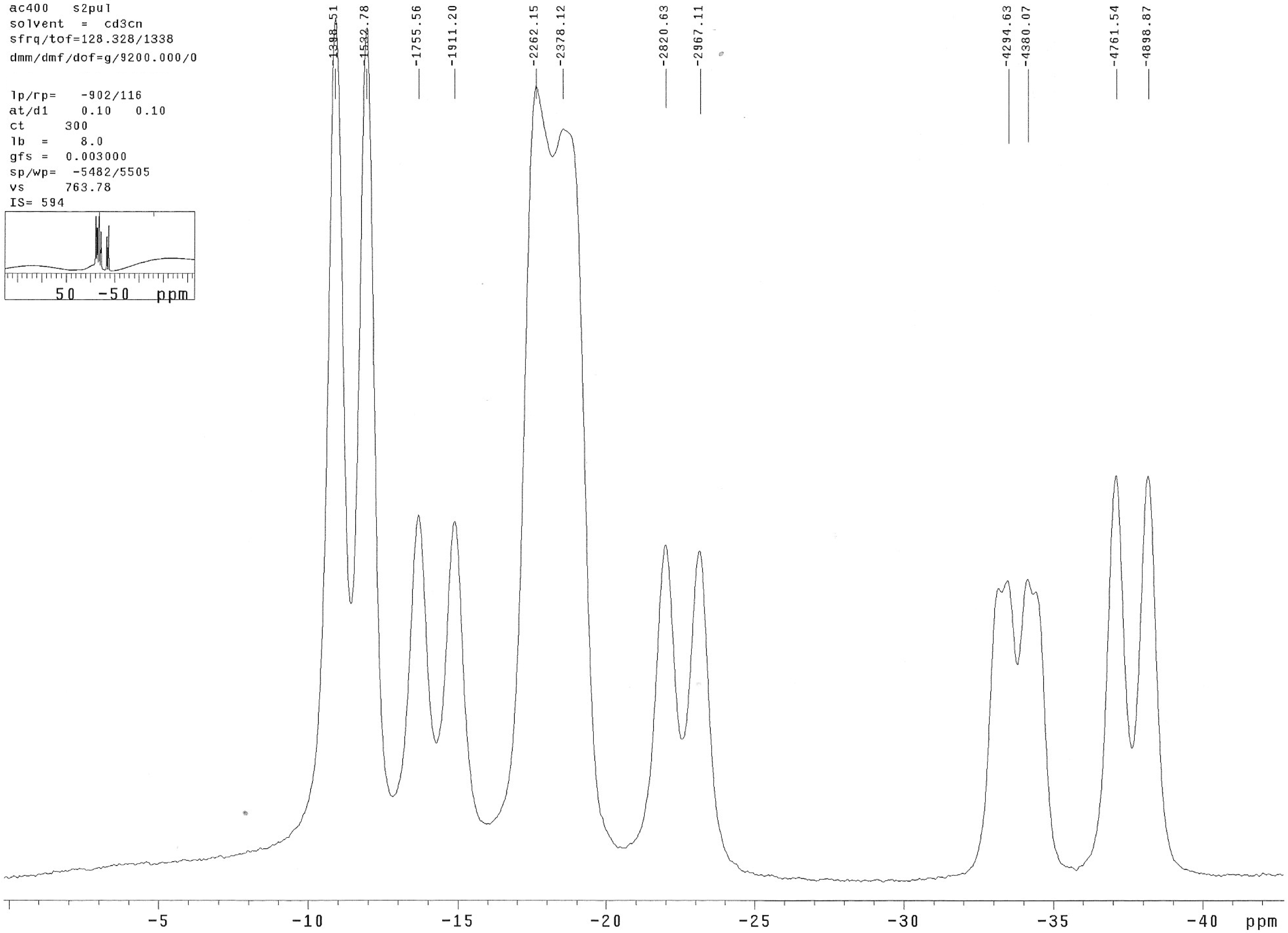

7-SO2NH2-(CH2)6-7,8-C2B9H11(-)K(+)

B11 H1 decoupled
ac400 COSY
solvent = cd3cn
sfrq/tof=128.328/811
dmm/dmf/dof=g/9600.000/0

lp/rp= -1000/80
at/d1 0.03 0.02
ct 108
lb = 17.1
gf = 0.004000
gfs = 0.008000
sp/wp= -5173/4233
sp1/wp1= -5173/4233
vs 1421.41
IS= 265500

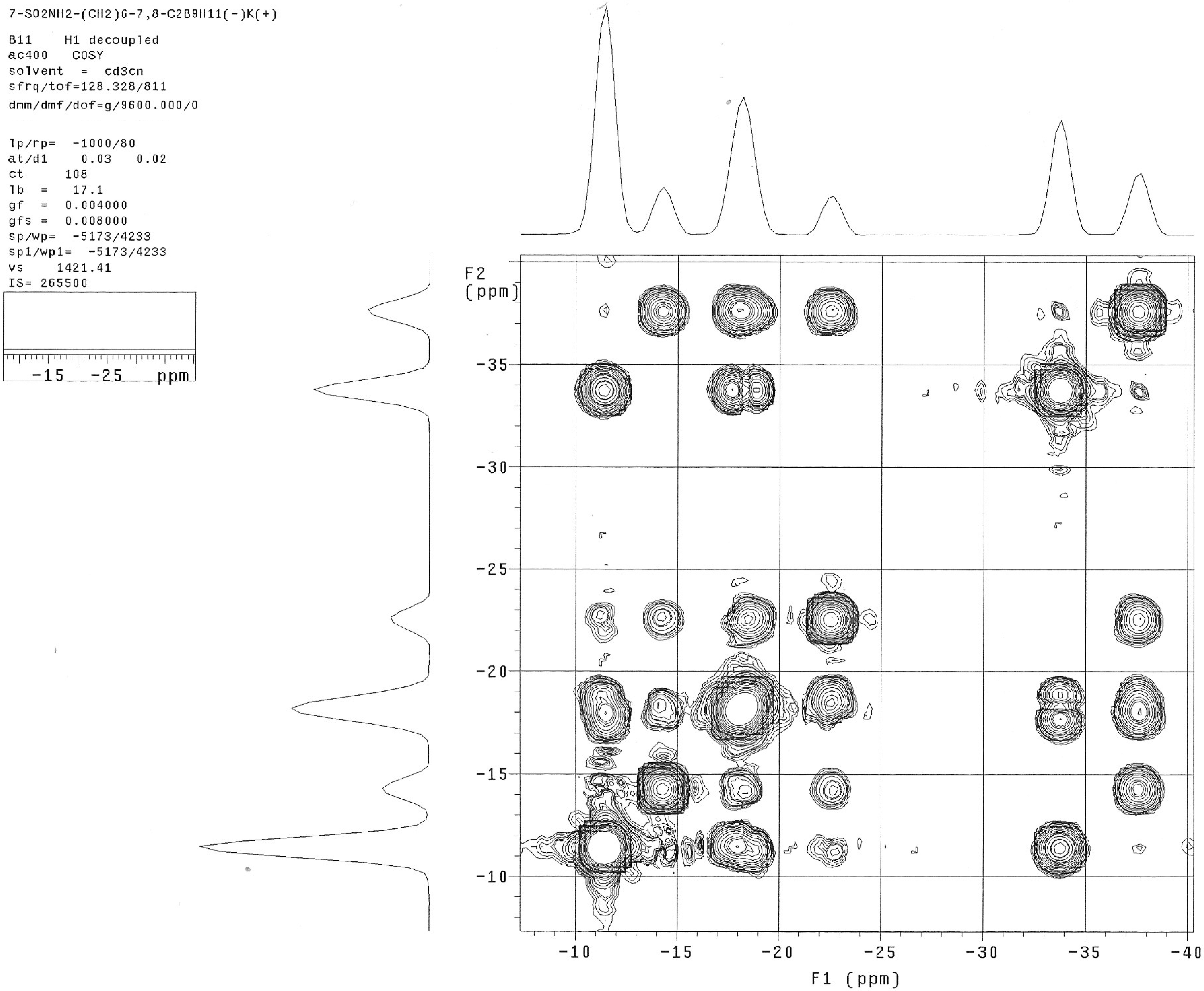

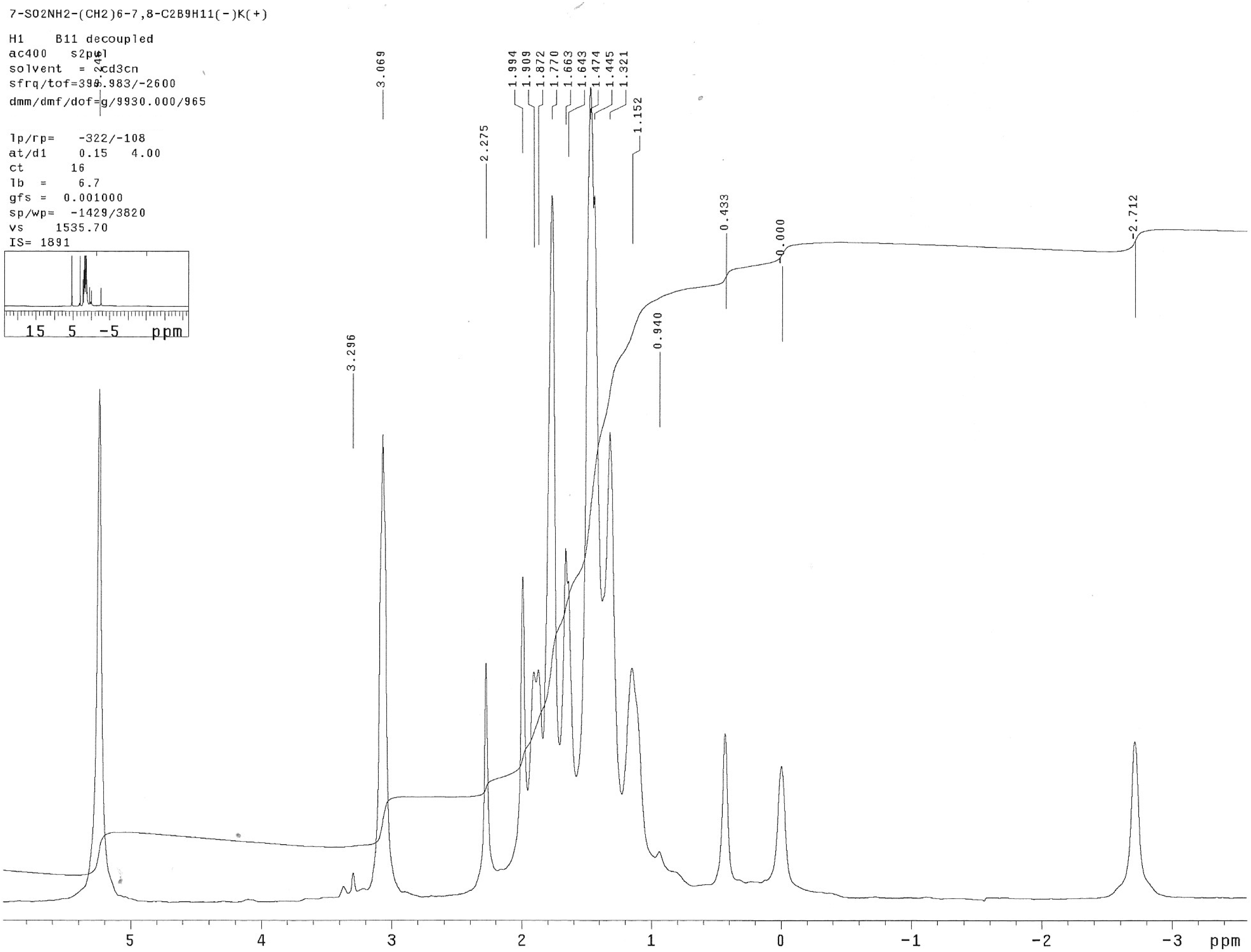

7-SO2NH2-(CH2)6-7,8-C2B9H11(-)K(+)
H1 B11 decoupled
ac400 s2pul
solvent = cd3cn
sfrq/tof=399.983/-2600
dmm/dmf/dof=g/9930.000/965
lp/rp= -322/-108
at/d1 0.15 4.00
ct 16
lb = 6.7
gfs = 0.001000
sp/wp= -1429/3820
vs 1535.70
IS= 1891
3.069
1.994
1.909
1.872
1.770
1.663
1.643
1.474
1.445
1.321
1.152
2.275
0.433
0.000
-2.712
0.940
3.296
ppm

7-SO2NH2-(CH2)6-7,8-C2B9H11(-)K(+)

H1 coupled
ac400 s2pul
solvent = cd3cn
sfrq/tof=399.953/-2600
dmm/dmf/dof=c/200.000/0

lp/rp= -279/-114
at/d1 2.00 1.00
ct 16
lb = 0.7
gfs = 0.001000
sp/wp= -1429/3820
vs 1392.31
IS= 1966

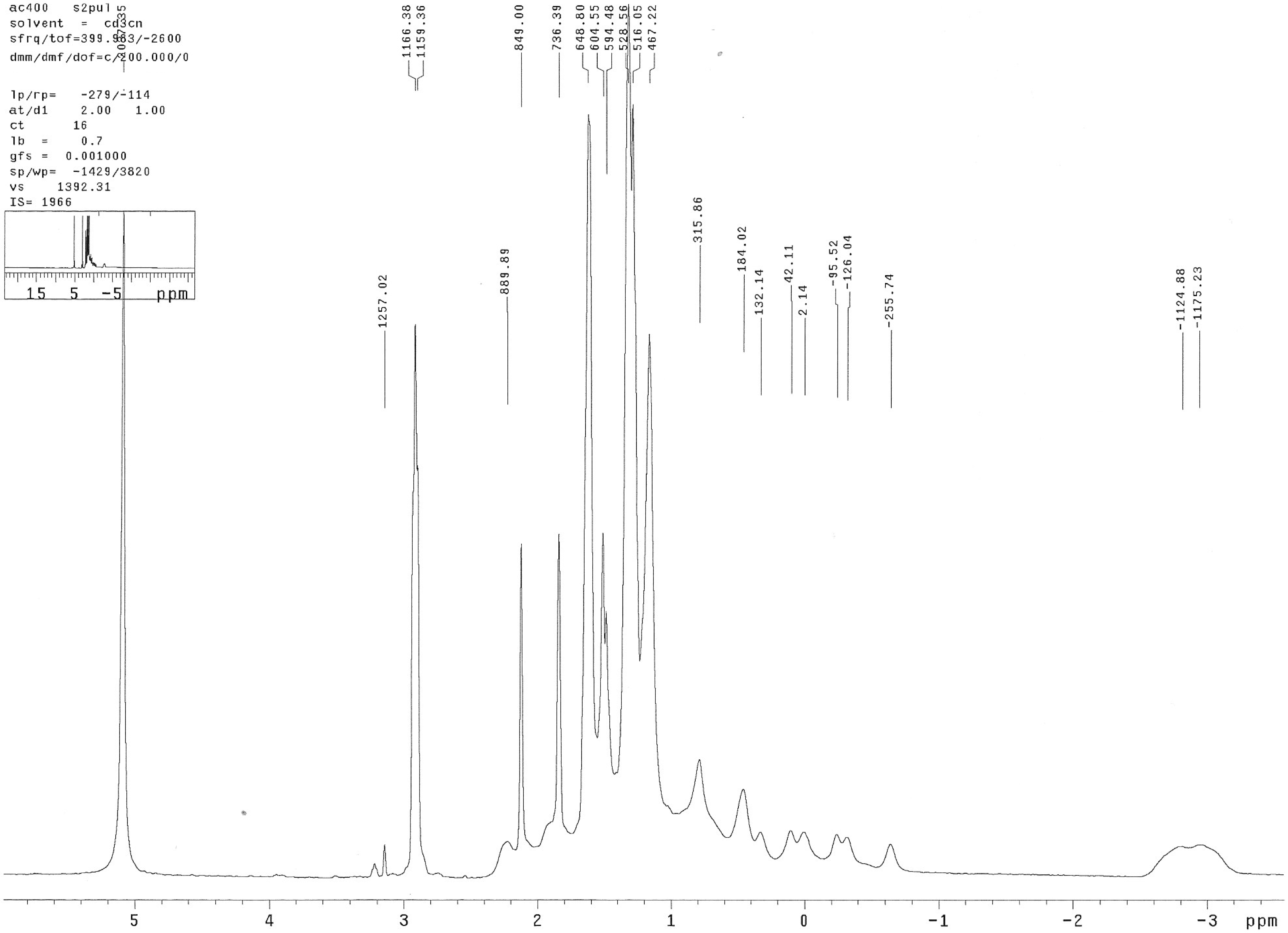

7-SO2NH2-(CH2)6-7,8-C2B9H11(-)K(+)

H1 B11 decoupled
ac400 s2pul
solvent = cd3cn
sfrq/tof=399.985/0
dmm/dmf/dof=c/200.000/-50000

lp/rp= -388/-55
at/d1 0.20 4.00
ct 16
lb = 2.0
sp/wp= -1804/4442
vs 753.38
selective according to B11 ppm:
6 -37.667
5 -33.812
4 -22.583
3 -18.206
2 -14.304
1 -11.449
COUPLED SPECTRUM SUBSTRACTED
IS= 1884

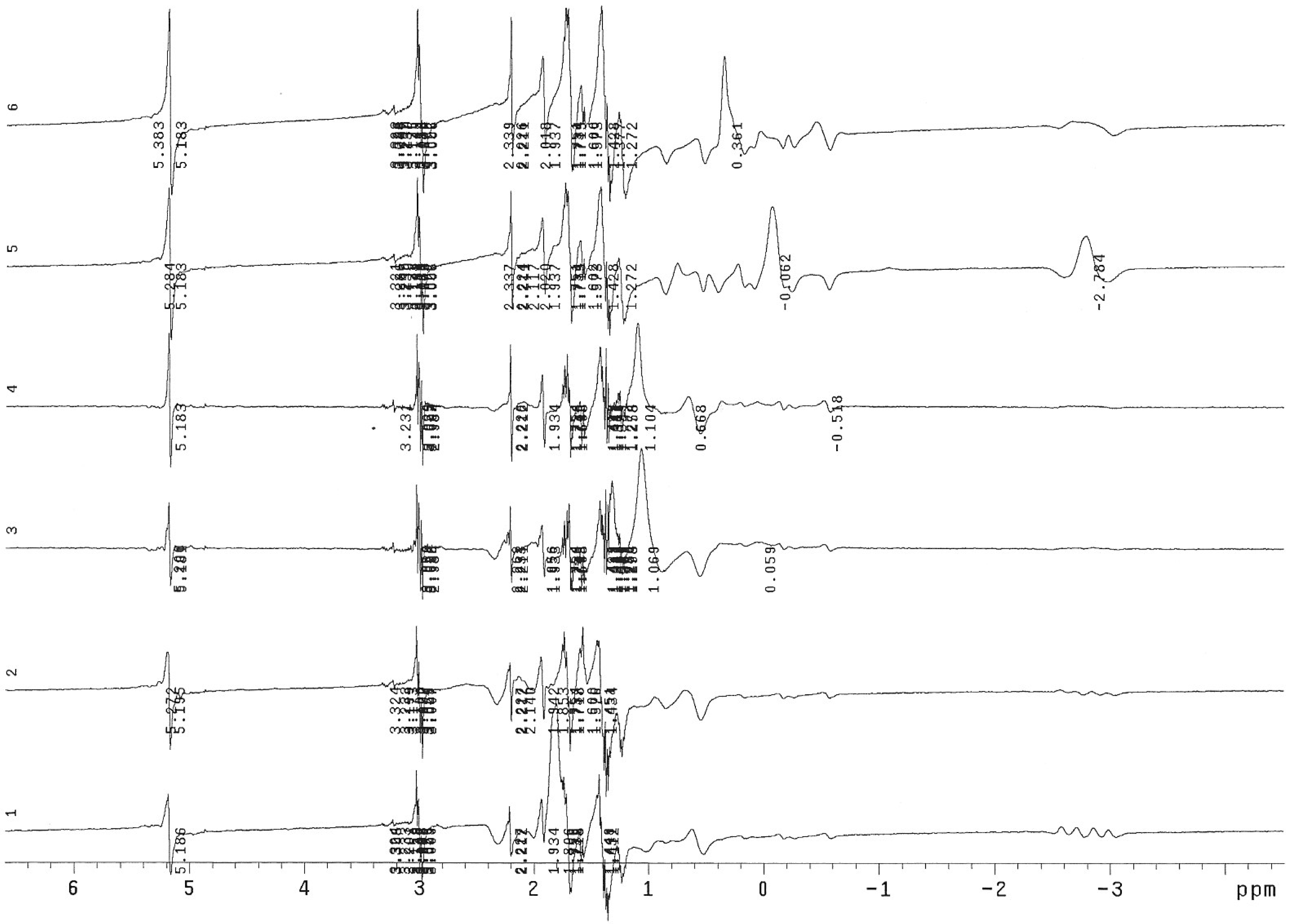

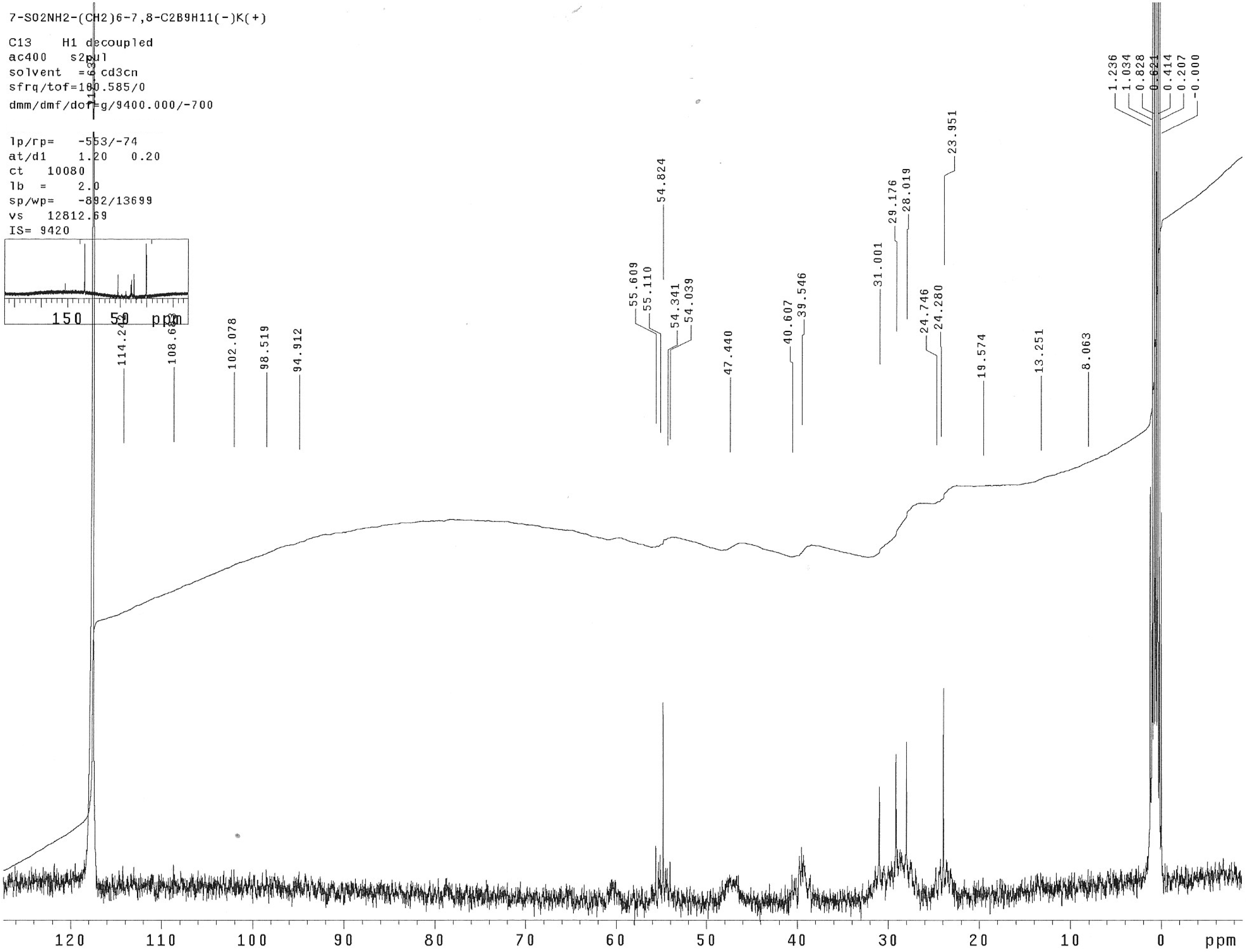
7-SO2NH2-(CH2)6-7,8-C2B9H11(-)K(+)
C13 H1 decoupled
ac400 s2pul
solvent = cd3cn
sfrq/tof=100.585/0
dmm/dmf/dof=g/9400.000/-700
lp/rp= -553/-74
at/d1 1.20 0.20
ct 10080
lb = 2.0
sp/wp= -892/13699
vs 12812.69
IS= 9420
ppm
